# Ethnicity and impact on the receipt of cognitive–behavioural therapy in people with psychosis or bipolar disorder: an English cohort study

**DOI:** 10.1136/bmjopen-2019-034913

**Published:** 2020-12-15

**Authors:** Rohan Michael Morris, William Sellwood, Dawn Edge, Craig Colling, Robert Stewart, Caroline Cupitt, Jayati Das-Munshi

**Affiliations:** 1 Division of Health Research, Lancaster University, Lancaster, UK; 2 Lancashire Care NHS Foundation Trust, Preston, UK; 3 Pennine Care NHS Foundation Trust, Greater Manchester, England; 4 Division of Psychology & Mental Health, School of Health Sciences, The University of Manchester, Manchester, UK; 5 Institute of Psychiatry, Psychology and Neuroscience, King’s College London, London, UK; 6 South London and Maudsley NHS Foundation Trust, London, UK; 7 Section of Epidemiology, Department of Health Service & Population Research, King’s College London, Institute of Psychiatry, London, UK

**Keywords:** schizophrenia & psychotic disorders, depression & mood disorders, mental health, adult psychiatry

## Abstract

**Objectives:**

(1) To explore the role of ethnicity in receiving cognitive–behavioural therapy (CBT) for people with psychosis or bipolar disorder while adjusting for differences in risk profiles and symptom severity. (2) To assess whether context of treatment (inpatient vs community) impacts on the relationship between ethnicity and access to CBT.

**Design:**

Cohort study of case register data from one catchment area (January 2007–July 2017).

**Setting:**

A large secondary care provider serving an ethnically diverse population in London.

**Participants:**

Data extracted for 30 497 records of people who had diagnoses of bipolar disorder (International Classification of Diseases (ICD) code F30-1) or psychosis (F20–F29 excluding F21). Exclusion criteria were: <15 years old, missing data and not self-defining as belonging to one of the larger ethnic groups. The sample (n=20 010) comprised the following ethnic groups: white British: n=10 393; Black Caribbean: n=5481; Black African: n=2817; Irish: n=570; and ‘South Asian’ people (consisting of Indian, Pakistani and Bangladeshi people): n=749.

**Outcome assessments:**

ORs for receipt of CBT (single session or full course) as determined via multivariable logistic regression analyses.

**Results:**

In models adjusted for risk and severity variables, in comparison with White British people; Black African people were less likely to receive a single session of CBT (OR 0.73, 95% CI 0.66 to 0.82, p<0.001); Black Caribbean people were less likely to receive a minimum of 16-sessions of CBT (OR 0.83, 95% CI 0.71 to 0.98, p=0.03); Black African and Black Caribbean people were significantly less likely to receive CBT while inpatients (respectively, OR 0.76, 95% CI 0.65 to 0.89, p=0.001; OR 0.83, 95% CI 0.73 to 0.94, p=0.003).

**Conclusions:**

This study highlights disparity in receipt of CBT from a large provider of secondary care in London for Black African and Caribbean people and that the context of therapy (inpatient vs community settings) has a relationship with disparity in access to treatment.

**Strengths and limitations of this study**

- A key strength of this study is that the data were from a near-complete case register of a large secondary care mental health service provider, which has a near monopoly on mental health provision in its catchment area.
- Published data are available on the tools used for extracting information about cognitive–behavioural therapy, which indicates high degrees of precision (95%) and sensitivity (96%).
- A limitation of this study is that it was not possible to assess access to other types of psychological intervention (eg, family therapy).
- This study was not able to assess the offer of therapy (only receipt); consequently, it is unclear if there are ethnic differences in whether therapy is offered to Black service users.

## Introduction

### Background

There are ethnic differences in the care pathways and treatments people with psychosis receive. Within the UK, people of Black Caribbean and Black African descent are more likely to: enter mental health services via forensic pathways and experience compulsory detention,^1^ receive medication by depot^2^ and be subject to community treatment orders.^3^ Black people with treatment-resistant schizophrenia are less likely to receive drug treatments in accordance with national guidelines, and Asian British people with a schizophrenia diagnosis are less likely to receive copies of their care plans.^2^ Treatment inequalities based on ethnicity have also been identified in other countries. For example, in the USA, people of African descent have less money spent on their healthcare through state-funded programmes^4^ and are less likely to receive medication associated with fewer side effects.^5^ In the Netherlands, ethnic minority groups are more likely to be compulsorily detained for treatment and less likely to be recommended for outpatient treatment.^6^

A prospective study in the UK found significant ethnic differences in Mental Health Act 2007 (MHA) assessments and detentions, with Black Africans having higher rates than any other ethnic group.^7^ However, when controlling for diagnosis, age, risk and social support, there were no significant ethnic differences in detention.^7^ Similarly, Singh *et al*^8^ found no significant differences between ethnic groups in MHA detention while controlling for variables such as risk and social support. These studies raise the possibility that treatment differences could be accounted for by ethnic differences in factors such as: self-harm and suicide attempt,^9^ psychosis symptom profiles,^10^ deprivation^11^ and substance use.^12^

UK national guidelines recommend cognitive–behavioural therapy (CBT) for the treatment and prevention of psychosis (CBTp), as CBTp has demonstrated robust evidence of its efficacy on service user outcomes.^13^ However, the National Audit of Schizophrenia found that CBTp was only offered to 39% of service users and accessed by 19% of service users.^14^ There are evidently barriers to accessing CBTp (eg, Hazell *et al*, Prytys *et al*^15 16^), although certain factors may increase referral to CBTp (eg, higher levels of positive symptoms^17^).

People from ethnic minority communities experience additional barriers to access and engagement with psychological therapy more generally.^18^ In the UK, people of Black Caribbean and Black African descent with psychosis are less likely to receive a talking therapy than their white British counterparts.^19–21^ A nationally representative survey of people with psychosis found that all ethnic minority groups (excluding those with mixed ethnicity) were less likely to be offered CBT, and Black service users were less likely to be offered family therapy.^2^ Similar findings have been demonstrated in international samples, where Black Americans with psychosis are less likely to receive a talking therapy than their white American counterparts.^22^ Nonetheless, research emanating from the UK (South London and Maudsley Improving Access to Psychological Therapies for people with severe mental illness (SLaM IAPT-SMI) Demonstration Site) has indicated that after CBTp has been offered there is no difference between a Black and Minority ethnic (BME) group and a non-BME group in engagement in CBTp.^23 24^

Engagement is a complex concept that requires the service provider being adequately engaging and the recipient to be adequately engaged. There are potentially many explanations of ethnic variations in access to and engagement with CBT. For example, ethnic minority communities have more coercive pathways into treatment (eg, Mann *et al*^1^), which may adversely influence the therapeutic relationship,^25^ and subsequently impact on engagement in treatment.^26^ Other barriers to engagement might include: lower socioeconomic status^27^; increased stigma in certain communities^28^; fear of service users by providers and fear of providers by serviceusers^29^; suspiciousness of mental health services and non-culturally appropriate therapy^30^; language barriers^31^; clinicians’ perceptions of religious and spiritual explanations for psychosis^32^; and institutional racism within mental health services.^33 34^

### Research questions and rationale

There is a lack of information about the extent of inequalities experienced by ethnic minority groups with serious mental illness, despite well-recognised adverse outcomes in certain minority groups. Furthermore, there is a paucity of information about the role that risk and symptom severity plays in treatment disparity (including access to psychological therapy) for ethnic minority groups. In order to address these gaps in knowledge, using all the case records from a large secondary care mental healthcare provider, this study set out to answer the following questions:

1. In people who have had a diagnosis of bipolar disorder (ICD-10 code F30-1) or psychosis (ICD-10 code F20-29 excluding F21), are there variations by ethnic group in receipt of either individual or group CBT after adjustment for differences in risk profiles and symptom severity?
2. Do ethnic group variations in receipt of CBT differ between contexts (eg, inpatient vs community settings) after adjustment for risk profiles and symptom severity?

## Method

### Study design and setting

The data, which were generated as part of routine care, were derived from clinical records from South London and Maudsley (SLaM) Trust. SLaM is a near-monopoly provider of secondary mental health services^35^ for a catchment of over 1.2 million residents in South London and has over 400 000 service user records.^36^ The SLaM catchment boroughs are not dissimilar from London as a whole in terms of age, education, gender and socioeconomic status.^36 37^ However, SLaM has a higher proportion of ethnic minority groups in comparison with England as whole.^36^ The (self-assigned) ethnicity population distribution recorded in the 2011 census for the SLaM catchment area is: 55.1% white, 24.7% Black, 10.8% Asian, 6.9% mixed ethnicity and 2.5% other.^36^ Even after adjustment for age, sex and ethnicity, areas within SLaM’s catchment have been shown to have a 2.2 times higher incidence of psychosis than the European average.^38^

This investigation used the Clinical Record Interactive Search (CRIS) tool^36^ to access an anonymised data set derived from SLaM’s electronic health records that comprise the Maudsley Biomedical Research Centre (BRC) Case Register. The BRC Case Register uses an opt-out mechanism, which is seldom used (circa n=4). Consequently, the sampling techniques employed ensure that persons who have not experienced good engagement with mental health services are still represented in the sample. Established in 2008, the CRIS system facilitates access and retrieval of anonymised clinical records. For a more in-depth description of how the data are stored, anonymised, and accessed see refs 36 37 39.

### Sample

Cases were included if they had received an ICD-10 diagnosis of a bipolar-related mental health problem (ie, manic episode (F30) and/or bipolar affective disorder (F31)) and were defined as having a bipolar disorder. The psychosis group included anyone with any of the following diagnoses: schizophrenia (F20), delusional disorder (F22), brief psychotic disorder (F23), shared psychotic disorder (F24), schizoaffective disorder (F25), other nonorganic psychotic disorders (F28) and unspecified nonorganic psychosis (F29).

No upper limit was set on age. Cases were excluded if: they were under the age of 15 years (a criterion that has been previously applied to this cohort^40^); they had a diagnosis of an organic/non-functional disorder; or there were missing data regarding marital status, ethnicity, Index of Multiple Deprivation (IMD) score, gender or age. To this end, only participants with complete data were included.

Due to limited numbers in some ethnic groups, cases were excluded if their recorded ethnicity did not belong to one of the following Office of National Statistics categories: Black African, Black Caribbean, Irish and white British.^41^ A group labelled ‘South Asian’ including individuals recorded as Indian, Pakistani or Bangladeshi was also included in the sample. This investigation used the same approach of defining and grouping ethnicity that has been applied to CRIS data previously.^40 42^

### Data retrieval

SLaM adopted fully electronic health records for all its services in 2006, including the importing of legacy data. The current data set includes records from 1 January 2007 up until the extraction date of 31 July 2017. Source clinical records contain information from structured closed question response boxes (eg, age) and free text. Automated natural language processing (NLP) algorithms (see ref 43) are used to determine the presence and prescribed ‘value’ of variables contained in free text.

Within the current investigation, NLP algorithms were used to provide supplementary information on diagnoses and CBT. Recording an ICD-10^44^ diagnosis within a structured field is mandatory within SLaM,^45^ supplemented by NLP to ascertain diagnoses recorded in free-text sources, for example, clinical notes.^36 45^ Another NLP algorithm has been developed to identify case notes that document a CBT session,^19^ again supplementing information within structured fields and achieving in combination a positive predictive value of 95% and a sensitivity of 96%.^19^

### Demographic, clinical and treatment data extracted and operationalised

Demographic data retrieved included gender, marital status, ethnicity and age. All of the demographic data was retrieved at the point of data extraction (31 July 2017), for example, the participants’ age on the 31 of July 2017. From lower super output area of residence, a standard national geographic unit containing approximately 1500 residents, area level deprivation was calculated from the IMD.^46^ Multiple area level assessments contribute to seven subscales (income deprivation; employment deprivation; education, skills and training deprivation; health deprivation and disability; crime; barriers to housing and services; and living environment deprivation) that form the IMD. Scores on the IMD were split into deciles within the current sample.

The algorithm within the SLaM clinician interface ensures that structured risk assessments are completed when risk information is noted. We developed an assessment of severity and risk based on previous approaches used with this dataset.^47^ To this end, we retrieved information from structured risk assessments pertaining to: history of violence, history of ‘non-adherence’, history of suicide attempt, perceived lethal means used in suicide attempt, current plans to end life, expression of suicidal ideation, expressed feelings of hopelessness, expressed high levels of subjective distress and expressed feelings of having no control. We also retrieved information about previous: substance use disorder diagnosis (ICD code F1), inpatient admissions, treatment under the MHA, Accident and Emergency (A&E) attendance (for mental health problems), referral to assertive outreach, referral to the crisis team and forensic history.

**Figure 1:**
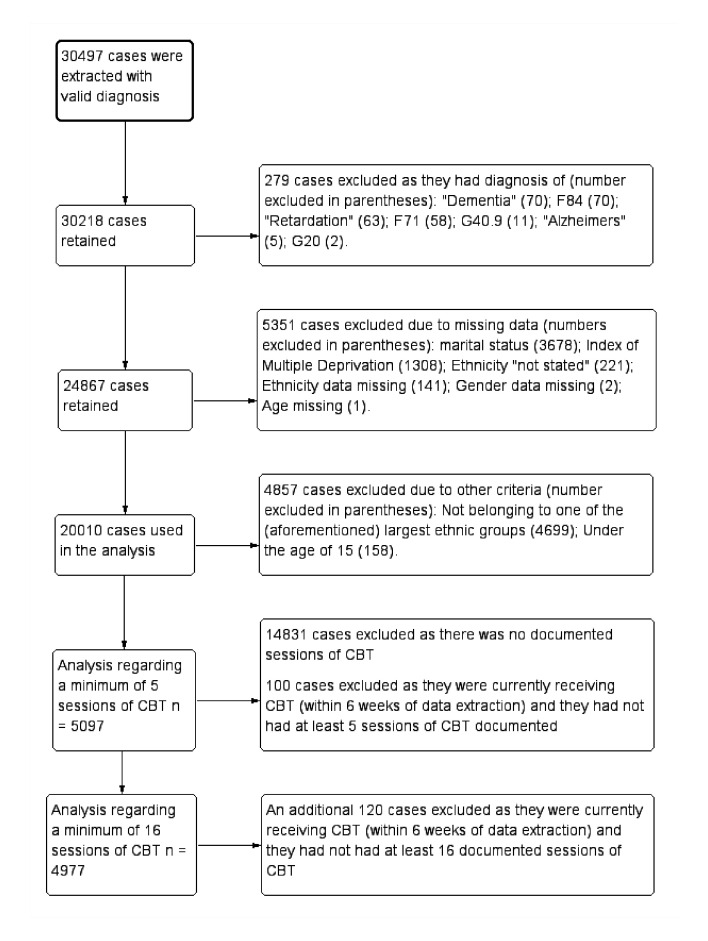
Demonstrating the flow of included cases. CBT, cognitive–behavioural therapy.

We retrieved data about the CBT session regarding: whether the service user was an inpatient or outpatient at the time of contact; whether the contact was face to face or remote (eg, via telephone); and whether the contact was in a one-to-one or group session. In line with national standard guidelines definition of access,^48^ the current investigation assessed whether participants had at least one documented session of CBT. National Institue for Health and Clinical Excellence (NICE) guidelines for psychosis recommend that CBT is delivered ‘over at least 16 planned session (sic)’ (13, p. 589). NICE guidelines for bipolar disorder recommend that a depressive episode should be treated with between 16 and 20 sessions of CBT.^49^ Consequently, a 16-session criterion was also adopted as a more stringent definition of a course of CBT. Jolley and colleagues^23^ operationalised CBT completion as at least five sessions. Supplementary analyses were conducted using this less stringent definition of the completion of CBT treatment. Analyses of the 5 and 16 session criteria were restricted to participants who had at least one documented session of CBT (n=5197). Participants were also excluded from analyses regarding the 5 and 16 session criteria if they were currently receiving CBT at data extraction and had not received a minimum of 5 or 16 sessions of CBT, which resulted in 100 and 220 participants being excluded respectively (see figure 1). CBT that was currently ongoing was defined as anyone who had a CBT session in the 6 weeks prior to data extraction.

### Patient and public involvement

This specific project was reviewed, commented on and approved by the CRIS Oversight Committee, which is chaired by a service user representative. Furthermore, the development of the CRIS system was informed by consultation with service users.^39^

### Analysis

Logistic regression models were built using multivariable procedures in Stata V.12. Models were adjusted for demographic data (gender, age, IMD and marital status), diagnoses (psychosis/bipolar disorder) and risk/severity variables (as described previously). Analyses are presented as: crude associations; adjustments for demographic data and diagnosis (step 1); and adjustments for demographic data, diagnosis and the risk/severity variables (step 2).

## Results

### Descriptive statistics

A total of 5351 cases were excluded due to missing data relating to marital status (n=3678), Index of Multiple Deprivation (n=1308), ethnicity (n=362), gender (n=2) and age (n=1). The final sample consisted of 20 010 cases; figure 1 displays the flow of cases through the study.

The majority of cases were white British (n=10 393, 51.9%); the next largest ethnic group were Black Caribbean people who made up 27.4% of the sample (n=5481). There were more male cases (n=10 457, 52.3%) than female, and the majority were single (n=17 097, 85.4%). Table 1 summarises the demographic and diagnosis data (at the time of data extraction) with relevant proportions for each ethnic group. Further information on treatment, risk and severity including items from the structured risk assessment can be found in online supplemental table 1.

10.1136/bmjopen-2019-034913.supp1 Supplementary data

**Table 1:** Information on baseline demographics and diagnoses and their relevant proportions for each ethnic group

	White British		Irish		Black African		Black Caribbean		South Asian		Total		χ^2^ (df)	P value
	N	%	N	%	N	%	N	%	N	%				
Ethnicity	10 393	52	570	3	2817	14	5481	27	749	4	20 010			
Female	5070	49	269	47	1350	48	2497	46	367	49	9553	48		
Male	5323	51	301	53	1467	52	2984	54	382	51	10 457	52	16	<0.01
Index of Multiple Deprivation														
1	1489	14	42	7	70	3	199	4	59	8	1859	9	1000 (36)*	<0.001
2	1160	11	53	9	165	6	456	8	92	12	1926	10		
3	1133	11	62	11	195	7	536	10	87	12	2013	10		
4	1041	10	53	9	284	10	542	10	86	12	2006	10		
5	980	9	58	10	302	11	584	11	82	11	2006	10		
6	920	9	62	11	327	12	654	12	69	9	2032	10		
7	933	9	60	11	326	12	617	11	80	11	2016	10		
8	919	8.8	59	10	407	15	651	12	54	7	2090	10		
9	867	8.3	60	11	379	14	646	12	64	9	2016	10		
10	951	9.2	61	11	362	13	596	11	76	10	2046	10		
Single marital status	8784	85	486	85	2300	82	5035	92	492	66	17 097	85	456(4)*	<0.001
In relationship	1609	16	84	15	517	18	446	8	257	34	2913	15		
Age: median (IQR)	49 (26.9)		56 (28.8)		43 (18.8)		46 (22.3)		47 (26.2)		48 (24.5)		451(4)*	<0.001
Psychosis†	6516	63	366	64	2435	86	4617	84	563	75	14 497	73	1200(4)*	<0.001
Bipolar‡	3877	37	204	36	382	14	864	16	186	25	5513	28		
Lifetime comorbid substance use diagnosis	1675	16	140	25	292	10	865	16	53	7	3025	15.1	94 (4)*	<0.001

*Kruskal-Wallis H non-parametric test for ranked data used to determine the χ^2^ value

†Psychosis=diagnosis of schizophrenia, delusional disorder, brief psychotic disorder, shared psychotic disorder, schizoaffective disorder, other non-organic psychotic disorders or unspecified non-organic psychosis.

‡Bipolar=diagnosis of a manic episode or bipolar affective disorder.

1, least deprived; 10, most deprived.

Just over a quarter of the sample (26.0%, n=5197) had a documented session of CBT in the study period. The median number of sessions of CBT was 5 (IQR 13). Considering all CBT sessions documented, most were delivered face to face at a ratio of approximately 30 face-to-face sessions for every one remote (eg, telephone) session delivered and as individual rather than group sessions at a ratio of approximately 17:1. Of the people who had received CBT, 30% had their first ever (documented) session as an inpatient, 55.4% had ≥5 sessions and 25.8% had received ≥16 sessions. Further information about CBT can be found in online supplemental table 2.

### Ethnicity and reported receipt of CBT as an inpatient or outpatient

Table 2 displays the unadjusted and adjusted ORs for having a reported session of CBT in relation to ethnicity and covariates. The final adjusted model indicated that the Black African group were significantly less likely to receive CBT than the white British group (OR 0.73, 95% CI 0.66 to 0.82, p<0.001), after risk indicators were taken into account. In the adjusted model, several factors related to risk and severity were independently associated with increased likelihood of reported receipt of CBT, including lifetime inpatient admission, history of non-adherence, history of suicide attempt, lethal means used in suicide attempt, suicidal ideation, feelings of hopelessness, high levels of distress, no feelings of control and referral to the crisis team. However, a history of a substance misuse disorder diagnosis and plans to end life were associated with a decreased likelihood of reported receipt of CBT.

**Table 2:** Crude and adjusted associations from logistic regression models for at least one recorded session of CBT (inpatient or outpatient)

Variable	N	OR (95% CI)		
		Crude associations	Step 1	Step 2
Ethnicity				
White British	10 393	Reference group		
Irish	570	1.00 (0.82 to 1.21)	1.12 (0.91 to 1.36)	1.05 (0.85 to 1.29)
Black African	2817	1.06 (0.97 to 1.17)	0.96 (0.87 to 1.06)	0.73 (0.66 to 0.82)***
Black Caribbean	5481	1.29 (1.20 to 1.39)***	1.20 (1.11 to 1.30)***	0.93 (0.86 to 1.02)
South Asian	749	0.99 (0.83 to 1.18)	0.97 (0.82 to 1.16)	0.93 (0.77 to 1.12)
Gender				
Female	9553	Reference group		
Male	10 457	0.89 (0.84 to 0.95)***	0.84 (0.78 to 0.89)***	0.84 (0.78 to 0.90)***
Age (years)		0.98 (0.98 to 0.99)***	0.98 (0.98 to 0.99)***	0.99 (0.98 to 0.99)***
Area level deprivation				
IMD decile (per 10th)		1.01 (1.00 to 1.02)	1.01 (0.99 to 1.02)	0.99 (0.98 to 1.00)
Marital status				
In relationship	2913	Reference group		
Single	17 097	1.23 (1.12 to 1.35)	1.08 (0.98 to 1.19)	1.07 (0.97 to 1.18)
Diagnosis				
Psychosis	14 497	Reference group		
Bipolar affective disorder	5513	0.94 (0.88 to 1.01)	0.93 (0.86 to 1.00)	1.00 (0.93 to 1.09)
Comorbid substance misuse				
No previous substance misuse diagnosis	16 985	Reference group		
Lifetime comorbid substance misuse diagnosis	3025	1.31 (1.20 to 1.42)***		0.85 (0.77 to 0.93)***
Admission				
No previous admission	10 593	Reference group		
Inpatient admission ever	9417	3.20 (2.99 to 3.42)***		1.76 (1.58 to 1.95)***
Treatment under the Mental Health Act (MHA)				
Never treated under MHA	12 904	Reference group		
Ever treated under MHA	7106	2.54 (2.38 to 2.71)***		0.96 (0.87 to 1.07)
Structured risk assessment items†				
History of violence	6216	2.31 (2.16 to 2.47)***		1.09 (1.00 to 1.20)
Difficulty managing physical health	3622	1.74 (1.61 to 1.88)***		0.97 (0.88 to 1.07)
History of non-adherence	6425	2.55 (2.39 to 2.73)***		1.27 (1.16 to 1.39)***
History of suicide attempt	3758	2.83 (2.63 to 3.05)***		1.36 (1.22 to 1.53)***
Lethal means used in suicide attempt	2026	2.65 (2.41 to 2.91)***		1.04 (1.22 to 1.53)***
Plans to end life	863	2.62 (2.29 to 3.01)***		0.82 (0.69 to 0.96)*
Suicidal ideation	2041	3.23 (2.94 to 3.55)***		1.24 (1.10 to 1.41)***
Feelings of hopelessness	2850	3.06 (2.82 to 3.32)***		1.24 (1.11 to 1.40)***
High level of distress	4666	3.24 (3.02 to 3.47)***		1.53 (1.40 to 1.68)***
No feelings of control	2972	3.03 (2.79 to 3.28)***		1.22 (1.09 to 1.36)***
Referred/seen by other team				
Never referred to crisis team	13 504	Reference group		
Ever referred to the crisis team	6506	2.96 (2.77 to 3.16)***		1.69 (1.57 to 1.83)***
Never seen at A&E‡	13 389	Reference group		
Ever seen at A&E‡	6621	1.69 (1.58 to 1.80)***		0.97 (0.90 to 1.04)
Never referred to assertive outreach	18 977	Reference group		
Ever referred to assertive outreach	1033	1.51 (1.32 to 1.72)***		0.94 (0.81 to 1.09)
Forensic history				
No forensic history reported	18 137	Reference group		
Forensic history reported	1873	1.70 (1.53 to 1.88)***		1.07 (0.96 to 1.20)

Step 1: adjusted for ethnicity+gender+age+IMD decile+marital status+diagnosis: psychosis/bipolar.

Step 2: adjusted for ethnicity+gender+age+IMD decile+marital status+diagnosis: psychosis/bipolar+substance use diagnosis+inpatient admittance+treated under the MHA+structured risk assessment items (entered separately)+referred to crisis team+treated at A&E+referred to assertive outreach+forensic history.

*P<0.05;**p<0.01; ***p<0.001.

†For brevity, reference groups are omitted. Reference groups are a non-affirmative response to the item. The n for the reference group is the number of people included in the analysis (n=20 010) – the number of people with an affirmative response.

‡Seen at A&E due to mental health emergency.

1, least deprived; 10, most deprived; CBT, cognitive–behavioural therapy; IMD, Index of Multiple Deprivation.

#### Ethnicity and a minimum of 16 CBT sessions

Table 3 displays the unadjusted and adjusted ORs of receiving a minimum of 16 sessions of CBT in relation to ethnicity and covariates. The adjusted model indicated that the Black Caribbean group were significantly less likely to receive a minimum of 16 sessions of CBT than the white British group (OR 0.83, 95% CI 0.71 to 0.98, p=0.03). The model also indicated that receiving the first session of CBT as an inpatient was associated with decreased odds of having at least 16 sessions of CBT (OR 0.35, 95% CI 0.29 to 0.42, p<0.001) and some of the indicators of risk increased the odds of receiving CBT (history of suicide attempt, reported high levels of distress and lifetime referral to crisis team). However, several factors associated with increased odds of ever receiving a documented session of CBT (table 2) were not significantly associated with having a minimum of 16 documented sessions (ie, lifetime inpatient admittance, history of non-adherence, lethal means used in suicide attempt, reported suicidal ideation, reported feelings of hopelessness and reported feelings of a lack of control).

**Table 3:** Crude and adjusted associations from logistic regression models for at least 16 recorded sessions of CBT

Variable	N	OR (95% CI)		
		Crude associations	Step 1	Step 2
Ethnicity				
White British	2456	Reference group		
Irish	137	1.03 (0.70 to 1.50)	1.02 (0.70 to 1.50)	1.05 (0.71 to 1.55)
Black African	682	0.78 (0.64 to 0.95)*	0.77 (0.63 to 0.95)*	0.86 (0.69 to 1.06)
Black Caribbean	1524	0.77 (0.67 to 0.90)**	0.76 (0.65 to 0.89)**	0.83 (0.71 to 0.98)*
South Asian	178	0.98 (0.70 to 1.38)	0.99 (0.72 to 1.39)	1.03 (0.73 to 1.47)
Gender				
Female	2485	Reference group		
Male	2492	0.99 (0.87 to 1.12)	0.98 (0.86 to 1.11)	1.05 (0.91 to 1.20)
Age (years)		1.00 (1.00 to 1.01)	1.00 (1.00 to 1.01)	1.00 (1.00 to 1.01)
Area level deprivation				
IMD decile (per 10th)		0.99 (0.97 to 1.01)	1.00 (0.97 1.02)	0.99 (0.97 to 1.01)
Marital status				
In relationship	639	Reference group		
Single	4338	1.07 (0.88 to 1.29)	1.11 (0.91 to 1.36)	1.21 (0.98 to 1.48)
Diagnosis				
Psychosis	3645	Reference group		
Bipolar affective disorder	1332	0.95 (0.83 to 1.10)	0.90 (0.77 to 1.04)	0.86 (0.74 to 1.01)
Comorbid substance misuse				
No previous substance misuse diagnosis	4090	Reference group		
Lifetime comorbid substance misuse diagnosis	887	0.81 (0.69 to 0.97)*		0.79 (0.66 to 0.96)*
Admission				
No previous admission	1622	Reference group		
Inpatient admission ever	3355	0.74 (0.65 to 0.85)***		1.06 (0.86 to 1.31)
Treatment under Mental Health Act (MHA)				
Never treated under MHA	2429	Reference group		
Ever treated under the MHA	2548	0.70 (0.61 to 0.79)***		0.86 (0.71 to 1.05)
Structured risk assessment items†				
History of violence	2234	0.80 (0.71 to 0.91)**		0.93 (0.78 to 1.10)
Difficulty managing physical health	1237	0.94 (0.81 to 1.09)		1.01 (0.85 to 1.20)
History of non-adherence	2382	0.83 (0.73 to 0.95)**		0.91 (0.77 to 1.08)
History of suicide attempt	1589	1.39 (1.22 to 1.59)***		1.33 (1.09 to 1.61)**
Lethal means used in suicide attempt	887	1.36 (1.16 to 1.60)***		1.01 (0.80 to 1.27)
Reported plans to end life	382	1.54 (1.23 to 1.92)***		1.33 (1.01 to 1.73)*
Suicidal ideation	961	1.38 (1.18 to 1.61)***		1.10 (0.89 to 1.35)
Feelings of hopelessness	1287	1.32 (1.14 to 1.52)***		1.01 (0.82 to 1.23)
High level of distress	2000	1.22 (1.07 to 1.39)**		1.22 (1.03 to 1.44)*
No feelings of control	1337	1.24 (1.08 to 1.43)**		1.09 (0.90 to 1.31)
Referred/seen by other team				
Never referred to crisis team	2459	Reference group		
Ever referred to the crisis team	2518	1.27 (1.12 to 1.44)***		1.34 (1.14 to 1.56)***
Never seen at A&E‡	2918	Reference group		
Ever seen at A&E‡	2059	0.96 (0.84 to 1.09)		0.93 (0.80 to 1.08)
Never referred to assertive outreach	4636	Reference group		
Ever referred to assertive outreach	341	0.67 (0.51 to 0.89)**		0.81 (0.60 to 1.08)
Forensic history				
No forensic history reported	4326	Reference group		
Forensic history reported	651	0.80 (0.66 to 0.98)**		0.86 (0.69 to 1.06)
Context of first CBT session				
First CBT as outpatient	3493	Reference group		
First CBT as inpatient	1484	0.35 (0.29 to 0.41) ***		0.35 (0.29 to 0.42) ***

Step 1: adjusted for ethnicity+gender+age+IMD decile+marital status+diagnosis: psychosis/bipolar.

Step 2: adjusted for ethnicity+gender+age+IMD decile+marital status+diagnosis: psychosis/bipolar+substance use diagnosis+inpatient admittance+treated under the MHA+structured risk assessment items (entered separately)+referred to crisis team+treated at A&E+referred to assertive outreach+forensic history+first CBT as inpatient.

*P<0.05; **p<0.01; ***p<0.001.

†For brevity, reference groups are omitted. Reference groups are a non-affirmative response to the item. The n for the reference group is the number of people included in the analysis (N=4977) – the number of people with an affirmative response.

‡Seen at A&E due to mental health emergency.

1, least deprived; 10, most deprived; CBT, cognitive–behavioural therapy; IMD, Index of Multiple Deprivation.

### Ethnicity and reported receipt of CBT as an inpatient

Analyses were restricted to participants who had been an inpatient (n=9417) and associations investigated with receipt or not of CBT in this setting. Unadjusted and adjusted associations are displayed in table 4. The adjusted model demonstrated that the Black African group (OR 0.76, 95% CI 0.65 to 0.89, p=0.001) and the Black Caribbean group (OR 0.83, 95% CI 0.73 to 0.94, p=0.003) were significantly less likely to have received CBT than the white British group.

**Table 4:** Crude and adjusted associations from logistic regression models for at least one recorded session of CBT as an inpatient

Variable	N	OR (95% CI)		
		Crude associations	Step 1	Step 2
Ethnicity				
White British	4000	Reference group		
Irish	232	0.95 (0.69 to 1.32)	1.02 (0.73 to 1.41)	0.99 (0.71 to 1.39)
Black African	1734	0.82 (0.71 to 0.95)**	0.80 (0.69 to 0.93)**	0.76 (0.65 to 0.89)**
Black Caribbean	3132	0.93 (0.83 to 1.05)	0.91 (0.80 to 1.02)	0.83 (0.73 to 0.94)**
South Asian	319	0.82 (0.62 to 1.10)	0.83 (0.62 to 1.12)	0.86 (0.64 to 1.16)
Gender				
Female	4390	Reference group		
Male	5027	0.93 (0.84 to 1.03)	0.89 (0.80 to 0.99)	0.87 (0.79 to 0.97)
Age (years)		0.99 (0.99 to 1.00)***	0.99 (0.99 to 1.00)***	0.99 (0.99 to 0.99)***
Area-level deprivation				
IMD decile (per 10th)		0.97 (0.95 to 0.99)**	0.97 (0.96 to 0.99)**	0.97 (0.95 to 0.99)**
Marital status				
In relationship	1234	Reference group		
Single	8183	1.24 (1.06 to 1.45)**	1.19 (1.02 to 1.40)*	1.08 (0.91 to 1.27)
Diagnosis				
Psychosis	7114	Reference group		
Bipolar affective disorder	2303	0.97 (0.86 to 1.09)	0.94 (0.83 to 1.06)	1.02 (0.90 to 1.16)
Comorbid substance misuse				
No previous substance misuse diagnosis	7456	Reference group		
Lifetime comorbid substance misuse diagnosis	1961	1.05 (0.93 to 1.19)		0.88 (0.77 to 1.00)
Treatment under Mental Health Act (MHA)				
No treatment under MHA	2506	Reference group		
Ever treated under MHA	6911	1.56 (1.38 to 1.76)***		1.39 (1.21 to 1.59)***
Structured risk assessment items				
History of violence	4914	1.56 (1.41 to 1.73)***		1.13 (1.00 to 1.28)*
Difficulty managing physical health	2720	1.59 (1.44 to 1.77)***		1.34 (1.19 to 1.51)***
History of non-adherence	5161	1.66 (1.50 to 1.84)***		1.24 (1.09 to 1.41)**
History of suicide attempt	2879	1.61 (1.46 to 1.79)***		1.17 (1.00 to 1.35)*
Lethal means used in suicide attempt	1612	1.56 (1.38 to 1.77)***		1.02 (0.86 to 1.20)
Plans to end life	754	1.66 (1.41 to 1.96)***		1.09 (0.89 to 1.32)
Suicidal ideation	1684	1.66 (1.47 to 1.87)***		1.14 (0.97 to 1.33)
Feelings of hopelessness	2218	1.66 (1.48 to 1.85)***		1.08 (0.93 to 1.25)
High level of distress	3747	1.82 (1.65 to 2.02)***		1.37 (1.22 to 1.54)***
No feelings of control	2370	1.68 (1.51 to 1.87) *		1.08 (0.94 to 1.24)
Referred/seen by other team				
Never referred to crisis team	4217	Reference group		
Ever referred to the crisis team	5200	1.08 (0.97 to 1.19)		0.90 (0.80 to 1.00)*
Never seen at A&E	4981	Reference group		
Ever seen at A&E	4436	1.22 (1.10 to 1.34) ***		1.11 (1.00 to 1.23)
Never referred to assertive outreach	8633	Reference group		
Ever referred to assertive outreach	784	1.45 (1.23 to 1.71) ***		1.18 (0.99 to 1.41)
Forensic history				
No forensic history reported	7936	Reference group		
Forensic history reported	1481	1.11 (0.97 to 1.27)		1.02 (0.89 to 1.18)

Step 1: adjusted for ethnicity+gender+age+IMD decile+marital status+diagnosis: psychosis/bipolar.

Step 2: adjusted for ethnicity+gender+age+IMD decile+marital status+diagnosis: psychosis/bipolar+substance use diagnosis+treated under the MHA+structured risk assessment items (entered separately)+referred to crisis team+treated at A&E+referred to assertive outreach+forensic history.

*P<0.05; **p<0.01; ***p<0.001.

†For brevity, reference groups are omitted. Reference groups are a non-affirmative response to the item. The n for the reference group is the number of people included in the analysis (N=9417) – the number of people with an affirmative response.

‡Seen at A&E due to mental health emergency.

1, least deprived; 10, most deprived; CBT, cognitive–behavioural therapy; IMD, Index of Multiple Deprivation.

### Supplementary analyses

Analyses using the less stringent definition of a course of CBT (≥5 sessions) indicated the Black African group were significantly less likely to receive this in comparison to the white British group (OR 0.76, 95% CI 0.63 to 0.91, p=0.003) (see online supplemental table 3). Analyses of CBT sessions received only as an outpatient also indicated that the Black African group (OR 0.75, 95% CI 0.67 to 0.84, p<0.001) were significantly less likely to receive this than the white British group (see online supplemental table 4).

### Post hoc sensitivity analysis

#### Recording of clinical risk

The crude estimates indicated that several variables indicative of higher clinical risk and severity were associated with increased odds of having a (single) documented session of CBT (table 2). We considered that this may be because CBT is better recorded (rather than more likely to be delivered) for those at an increased risk (eg, of harming themselves, suicide and harming others) and proposed that, if defensive practice resulted in better note keeping, this would be most likely evident in the structured fields. Consequently, as a supplementary sensitivity analysis, using the entire sample (n=20 010), models assessing reported receipt of CBT were rerun omitting entries identified in the structured fields, (ie, just using data derived from free text). However, this analysis continued to indicate an association between Black African group membership and significantly lower odds of receiving CBT than white British group membership (OR 0.76, 95% CI 0.63 to 0.92, p=0.004). Adjusted and unadjusted ORs are presented in online supplemental table 5.

#### Influence of time

Additional analyses were conducted to assess if changes over time affected referral practices for psychological treatments. To this end, a variable was created indicating participants who had received a diagnosis of psychosis or bipolar affective disorder after the midpoint of the data collection window (ie, after 16 April 2012). Models considering ethnicity and reported receipt of CBT were rerun including the variable indicating the date at which diagnosis was given. This analysis also indicated that the Black African group were significantly less likely to receive CBT than the white British group (OR 0.72, 95% CI 0.65 to 0.81, p<0.001), suggesting that this finding was not influenced by the date diagnosis was given (see online supplemental table 6). In the fully adjusted model, receiving a diagnosis of psychosis or bipolar affective disorder after the midpoint of the data collection window was associated with decreased odds of a documented session of CBT (OR 0.77, 95% CI 0.71 to 0.83, p<0.001). Furthermore, analysis was conducted to assess if there was an interaction between time and ethnicity; however, a likelihood ratio test indicated that fitting this interaction term did not significantly improve the model: χ^2^ (4)=5.25, p*=*0.26.

## Discussion

### Statement of principal findings

This investigation found that after adjustment for numerous indicators of risk and severity, in comparison with white British counterparts, Black African people with bipolar disorder or psychosis were less likely to have a documented session of CBT, a finding that was robust to a number of sensitivity analyses. After adjustment for indicators of risk and symptom severity in comparison with white British people, Black Caribbean people were also less likely to receive CBT as inpatients and were less likely to receive the minimum 16 sessions recommended by national guidelines. This study also found that regardless of ethnicity, people who had their first documented session of CBT as an inpatient were less likely to receive a minimum of 16 sessions of CBT (and a similar effect was also noted in supplementary analyses of a minimum five documented sessions and documented receipt of CBT as an outpatient). In addition, regardless of ethnicity indicators of higher risk and severity of symptoms were typically associated with higher odds of receiving CBT; however, these associations between risk status and receipt of CBT were less consistent in analyses of a minimum 16 documented sessions.

### Strengths and limitations of the study

To our knowledge, this study has used the largest sample to date to assess ethnic differences in access to CBT for people with psychosis or bipolar affective disorder. This study used a case register from a large mental healthcare provider serving a socially and ethnically diverse geographic catchment. Furthermore, the data were sourced from the full electronic health record, using a case register with near-complete coverage of people receiving mental healthcare for these diagnoses. The study used a tool to extract information about CBT from structured fields and free text, an approach that has been shown to have high positive predictive value and sensitivity values in previous work.^19^ Consequently, this study likely provides a highly accurate picture of access to CBT delivered by mental health services within the catchment. Of note, despite having recognised high incidence rates of psychosis,^38^ the catchment is not dissimilar to other parts of London and UK urban areas on several sociodemographic metrics^36 37^; the results of this investigation may generalise to other urban and semiurban multicultural areas in England, a notion that is supported by ethnic disparity in access to therapy indicated in nationally representative data.^2^ By accessing a large data set of complete clinical records, we were able to contribute novel findings relating to the impact of risk and pathways on engagement with CBT. However, one limitation of this investigation is that it was not possible to extract information from the BRC Case Register about other psychological therapies, some of which are recommended by national guidelines and delivered routinely within the services analysed (eg, family intervention^13^). It is possible therefore that disparity in access to CBT may be accounted for by ethnic differences in preference for therapy type, although this has not been suggested to be the case in other studies of national data from the UK.^2^ Another limitation is that although this study likely displays an accurate picture of service users who *received* CBT, it was not possible to derive information about the *offer* of CBT. If service users are not accepting CBT or completing a course, or alternatively service providers are not offering or delivering a course of CBT, it is important to understand why. This could be explored in future research.

An additional limitation of this study is we did not extract information regarding the length of inpatient stay. The consequence of this is we do not know the impact of length of stay on the likelihood that someone receives CBT. It is feasible that people who have very short inpatient stays are less likely to receive CBT than those who spend longer in that environment.

### Strengths of this study in relation to other research

Our findings replicate those observed for unselected community residents from a nationally representative sample, namely less equitable access to CBT for ethnic minority groups.^2^ Previous investigations that have explored ethnic disparities in access/engagement with CBT in samples with psychosis have not differentiated between Black African and Black Caribbean people,^2 19 23 24^ despite the two groups typically having different migratory histories and different factors influencing pathways into treatment for psychosis.^50^ The current investigation was able to define more specific ethnic categories providing a more nuanced understanding of ethnicity and access to CBT.

### Comparisons with previous research

Previous research has highlighted that more positive symptoms in psychosis increase referrals for CBT.^17^ Our study extended this finding by highlighting that numerous indicators of higher symptom severity and risk increase the propensity to receive a minimum of one session of CBT. However, despite controlling for these variables, this study found persistent disparities by ethnicity in receipt of CBT (ie, a minimum of one documented session). The relationship between risk and CBT engagement (ie, documented receipt of a minimum of 16 sessions) appeared less consistent. Several of the risk indicators that increased the odds of receiving one documented session of CBT were not significantly associated either way with receipt of a minimum of 16 sessions. This may suggest a more complex relationship between risk and CBT engagement. The positive association between recorded level of clinical risk and receipt of CBT is in contrast to research suggesting that inequalities between ethnic groups in mental health treatment could be caused by differences in symptom severity.^7 8^ Despite risk indicators (typically) increasing access to CBT and previous investigations suggesting that Black women are most likely to self-harm^51^; the current investigation does not indicate that ethnic disparities in the receipt of CBT is as a consequence of ethnic differences in risk or symptom profile.

First, access of CBT as an inpatient was associated with lower odds of receiving further CBT sessions. There are numerous potential explanations. For example, coercive practice in inpatient settings has been well documented, and this may potentially impact on subsequent engagement.^52^ Alternatively, our finding may be related to differences in recovery styles.^53^ An avoidant recovery style (referred to as sealing over) has been linked to poorer engagement with services,^54^ and it is possible that some people are receptive to psychological therapy at the point of crisis (ie, during inpatient stay), but once there is a diminution of symptoms, they ‘seal over’ that reduces engagement.

### Implications of this research and suggestions for future research

Our study suggests that, within clinical settings, further work is needed to ensure there is parity in access to CBT. In practice, this might include ensuring that CBT is systematically offered to groups who are less likely to receive treatment. It is also feasible that further work is needed to ensure that CBT is more acceptable to Black groups that might be achieved by culturally adapting interventions.^55^ Nonetheless, more research is required to explore the reasons underpinning ethnicity difference in access to CBT, whether ethnic differences in receipt of CBT extend to the offer of CBT, and the impact clinical risk has on engagement with CBT. Moreover, further research is necessary to explore the impact of pathways into care or psychological treatment and its role in subsequent engagement.

## Supplementary Material

Reviewer comments

Author's manuscript

## References

[1] Mann F, Fisher HL, Major B, et al Ethnic variations in compulsory detention and hospital admission for psychosis across four UK early intervention services. BMC Psychiatry 2014;14:256. 10.1186/s12888-014-0256-1 25214411 PMC4173060

[2] Das-Munshi J, Bhugra D, Crawford MJ Ethnic minority inequalities in access to treatments for schizophrenia and schizoaffective disorders: findings from a nationally representative cross-sectional study. BMC Med 2018;16:55. 10.1186/s12916-018-1035-5 29669549 PMC5904997

[3] Evans R, Makala J, Humphreys M, et al Supervised community treatment in Birmingham and Solihull: first 6 months. Psychiatrist 2010;34:330–3. 10.1192/pb.bp.109.027482

[4] Horvitz-Lennon M, McGuire TG, Alegria M, et al Racial and ethnic disparities in the treatment of a Medicaid population with schizophrenia. Health Serv Res 2009;44:2106–22. 10.1111/j.1475-6773.2009.01041.x 19780855 PMC2796317

[5] Daumit GL, Crum RM, Guallar E, et al Outpatient prescriptions for atypical antipsychotics for African Americans, Hispanics, and whites in the United States. Arch Gen Psychiatry 2003;60:121–8. 10.1001/archpsyc.60.2.121 12578429

[6] Vinkers DJ, de Beurs E, Barendregt M, et al Pre-trial psychiatric evaluations and ethnicity in the Netherlands. Int J Law Psychiatry 2010;33:192–6. 10.1016/j.ijlp.2010.03.010 20403639

[7] Gajwani R, Parsons H, Birchwood M, et al Ethnicity and detention: are black and minority ethnic (BME) groups disproportionately detained under the mental health act 2007? Soc Psychiatry Psychiatr Epidemiol 2016;51:703–11. 10.1007/s00127-016-1181-z 26886264 PMC4846695

[8] Singh SP, Burns T, Tyrer P, et al Ethnicity as a predictor of detention under the mental health act. Psychol Med 2014;44:997–1004. 10.1017/S003329171300086X 23795603

[9] Al-Sharifi A, Krynicki CR, Upthegrove R Self-harm and ethnicity: a systematic review. Int J Soc Psychiatry 2015;61:600–12. 10.1177/0020764015573085 25783961

[10] Veling W, Selten J-P, Mackenbach JP, et al Symptoms at first contact for psychotic disorder: comparison between native Dutch and ethnic minorities. Schizophr Res 2007;95:30–8. 10.1016/j.schres.2007.06.024 17669627

[11] Jivraj S, Khan O Ethnicity and deprivation in England: how likely are ethnic minorities to live in deprived neighbourhoods? centre on dynamics of ethnicity briefing paper, 2013 Available: http://hummedia.manchester.ac.uk/institutes/code/briefingsupdated/ethnicity-and-deprivation-in-england-how-likely-are-ethnic-minorities-to-live-in-deprived-neighbourhoods%20(1).pdf [Accessed 1 Feb 2018].

[12] Shih RA, Miles JNV, Tucker JS, et al Racial/ethnic differences in adolescent substance use: mediation by individual, family, and school factors. J Stud Alcohol Drugs 2010;71:640–51. 10.15288/jsad.2010.71.640 20731969 PMC2930496

[13] National Institute for Health and Care Excellence Psychosis and schizophrenia in adults: the NICE guideline on treatment and management (CG178. London: NICE, 2014.

[14] Royal College of Psychiatrists Report of the second round of the National audit of schizophrenia (Nas2) 2014. London: Royal College of Psychiatrists Centre for Quality Improvement, 2014.

[15] Hazell CM, Strauss C, Cavanagh K, et al Barriers to disseminating brief CBT for voices from a lived experience and clinician perspective. PLoS One 2017;12:e0178715. 10.1371/journal.pone.0178715 28575094 PMC5456317

[16] Prytys M, Garety PA, Jolley S, et al Implementing the NICE guideline for schizophrenia recommendations for psychological therapies: a qualitative analysis of the attitudes of CMHT staff. Clin Psychol Psychother 2011;18:48–59. 10.1002/cpp.691 21110400

[17] Fanning F, Foley S, Lawlor E, et al Group cognitive behavioural therapy for first episode psychosis: who's referred, who attends and who completes it? Early Interv Psychiatry 2012;6:432–41. 10.1111/j.1751-7893.2011.00333.x 22240156

[18] Memon A, Taylor K, Mohebati LM, et al Perceived barriers to accessing mental health services among black and minority ethnic (BME) communities: a qualitative study in Southeast England. BMJ Open 2016;6:e012337. 10.1136/bmjopen-2016-012337 PMC5128839 27852712

[19] Colling C, Evans L, Broadbent M, et al Identification of the delivery of cognitive behavioural therapy for psychosis (CBTp) using a cross-sectional sample from electronic health records and open-text information in a large UK-based mental health case register. BMJ Open 2017;7:e015297. 10.1136/bmjopen-2016-015297 PMC5734297 28716789

[20] McKenzie K, Samele C, Van Horn E, et al Comparison of the outcome and treatment of psychosis in people of Caribbean origin living in the UK and British whites. Report from the UK700 trial. Br J Psychiatry 2001;178:160–5. 10.1192/bjp.178.2.160 11157430

[21] McKenzie K, van Os J, Fahy T, et al Psychosis with good prognosis in Afro-Caribbean people now living in the United Kingdom. BMJ 1995;311:1325–7. 10.1136/bmj.311.7016.1325 7496280 PMC2551241

[22] Walkup J, Wei W, Sambamoorthi U, et al Provision of psychotherapy for a statewide population of Medicaid beneficiaries with schizophrenia. Psychol Serv 2006;3:227–38. 10.1037/1541-1559.3.4.227

[23] Jolley S, Garety P, Peters E, et al Opportunities and challenges in improving access to psychological therapies for people with severe mental illness (IAPT-SMI): evaluating the first operational year of the South London and Maudsley (SLAM) demonstration site for psychosis. Behav Res Ther 2015;64:24–30. 10.1016/j.brat.2014.11.006 25499927

[24] Johns L, Jolley S, Garety P, et al Improving access to psychological therapies for people with severe mental illness (IAPT-SMI): lessons from the South London and Maudsley psychosis demonstration site. Behav Res Ther 2019;116:104–10. 10.1016/j.brat.2019.03.002 30877877

[25] Sweeney A, Fahmy S, Nolan F, et al The relationship between therapeutic alliance and service user satisfaction in mental health inpatient wards and crisis house alternatives: a cross-sectional study. PLoS One 2014;9:e100153. 10.1371/journal.pone.0100153 25010773 PMC4091866

[26] Holdsworth E, Bowen E, Brown S, et al The development of a program engagement theory for group offending behavior programs. Int J Offender Ther Comp Criminol 2017;61:1479–99. 10.1177/0306624X15624177 26769679

[27] Barrett MS, Chua W-J, Crits-Christoph P, et al Early withdrawal from mental health treatment: implications for psychotherapy practice. Psychotherapy 2008;45:247–67. 10.1037/0033-3204.45.2.247 19838318 PMC2762228

[28] Mantovani N, Pizzolati M, Edge D Exploring the relationship between stigma and help-seeking for mental illness in African-descended faith communities in the UK. Health Expect 2017;20:373–84. 10.1111/hex.12464 27124178 PMC5433535

[29] Keating F, Robertson D, McCulloch A, et al Breaking the circles of fear: a review of the relationship between mental health services and African and Caribbean communities. London: The Sainsbury Centre for Mental Health, 2002.

[30] Rathod S, Kingdon D, Phiri P, et al Developing culturally sensitive cognitive behaviour therapy for psychosis for ethnic minority patients by exploration and incorporation of service users' and health professionals' views and opinions. Behav Cogn Psychother 2010;38:511–33. 10.1017/S1352465810000378 20630118

[31] Chen J, Rizzo J Racial and ethnic disparities in use of psychotherapy: evidence from U.S. national survey data. Psychiatric Serv 2010;61:364–72. 10.1176/ps.2010.61.4.364 20360275

[32] Islam Z, Rabiee F, Singh SP Black and minority ethnic groups’ perception and experience of early intervention in psychosis services in the United Kingdom. J Cross Cult Psychol 2015;46:737–53. 10.1177/0022022115575737

[33] Blofeld J Independent inquiry into the death of David Bennett. Cambridgshire, Norfolk, Suffolk and Cambridgeshire: Strategic Health Authority, 2003.

[34] Royal College of Psychiatrists Racism and mental health: position statement 01/18. London: Royal College of Psychiatrists, 2018.

[35] Lally J, Wong YL, Shetty H, et al Acute hospital service utilization by inpatients in psychiatric hospitals. Gen Hosp Psychiatry 2015;37:577–80. 10.1016/j.genhosppsych.2015.07.006 26319481

[36] Perera G, Broadbent M, Callard F, et al Cohort profile of the South London and Maudsley NHS Foundation trust biomedical research centre (SLAM BRC) case register: current status and recent enhancement of an electronic mental health Record-derived data resource. BMJ Open 2016;6:e008721 10.1136/bmjopen-2015-008721 PMC4785292 26932138

[37] Stewart R, Soremekun M, Perera G, et al The South London and Maudsley NHS Foundation trust biomedical research centre (SLAM BRC) case register: development and descriptive data. BMC Psychiatry 2009;9:51. 10.1186/1471-244X-9-51 19674459 PMC2736946

[38] Jongsma HE, Gayer-Anderson C, Lasalvia A, et al Treated incidence of psychotic disorders in the multinational eu-gei study. JAMA Psychiatry 2018;75:36–46. 10.1001/jamapsychiatry.2017.3554 29214289 PMC5833538

[39] Fernandes AC, Cloete D, Broadbent M, et al Development and evaluation of a de-identification procedure for a case register Sourced from mental health electronic records. BMC Psychiatry 2013;13:71. 23842533 10.1186/1472-6947-13-71 PMC3751474

[40] Das-Munshi J, Chang C-K, Dutta R, et al Ethnicity and excess mortality in severe mental illness: a cohort study. Lancet Psychiatry 2017;4:389–99. 10.1016/S2215-0366(17)30097-4 28330589 PMC5406616

[41] Office for National Statistics Measuring equality: a guide for the collection and classification of ethnic group, National identity and religion data in the UK. (n.d.). Available: www.ons.gov.uk/methodology/classificationsandstandards/measuringequality/ethnicgroupnationalidentityandreligion [Accessed 4 Oct 2019].

[42] Das-Munshi J, Ashworth M, Gaughran F, et al Ethnicity and cardiovascular health inequalities in people with severe mental illnesses: protocol for the E-CHASM study. Soc Psychiatry Psychiatr Epidemiol 2016;51:627–38. 10.1007/s00127-016-1185-8 26846127 PMC4823321

[43] Aggarwal C, Zhai C Mining text data. New York: Springer, 2012.

[44] WHO International statistical classification of diseases and related health problems 10th version. Geneva: World Health Organisation, 2010 http://apps.who.int/classifications/icd10/browse/2016/en

[45] Jackson RG, Patel R, Jayatilleke N, et al Natural language processing to extract symptoms of severe mental illness from clinical text: the clinical record interactive search comprehensive data extraction (CRIS-CODE) project. BMJ Open 2017;7:e012012. 10.1136/bmjopen-2016-012012 PMC5253558 28096249

[46] Department for Communities and Local Government The English index of multiple deprivation (IMD) 2015 – guidance. London: Home Office, 2015 assets.publishing.service.gov.uk/government/uploads/system/uploads/attachment_data/file/464430/English_Index_of_Multiple_Deprivation_2015_-_Guidance.pdf

[47] Woodhead C, Ashworth M, Broadbent M, et al Cardiovascular disease treatment among patients with severe mental illness: a data linkage study between primary and secondary care. Br J Gen Pract 2016;66:e374–81. 10.3399/bjgp16X685189 27114210 PMC4871302

[48] National Health Service England Implementing the early intervention in psychosis access and waiting time standard: guidance. London: National Collaborating Centre for Mental Health, National Institute for Health and Care Excellence, 2016 www.england.nhs.uk/mentalhealth/wp-content/uploads/sites/29/2016/04/eip-guidance.pdf

[49] National Institute for Health and Care Excellence Bipolar disorder: assessment and management (CG185). London: NICE, 2014. 31487127

[50] Bhui K, Ullrich S, Kallis C, et al Criminal justice pathways to psychiatric care for psychosis. Br J Psychiatry 2015;207:523–9. 10.1192/bjp.bp.114.153882 26294370 PMC4664857

[51] Cooper J, Murphy E, Webb R, et al Ethnic differences in selfharm, rates, characteristics and service provision: three city cohort study. Br J Psychiatry 2010;2010:212–8. 10.1192/bjp.bp.109.072637 20807966

[52] Department of Health and Social Care Modernising the mental health act: increasing choice, reducing compulsion. London: Home Office, 2018 assets.publishing.service.gov.uk/government/uploads/system/uploads/attachment_data/file/762206/MHA_reviewFINAL.pdf

[53] Drayton M, Birchwood M, Trower P Early attachment experience and recovery from psychosis. Br J Clin Psychol 1998;37:269–84. 10.1111/j.2044-8260.1998.tb01385.x 9784883

[54] Tait L, Birchwood M, Trower P Adapting to the challenge of psychosis: personal resilience and the use of sealing-over (avoidant) coping strategies. Br J Psychiatry 2004;185:410–5. 10.1192/bjp.185.5.410 15516550

[55] Rathod S, Phiri P, Harris S, et al Cognitive behaviour therapy for psychosis can be adapted for minority ethnic groups: a randomised controlled trial. Schizophr Res 2013;143:319–26. 10.1016/j.schres.2012.11.007 23231878

