# Supplementary material for: Ethnicity and impact on the receipt of cognitive–behavioural therapy in people with psychosis or bipolar disorder: an English cohort study
Source: BMJ Open. 2020 Dec 15;10(12):e034913. doi: 10.1136/bmjopen-2019-034913 (PMC7745324; doi:10.1136/bmjopen-2019-034913)
Supplement: Supplementary data [file bmjopen-2019-034913supp001.pdf]

Supplementary Data Contents

Supplementary Data 1

Supplementary Data 1 contains further information regarding treatment, risk, and severity including items from a structured risk assessment. The table also contains tests of difference between the different ethnic groups.

Supplementary Data 2

Supplementary Data 2 contains information about CBT treatment received. The table displays the relevant proportions for each ethnic group and tests of difference.

Supplementary Data 3

Supplementary Data 3 contains crude estimates and adjusted multivariable logistic regression models regarding ethnicity and having a minimum of 5 sessions of CBT.

Supplementary Data 4

Supplementary Data 4 contains crude estimates and adjusted multivariable logistic regression models regarding ethnicity and reported receipt of CBT as an outpatient.

Supplementary Data 5

Supplementary Data 5 contains crude estimates and adjusted multivariable logistic regression models regarding ethnicity and reported receipt of CBT in the unstructured clinical notes (i.e. just using data derived from free text not structured fields).

.

Supplementary Data 6

Supplementary Data 6 contains crude estimates and adjusted multivariable logistic regression models regarding ethnicity and reported receipt of CBT which are adjusted for the effect of time.

BMJ Publishing Group Limited (BMJ) disclaims all liability and responsibility arising from any reliance placed on this supplemental material which has been supplied by the author(s)

Supplementary Data 1

Table 1
Information about clients' treatment and risk assessment

		White British		Irish		African		Caribbean		South Asian		Total			
		N	%	N	%	N	%	n	%	n	%	N	%	Chi^2*	*p*-value
	Ever treated under Mental Health Act	2575	24.8	155	27.2	1492	53.0	2648	48.3	236	31.5	7106	35.5	1300.0	<*.001*
	Inpatient admission ever	4000	38.5	232	40.7	1734	61.6	3132	57.1	319	42.6	9417	47.1	783.0	<*.001*
	Forensic History#	836	8.0	44	7.7	260	9.2	700	12.8	33	4.4	1873	9.4	119.9	<*.001*
Structured risk assessment items	History of violence	2324	22.4	140	24.6	1086	38.6	2491	45.4	175	23.4	6216	31.1	1000.0	<*.001*
	Difficulty managing physical health	1645	15.8	98	17.2	513	18.2	1240	22.6	126	16.8	3622	18.1	113.0	<*.001*
	History of non-adherence	2320	22.3	150	26.3	1177	41.8	2579	47.1	199	26.6	6425	32.1	1200.0	<*.001*
	History of suicide attempt	2062	19.8	119	20.9	445	15.8	1045	19.1	87	11.6	3758	18.8	51.2	<*.001*
	Lethal means used in suicide attempt	1157	56.1	67	56.3	230	51.7	531	50.8	41	47.1	2026	53.9	44.1	<*.001*

Current plans to end life	524	5.0	25	4.4	109	3.9	186	3.4	19	2.5	863	4.3	31.7	*<.001*
Current suicidal ideation	1126	10.8	73	12.8	256	9.1	526	9.6	60	8.0	2041	10.2	18.7	*<.01*
Hopelessness	1481	14.3	93	16.3	398	14.1	797	14.5	81	10.8	2850	14.2	9.6	*.047*
High levels of distress	1986	19.1	129	22.6	808	28.7	1595	29.1	148	19.8	4666	23.3	256.3	*<.001*
No perception of control	1404	13.5	86	15.1	442	15.7	950	17.3	90	12.0	2972	14.9	47.8	*<.001*
Crisis team ever	2642	25.4	145	25.4	1241	44.1	2209	40.3	269	35.9	6506	32.5	577.8	*<.001*
Assertive outreach ever	496	4.8	10	1.8	149	5.3	348	6.3	30	4.0	1033	5.2	34.7	*<.001*
A&E ever~	3172	30.5	191	33.5	1125	39.9	1879	34.3	254	33.9	6621	33.1	94.4	*<.001*

Forensic history noted in the clinical records includes self-reported and reports by professionals from different organisations; ~ Seen at A & E due to mental health; * All degrees of freedom = 4

Supplementary Data 2

Table 2

Information about CBT and the relevant proportions by ethnicity

	White British		Irish		African		Caribbean		South Asian		Total		Kruskal-Wallis *H*, Chi^2 *	*p*-value
	N	%	N	%	n	%	n	%	n	%	N	%		
CBT ever	2541	24.5	139	24.4	722	25.6	1613	29.4	182	24.3	5197	26.0	48.6	<.001
CBT on-going	160	6.3	9	6.5	66	9.1	141	8.7	10	5.5	386	7.4	24	<.001
Sessions: median (IQR)#	6 (14)		8 (15)		4 (12)		5 (11)		6 (14)		5 (13)		37.2	<.001
CBT inpatient ever	833	8.0	47	8.3	318	11.3	644	11.8	57	7.6	1899	9.5	47.1	<.001
Inpatient sessions: median (IQR) ##	2 (5)		2 (3)		2 (2)		2 (3)		1 (3)		2 (3)		22.4	<.001
CBT outpatient ever	2011	19.4	113	19.8	514	18.3	1197	21.8	147	19.6	3982	19.9	14.2	<.01
Outpatient sessions: median (IQR) ###	8 (16)		8 (16)		6 (15)		6 (14)		8 (16)		7 (15)		38.1	<.001
% of sessions Face to Face	96.5		97.8		97.0		96.6		98.3		96.7			

% of sessions one to one	94.7		91.7		92.4		93.1		96.5		93.9			
1st CBT as inpatient##	670	26.4	37	26.6	262	36.3	537	33.3	51	28.0	1557	30.0	68.2	<.001
>= 5 sessions~	1477	58.9	82	59.4	344	48.9	818	52.3	101	56.1	2822	55.4	31.4	<.001
>= 16 sessions~	686	27.9	39	28.7	158	23.2	352	23.1	49	27.5	1284	25.8	14.9	<.01

* Kruskal-Wallis H non parametric test for ranked data used to determine the Chi^2 value , all degrees of freedom = 4; # Including only the people who had received CBT; ## Including only people who have received CBT as an inpatient; ### Including only people who have received CBT as an outpatient

~ Only includes people who had ever received CBT and people who had not received CBT within 6 weeks of data extraction if they did not meet the criteria (i.e. people with <5 or <16 sessions who were currently receiving CBT)

Supplementary Data 3

Table 3

Crude and adjusted associations from logistic regression models for at least five recorded sessions of CBT (inpatient or outpatient)

Variable	N	Odds Ratio (95% Confidence Interval)		
		Crude Associations	Step 1	Step 2
**Ethnicity**				
White British	2509	Reference group		
Irish	139	1.01 (0.71-1.42)	1.02 (0.72-1.44)	1.02 (0.71-1.47)
Black African	704	0.67 (0.56-0.79) ***	0.68 (0.57-0.81) ***	0.76 (0.63-0.91) **
Black Caribbean	1565	0.77 (0.67-0.89) ***	0.79 (0.69-0.90) ***	0.88 (0.76-1.01)
South Asian	180	0.89 (0.66-1.21) ***	0.88 (0.64-1.19)	0.91 (0.66-1.25)
**Gender**				
Female	2543	Reference Group		
Male	2554	0.97 (0.87-1.09) ***	0.99 (0.88-1.11)	1.09 (0.96-1.23)
**Age (years)**		1.00 (1.00-1.00)	1.00 (1.00-1.00)	1.00 (1.00-1.00)
**Area level deprivation**				
IMD decile (per tenth)		0.97 (0.96-0.99) *	0.99 (0.97-1.01)	0.98 (0.96-1.00)*
**Marital Status**				
In relationship	654	Reference Group		
Single	4443	0.84 (0.71-0.99) *	0.87 (0.73-1.03)	0.95 (0.79-1.14)
**Diagnosis**				
Psychosis	3734	Reference Group		
Bipolar affective disorder	1363	1.14 (1.01-1.29) *	1.04 (0.92-1.19)	1.00 (0.87-1.15)
**Comorbid substance misuse**				
No previous substance misuse diagnosis	4191	Reference group		
Lifetime comorbid substance misuse diagnosis	906	0.78 (0.68-0.90) **		0.79 (0.68-0.93) **
**Admission**				
No previous admission	1650	Reference Group		
Inpatient admission ever	3447	0.64 (0.57-0.72) ***		1.05 (0.87-1.28)
**Treatment under Mental Health Act**				
No treatment under Mental Health Act	2473	Reference Group		
Ever treated under Mental Health Act	2624	0.63 (0.56-0.70) ***		0.91 (0.76-1.08)**
**Structured risk assessment items#**				
History of Violence	2288	0.71 (0.64-0.80) ***		0.88 (0.76-1.02)
Difficulty managing physical health	1257	0.81 (0.71-0.92) **		0.96 (0.83-1.12)
History of Non-adherence	2443	0.73 (0.66-0.82) ***		0.90 (0.77-1.05)

History of Suicide Attempt	1628	1.21 (1.08-1.37) **	1.23 (1.03-1.72) *
Lethal means used in suicide attempt	904	1.20 (1.04-1.39) *	0.98 (0.80-1.21)
Plans to end life	396	1.43 (1.16-1.77) **	1.33 (1.03-1.72) *
Suicidal ideation	991	1.30 (1.13-1.50) ***	1.13 (0.93-1.36)
Feelings of hopelessness	1318	1.20 (1.05-1.36) **	0.97 (0.81-1.16)
High level of distress	2059	1.13 (1.01-1.26) *	1.29 (1.11-1.49) **
No feelings of control	1366	1.12 (0.99-1.27)	1.06 (0.89-1.25)
**Referred/seen by other team**			
Never referred to Crisis team	2508	Reference Group	
Ever referred to the Crisis team	2589	1.13 (1.01-1.26) *	1.21 (1.05-1.38) **
Never seen at A & E~	2971	Reference Group	
Ever seen at A & E~	2126	0.96 (0.86-1.07)	1.00 (0.88-1.14)
Never referred to Assertive Outreach	4752	Reference Group	
Ever referred to Assertive Outreach	345	0.65 (0.52-0.80) ***	0.83 (0.65-1.05)
**Forensic History**			
No forensic history reported	4435	Reference Group	
Forensic History reported	662	0.72 (0.61-0.85) ***	0.81 (0.68-0.97) *
**Context of first CBT session**			
First CBT as outpatient	3584	Reference Group	
First CBT as inpatient	1513	0.33 (0.29-0.38) ***	0.34 (0.29-0.39) ***

*$p<.05$;**$p<.01$;***$p<.001$; IMD= Index of Multiple Deprivation, 1=least deprived, 10=most deprived; ~ Seen at A & E due to mental health emergency; # For brevity reference groups are omitted. Reference groups are a non-affirmative response to the item. The *n* for the reference group is the number of people included in the analysis (N=5097) – the number of people with an affirmative response.

Step 1 Adjusted for Ethnicity + Gender + Age + IMD decile + Marital Status + diagnosis: psychosis/bipolar

Step 2 Adjusted for Ethnicity + Gender + Age + IMD decile + Marital Status + diagnosis: psychosis/bipolar + Substance use diagnosis + inpatient admittance + treated under the MHA + Structured risk assessment items (entered separately) + Referred to crisis team + Treated at A & E + Referred to assertive outreach + forensic history + First CBT as inpatient

Supplementary Data 4

Table 4

Crude and adjusted associations from logistic regression models for at least one recorded session of CBT as an outpatient

Variable		Odds Ratio (95% Confidence Interval)		
	N	Crude Associations	Step 1	Step 2
**Ethnicity**				
White British	10393	Reference Group		
Irish	570	1.03 (0.84-1.27)	1.15 (0.94- 1.42)	1.10 (0.89- 1.37)
Black African	2817	0.90 (0.81-1.00)	0.83 (0.74 - 0.92) **	0.75 (0.67-0.84) ***
Black Caribbean	5481	1.11 (1.03-1.20) **	1.06 (0.98- 1.15)	0.95 (0.87- 1.04)
South Asian	749	1.00 (0.83-1.20)	0.98 (0.81-1.18)	0.97 (0.80-1.17)
**Gender**				
Female	9553	Reference Group		
Male	10457	0.85 (0.80-0.92) ***	0.82 (0.76-0.88) ***	0.85 (0.79-0.91) ***
**Age (years)**		0.99 (0.98-0.99) ***	0.98 (0.98-0.99) ***	0.99 (0.98-0.99) ***
**Area level deprivation**				
IMD decile (per tenth)		1.00 (0.99-1.01)	1.00 (0.99-1.02)	0.99 (0.98-1.00)
**Marital Status**				
In relationship	2913	Reference Group		
Single	17097	1.09 (0.98-1.20)	0.98 (0.88-1.08)	1.01 (0.91-1.13)
**Diagnosis**				
Psychosis	14497	Reference Group		
Bipolar	5513	1.09 (1.01-1.18) *	1.15 (0.94- 1.42)	1.07 (0.99-1.16)
**Comorbid substance misuse**				
No previous substance misuse diagnosis	16985	Reference group		
Lifetime comorbid substance misuse diagnosis	3025	1.15 (1.05-1.27) **		0.86 (0.78-0.95) **
**Admission**				
No previous admission	10593	Reference Group		
Inpatient Admission Ever	9417	1.69 (1.57-1.81) ***		1.13 (1.01-1.27) *
**Treatment under Mental Health Act#**				
No treatment under Mental Health Act	12904	Reference Group		
Ever treated under Mental Health Act	7106	1.40 (1.30-1.50) ***		0.75 (0.67-0.84) ***
**Structured risk assessment items#**				
History of Violence	6216	1.54 (1.43-1.65) ***		1.02 (0.93-1.13)
Difficulty managing physical health	3622	1.19 (1.09-1.29) ***		0.83 (0.75-0.92) **
History of Non-adherence	6425	1.69 (1.57-1.81) ***		1.22 (1.11-1.35) ***
History of Suicide Attempt	3758	2.25 (2.08-2.44) ***		1.38 (1.23-1.56) ***

Lethal means used in suicide attempt	2026	2.09 (1.89-2.31) ***	1.02 (0.88-1.17)
Plans to end life	863	2.01 (1.74-2.33) ***	0.79 (0.66-0.94) **
Suicidal ideation	2041	2.62 (2.38-2.89) ***	1.26 (1.11-1.44) ***
Feelings of hopelessness	2850	2.50 (2.30-2.73) ***	1.30 (1.15-1.47) ***
High level of distress	4666	2.36 (2.19-2.54) ***	1.50 (1.36-1.65) ***
No feelings of control	2972	2.33 (2.14-2.54) ***	1.21 (1.08-1.36) **
**Referred/seen by other team**			
Never referred to Crisis team	13504	Reference Group	
Ever referred to the Crisis team	6506	2.49 (2.32-2.67)***	2.02 (1.86-2.21) ***
Never seen at A & E~	13389	Reference Group	
Ever seen at A & E~	6621	1.36 (1.27-1.46)***	0.88 (0.81-0.96) **
Never referred to Assertive Outreach	18977	Reference Group	
Ever referred to Assertive Outreach	1033	0.92 (0.78-1.07)	0.71 (0.60-0.84) ***
**Forensic History**			
No forensic history reported	18137	Reference Group	
Forensic History reported	1873	1.45 (1.30-1.61) ***	1.15 (1.02-1.29) *
**Context of first CBT session**			
First CBT not as inpatient	18453	Reference Group	
First CBT as inpatient	1557	1.08 (0.95-1.23)	0.71 (0.62-0.81) ***

p<.05;p<.01;***p<.001;* IMD= Index of Multiple Deprivation, 1=least deprived, 10=most deprived; ~ Seen at A & E due to mental health emergency; # For brevity reference groups are omitted. Reference groups are a non-affirmative response to the item. The *n* for the reference group is the number of people included in the analysis (N=20010) – the number of people with an affirmative response.

Step 1 Adjusted for Ethnicity + Gender + Age + IMD decile + Marital Status + diagnosis: psychosis/bipolar

Step 2 Adjusted for Ethnicity + Gender + Age + IMD decile + Marital Status + diagnosis: psychosis/bipolar + Substance use diagnosis + inpatient admittance + treated under the MHA + Structured risk assessment items (entered separately) + Referred to crisis team + Treated at A & E + Referred to assertive outreach + forensic history + First CBT as an inpatient

Supplementary Data 5

Sensitivity Analysis

Table 5

Crude and adjusted associations from logistic regression models for at least one recorded session of CBT (inpatient or outpatient) in the unstructured clinical notes

Variable	N	Odds Ratio (95% Confidence Interval)		
		Crude Associations	Step 1	Step 2
**Ethnicity**				
White British	10393	Reference group		
Irish	570	0.88 (0.62-1.26)	1.07 (0.75-1.53)	1.04 (0.72-1.49)
Black African	2817	0.85 (0.71-1.01)	0.79 (0.66-0.95)*	0.76 (0.63-0.92) **
Black Caribbean	5481	0.96 (0.85-1.10)	0.95 (0.82-1.09)	0.90 (0.78-1.04)
South Asian	749	0.72 (0.52-1.02)	0.70 (0.50-0.99)*	0.89 (0.49-0.98)
**Gender**				
Female	9553	Reference group		
Male	10457	0.84 (0.75-0.94) **	0.83 (0.74-0.93) **	0.89 (0.79-1.00) *
**Age (years)**		0.98 (0.97-0.98) ***	0.98 (0.97-0.98) ***	0.98 (0.97-0.98) ***
**Area level deprivation**				
IMD decile (per tenth)		0.98 (0.96-1.00)*	0.99 (0.97-1.01)	0.98 (0.96-1.00)
**Marital Status**				
In relationship	2913	Reference group		
Single	17097	1.02 (0.87-1.21)	0.88 (0.74-1.04)	0.91 (0.77-1.09)
**Diagnosis**				
Psychosis	14497	Reference Group		
Bipolar affective disorder	5513	1.27 (1.12-1.43)***	1.13 (0.99-1.29)	1.17 (1.03-1.34)*
**Comorbid substance misuse**				
No previous substance misuse diagnosis	16985	Reference group		
Lifetime comorbid substance misuse diagnosis	3025	0.96 (0.82-1.13)		0.75 (0.64-0.89)**
**Admission**				
No previous admission	10593	Reference Group		
Inpatient Admission Ever	9417	1.65 (1.47-1.85) ***		1.60 (1.35-1.90) ***
**Treatment under Mental Health Act**				
No treatment under Mental Health Act	12904	Reference Group		
Ever treated under Mental Health Act	7106	1.19 (1.06-1.33) **		0.67 (0.57-0.80)***
**Structured risk assessment items#**				
History of Violence	6216	1.16 (1.03-1.31) *		0.84 (0.72-0.99)*

Difficulty managing physical health	3622	1.01 (0.87-1.17)	0.92 (0.78-1.09)
History of Non-adherence	6425	1.29 (1.15-1.45) ***	1.12 (0.95-1.31)
History of Suicide Attempt	3758	1.91 (1.69-2.17) ***	1.23 (1.02-1.50) *
Lethal means used in suicide attempt	2026	2.00 (1.72-2.32) ***	1.20 (0.97-1.50)
Plans to end life	863	2.01 (1.62-2.50) ***	0.90 (0.69-1.16)
Suicidal ideation	2041	2.42 (2.09-2.79) ***	1.43 (1.17-1.74) ***
Feelings of hopelessness	2850	1.95 (1.71-2.24) ***	1.11 (0.91-1.35)
High level of distress	4666	1.90 (1.69-2.14) ***	1.43 (1.17-1.74) ***
No feelings of control	2972	1.76 (1.54-2.02) ***	1.02 (0.85-1.23)
**Referred/seen by other team**			
Never referred to Crisis team	13504	Reference Group	
Ever referred to the Crisis team	6506	1.80 (1.61-2.02) ***	1.45 (1.26-1.66) ***
Never seen at A & E~	13389	Reference Group	
Ever seen at A & E~	6621	1.16 (1.03-1.30) *	0.76 (0.67-0.87)***
Never referred to Assertive Outreach	18977	Reference Group	
Ever referred to Assertive Outreach	1033	0.75 (0.56-1.00) *	0.61 (0.46-0.82)**
**Forensic History**			
No forensic history reported	18137	Reference Group	
Forensic History reported	1873	1.11 (0.92-1.34)	0.96 (0.78-1.17)

**p<.05; **p<.01; ***p<.001*; IMD= Index of Multiple Deprivation, 1=least deprived, 10=most deprived; ~ Seen at A & E due to mental health emergency; # For brevity reference groups are omitted. Reference groups are a non-affirmative response to the item. The *n* for the reference group is the number of people included in the analysis (N=20010) – the number of people with an affirmative response.
Step 1 Adjusted for Ethnicity + Gender + Age + IMD decile + Marital Status + diagnosis: psychosis/bipolar
Step 2 Adjusted for Ethnicity + Gender + Age + IMD decile + Marital Status + diagnosis: psychosis/bipolar + Substance use diagnosis + inpatient admittance + treated under the MHA + Structured risk assessment items (entered separately) + Referred to crisis team + Treated at A & E + Referred to assertive outreach + forensic history

Supplementary Data 6

Time analysis

Table 6

Crude and adjusted associations from logistic regression models for at least one recorded session of CBT (inpatient or outpatient) adjusting for time

Variable	N	Odds Ratio (95% Confidence Interval)		
		Crude Associations#	Step 1	Step 2
**Ethnicity**				
White British	10393	Reference group		
Irish	570	0.97 (0.80-1.19)	1.10 (0.90-1.35)	1.04 (0.84-1.28)
Black African	2817	1.06 (0.96-1.17)	0.93 (0.84-1.03)	0.72 (0.65-0.81) ***
Black Caribbean	5481	1.27 (1.18-1.36) ***	1.16 (1.08-1.25) ***	0.92 (0.85-1.00)
South Asian	749	1.00 (0.84-1.19)	0.97 (0.82-1.16)	0.93 (0.77-1.12)
**Gender**				
Female	9553	Reference group		
Male	10457	0.88 (0.83-0.94)***	0.81 (0.76-0.87) ***	0.83 (0.78-0.89) ***
**Age (years)**		0.98 (0.98-0.98) ***	0.98 (0.98-0.98) ***	0.98 (0.98-0.99) ***
**Area level deprivation**				
IMD decile (per tenth)		1.01 (1.00-1.02)	1.01 (0.99-1.02)	0.99 (0.98-1.00)
**Marital Status**				
In relationship	2913	Reference group		
Single	17097	1.22 (1.11-1.34) ***	1.56 (0.95-1.15)	1.05 (0.95-1.17)
**Diagnosis**				
Psychosis	14497	Reference Group		
Bipolar affective disorder	5513	0.96 (0.89-1.03)	0.94 (0.87-1.01)	1.01 (0.93-1.09)
**Comorbid substance misuse**				
No previous substance misuse diagnosis	16985	Reference group		
Lifetime comorbid substance misuse diagnosis	3025	1.31 (1.20-1.43) ***		0.85 (0.77-0.94)**
**Admission**				
No previous admission	10593	Reference Group		
Inpatient Admission Ever	9417	3.16 (2.96-3.38) ***		1.74 (1.56-1.93) ***
**Treatment under Mental Health Act**				
No treatment under Mental Health Act	12904	Reference Group		
Ever treated under Mental Health Act	7106	2.51 (2.35-2.68) ***		0.98 (0.88-1.09)
**Structured risk assessment items#**				
History of Violence	6216	2.26 (2.12-2.42) ***		1.08 (0.99-1.19)

Difficulty managing physical health	3622	1.68 (1.55-1.81) ***		0.96 (0.87-1.05)
History of Non-adherence	6425	2.51 (2.35-2.68) ***		1.24 (1.13-1.36) ***
History of Suicide Attempt	3758	2.79 (2.59-3.01) ***		1.35 (1.21-1.52) ***
Lethal means used in suicide attempt	2026	2.61 (2.37-2.86) ***		1.04 (0.91-1.19)
Plans to end life	863	2.64 (2.30-3.03) ***		0.82 (0.70-0.97) *
Suicidal ideation	2041	3.26 (2.97-3.58) ***		1.26 (1.11-1.43) ***
Feelings of hopelessness	2850	3.04 (2.80-3.30) ***		1.25 (1.11-1.41) ***
High level of distress	4666	3.21 (2.99-3.44) ***		1.55 (1.41-1.69) ***
No feelings of control	2972	2.98 (2.75-3.23) ***		1.20 (1.08-1.34) **
**Referred/seen by other team**				
Never referred to Crisis team	13504	Reference Group		
Ever referred to the Crisis team	6506	2.93 (2.74-3.13) ***		1.67 (1.54-1.81) ***
Never seen at A & E~	13389	Reference Group		
Ever seen at A & E~	6621	1.74 (1.63-1.86) ***		0.99 (0.91-1.06)
Never referred to Assertive Outreach	18977	Reference Group		
Ever referred to Assertive Outreach	1033	1.46 (1.27-1.66) ***		0.92 (0.80-1.06)
**Forensic History**				
No forensic history reported	18137	Reference Group		
Forensic History reported	1873	1.62 (1.46-1.79) ***		1.02 (0.91-1.14)
**Time point diagnosis given##**				
Psychosis/bipolar affective disorder diagnosis before midpoint of study period	13518	Reference category		
Psychosis/bipolar affective disorder diagnosis after midpoint of study period	6492	0.76 (0.71-0.82) ***	0.64 (0.59-0.69) ***	0.77 (0.71-0.83) ***

**p<.05; **p<.01; ***p<.001*; # including time; IMD= Index of Multiple Deprivation, 1=least deprived, 10=most deprived; ~ Seen at A & E due to mental health emergency; # For brevity reference groups are omitted. Reference groups are a non-affirmative response to the item. The *n* for the reference group is the number of people included in the analysis (N=20010) – the number of people with an affirmative response.
##midpoint of study period was 16/4/2012
Step 1 Adjusted for Ethnicity + Gender + Age + IMD decile + Marital Status + diagnosis: psychosis/bipolar + Psychosis/bipolar affective disorder diagnosis after 16/04/12
Step 2 Adjusted for Ethnicity + Gender + Age + IMD decile + Marital Status + diagnosis: psychosis/bipolar + Substance use diagnosis + inpatient admittance + treated under the MHA + Structured risk assessment items (entered separately) + Referred to crisis team + Treated at A & E + Referred to assertive outreach + forensic history + Psychosis/bipolar affective disorder diagnosis after 16/04/12
