# Supplementary material for: Ethnicity and impact on the receipt of cognitive–behavioural therapy in people with psychosis or bipolar disorder: an English cohort study
Source: BMJ Open. 2020 Dec 15;10(12):e034913. doi: 10.1136/bmjopen-2019-034913 (PMC7745324; doi:10.1136/bmjopen-2019-034913)
Supplement: Author's manuscript [file bmjopen-2019-034913.draft_revisions.pdf]

BMJ Open

BMJ Open is committed to open peer review. As part of this commitment we make the peer review history of every article we publish publicly available.

When an article is published we post the peer reviewers' comments and the authors' responses online. We also post the versions of the paper that were used during peer review. These are the versions that the peer review comments apply to.

The versions of the paper that follow are the versions that were submitted during the peer review process. They are not the versions of record or the final published versions. They should not be cited or distributed as the published version of this manuscript.

BMJ Open is an open access journal and the full, final, typeset and author-corrected version of record of the manuscript is available on our site with no access controls, subscription charges or pay-per-view fees (http://bmjopen.bmj.com).

If you have any questions on BMJ Open's open peer review process please email
info.bmjopen@bmj.com

Ethnicity and impact on the implementation of CBT in people with psychosis or bipolar disorder: A cohort study

Journal:	*BMJ Open*
Manuscript ID	bmjopen-2019-034913
Article Type:	Original research
Date Submitted by the Author:	21-Oct-2019
Complete List of Authors:	Morris, Rohan; Lancaster University, Division of Health Research; Lancashire Care NHS Foundation Trust<br>Sellwood, William; Lancaster University, Division of Health Research<br>Edge, Dawn; The University of Manchester, Division of Psychology & Mental Health, School of Health Sciences<br>Colling, Craig; King's College London, Institute of Psychiatry, Psychology and Neuroscience; South London and Maudsley NHS Foundation Trust<br>Stewart, Robert; King's College London, Institute of Psychiatry, Psychology and Neuroscience; South London and Maudsley NHS Foundation Trust<br>Cupitt, Caroline; South London and Maudsley NHS Foundation Trust<br>Das-Munshi, Jayati; King's College London, Institute of Psychiatry, Section of Epidemiology, Department of Health Service & Population Research
Keywords:	Schizophrenia & psychotic disorders < PSYCHIATRY, Depression & mood disorders < PSYCHIATRY, MENTAL HEALTH, Adult psychiatry < PSYCHIATRY

SCHOLARONE™
Manuscripts

I, the Submitting Author has the right to grant and does grant on behalf of all authors of the Work (as defined in the below author licence), an exclusive licence and/or a non-exclusive licence for contributions from authors who are: i) UK Crown employees; ii) where BMJ has agreed a CC-BY licence shall apply, and/or iii) in accordance with the terms applicable for US Federal Government officers or employees acting as part of their official duties; on a worldwide, perpetual, irrevocable, royalty-free basis to BMJ Publishing Group Ltd ("BMJ") its licensees and where the relevant Journal is co-owned by BMJ to the co-owners of the Journal, to publish the Work in this journal and any other BMJ products and to exploit all rights, as set out in our licence.

The Submitting Author accepts and understands that any supply made under these terms is made by BMJ to the Submitting Author unless you are acting as an employee on behalf of your employer or a postgraduate student of an affiliated institution which is paying any applicable article publishing charge ("APC") for Open Access articles. Where the Submitting Author wishes to make the Work available on an Open Access basis (and intends to pay the relevant APC), the terms of reuse of such Open Access shall be governed by a Creative Commons licence – details of these licences and which Creative Commons licence will apply to this Work are set out in our licence referred to above.

Other than as permitted in any relevant BMJ Author's Self Archiving Policies, I confirm this Work has not been accepted for publication elsewhere, is not being considered for publication elsewhere and does not duplicate material already published. I confirm all authors consent to publication of this Work and authorise the granting of this licence.

For peer review only

Ethnicity and impact on the implementation of CBT in people with psychosis or bipolar disorder: A cohort study

Rohan Morris[1,2] William Sellwood[1] Dawn Edge[3] Craig Colling[4,5] Robert Stewart[45] Caroline Cupitt[5] Jayati Das-Munshi[45]

[1] Division of Health Research, Lancaster University, UK

[2] Lancashire Care Foundation Trust, Lancashire, UK

[3]Division of Psychology & Mental Health, School of Health Sciences, University of Manchester

[4] Institute of Psychiatry, Psychology and Neuroscience, King's College London, London, United Kingdom

[5] South London & Maudsley NHS Foundation Trust, London, UK

Corresponding author contact details: Dr Rohan Michael Morris, r.morris1@lancaster.ac.uk, Lancaster University, Furness College, Department of Clinical Psychology, Division of Health Research, LA1 4YW, +44 (0)1524 592691

Keywords: Psychotic Disorders, Mood Disorders, Psychotherapy, Ethnic Groups, Cognitive Behavioral Therapy

Word count 4910

Ethnicity and impact on the implementation of CBT

For peer review only

Ethnicity and impact on the implementation of CBT

Abstract

Objectives: 1) To explore the role of ethnicity in accessing Cognitive Behavioural Therapy (CBT) for people with psychosis or bipolar disorder whilst adjusting for differences in risk profiles and symptom severity. 2) To assess whether the context of treatment (inpatient versus community) impacts on the relationship between ethnicity and access to CBT.

Design: Cohort study of case-register data from one catchment area (January 2007 to July 2017).

Setting: A large secondary care provider serving an ethnically-diverse population in London.

Participants: Data extracted for 30,497records of people who had diagnoses of bipolar disorder (ICD Code F30-1) or psychosis (F20-F29 excluding F21). Exclusion criteria were: <15 years old, missing data, and not self-defining as belonging to one of the larger ethnic groups. The sample (N=20010) comprised the following ethnic groups: White British, n=10393; Black Caribbean, n=5481, Black African, n=2817; Irish, n= 570; and 'South Asian' people (consisting of Indian, Pakistani, and Bangladeshi people) n=749.

Outcome Assessments: Odds ratios for receipt of CBT (single session or full course) as determined via multivariable logistic regression analyses.

Results: In models adjusted for risk and severity variables, in comparison to White British people; Black African people were less likely to receive a single session of CBT (OR 0.73, CI 0.66 to 0.82, p<.001); Black Caribbean people were less likely to receive a minimum of 16 sessions of CBT (OR 0.83, CI 0.71 to 0.98, p=.03); Black African people and Black Caribbean people were significantly less likely to receive CBT whilst inpatients (respectively OR 0.76, CI 0.65 to 0.89, p=.001; OR 0.83, CI 0.73 to 0.94, p=.003).

For peer review only

Ethnicity and impact on the implementation of CBT

Conclusions: This study highlights disparity in receipt of CBT for Black African and Caribbean people. This study also highlights that context of therapy (inpatient versus community settings) impacts on disparity in access to treatment.

Strengths and Limitations

- A key strength of this study is that the data were from a near-complete case register of a large secondary care mental health service provider; which has a near monopoly on mental health provision in its catchment area.
- Published data are available on the tools used for extracting information about CBT which indicates high degrees of precision (95%) and sensitivity (96%).
- A limitation of this study is that it was not possible to assess access to other types of psychological intervention (e.g. Family Therapy).
- This study was not able to assess the offer of therapy (only receipt), consequently it is unclear if there are ethnic differences in whether or not therapy is offered to Black service users.

For peer review only

Ethnicity and impact on the implementation of CBT

Introduction

Background

There are ethnic differences in the care pathways and treatments people with psychosis receive. Within the UK, people of Black Caribbean and Black African descent are more likely to: enter mental health services via forensic pathways and experience compulsory detention (Mann et al., 2014), receive medication by depot (Das-Munshi, Bhugra, & Crawford, 2018a), and be subject to Community Treatment Orders (CTO) (Evans, Makala, Humphreys & Mohan, 2010). Black people with treatment resistant schizophrenia are less likely to receive drug treatments in accordance with national guidelines and Asian British people with a schizophrenia diagnosis are less likely to receive copies of their care plans (Das-Munshi et al., 2018a). Treatment inequalities based on ethnicity have also been identified in other countries. For example, in the USA, people of African descent have less money spent on their healthcare through state funded programs (Horvitz-Lennon, McGuire, Alegria, & Frank, 2009) and are less likely to receive medication associated with fewer side effects (Daumit et al., 2003). In the Netherlands, ethnic minority groups are more likely to be compulsorily detained for treatment and less likely to be recommended for outpatient treatment (Vinkers, de Beurs, Barendregt, Rinne, & Hoek, 2010).

A prospective study in the UK, found significant ethnic differences in Mental Health Act 2007 (MHA) assessments and detentions, with Black Africans having higher rates than any other ethnic group (Gajwani, Parsons, Birchwood, & Singh, 2016). However, Gajwani et al., (2016) found that when controlling for diagnosis, age, risk and social support there were no significant ethnic differences in detention. Similarly, Singh et al., (2014) found no significant differences between ethnic groups in MHA detention whilst controlling for variables such as risk and social support. These studies raise the possibility that treatment

For peer review only

differences could be accounted for by ethnic differences in factors such as: self-harm and suicide attempt (Al-Sharifi, Krynicki, & Upthegrove, 2015), psychosis symptom profiles (Veling, Selten, Mackenbach, & Hoek, 2007), deprivation (Jivraj & Khan, 2013), and substance use (Shih, Miles, Tucker, Zhou & D'Amico, 2010).

UK national guidelines recommend Cognitive Behavioural Therapy for the treatment and prevention of psychosis (CBTp), as CBTp has demonstrated robust evidence of its efficacy on service-user outcomes (NICE, 2014a). However, the National Audit of Schizophrenia (Royal College of Psychiatrists, 2014) found that CBTp was only offered to 39% of service users and accessed by 19% of service users. There are evidently barriers to accessing CBTp (e.g. Hazell, Strauss, Cavanagh, & Hayward, 2017; Prytys, Garety, Jolley, Onwumere, & Craig, 2011) although certain factors may increase referral to CBTp (e.g. higher levels of positive symptoms; Fanning et al., 2012).

People from ethnic minority communities experience additional barriers to access and engagement with psychological therapy more generally (Memon et al., 2016). In the UK, people of Black Caribbean and Black African descent with psychosis are less likely to receive a talking therapy than their White British counterparts (Colling et al., 2017; McKenzie et al., 1995; McKenzie et al., 2001). A nationally representative survey of people with psychosis found that all ethnic minority groups (excluding those with Mixed ethnicity) were less likely to be offered CBT; and Black service users were less likely to be offered Family Therapy (Das-Munshi et al., 2018a). Similar findings have been demonstrated in international samples, where Black Americans with psychosis are less likely to receive a talking therapy than their White American counterparts (Walkup, Wei, Sambamoorthi, Crystal, & Yanos, 2006).

For peer review only

Ethnicity and impact on the implementation of CBT

Engagement is a complex concept that requires the service provider being adequately engaging and the recipient to be adequately engaged. There are potentially many explanations of ethnic variations in access to and engagement with CBT. For example, ethnic minority communities have more coercive pathways into treatment (e.g. Mann et al., 2014) which may adversely influence the therapeutic relationship (Sweeney et al., 2014), and subsequently impact on engagement in treatment (Holdsworth, Bowen, Brown & Howat, 2016). Other barriers to engagement might include: lower socio-economic status (Barrett et al., 2008); increased stigma in certain communities (Mantovani, Pizzolati, & Edge, 2017); fear of service-users by providers, and fear of providers by service-users (Keating, Robertson, McCulloch & Francis, 2002); suspiciousness of mental health services and non-culturally appropriate therapy (Rathod, Kingdon, Phiri, & Gobbi, 2010); language barriers (Chen & Rizzo, 2010); clinicians' perceptions of religious and spiritual explanations for psychosis (Islam, Rabiee & Singh, 2017); and institutional racism within mental health services (Blofeld, 2003; Royal College of Psychiatrists, 2018).

Research Questions and rationale:

There is a lack of information about the extent of inequalities experienced by ethnic minority groups with serious mental illness, despite well-recognised adverse outcomes in certain minority groups. Furthermore, there is a paucity of information about the role that risk and symptom severity plays in treatment disparity (including access to psychological therapy) for ethnic minority groups. Consequently, using all the case records from a large secondary care mental healthcare provider, this study set out to answer the following questions:

1: In people who have had a diagnosis of bipolar disorder (ICD-10 code F30-1) or psychosis (ICD-10 code F20-29 excluding F21), are there variations by ethnic-group in receipt of CBT after adjustment for differences in risk profiles and symptom severity?

For peer review only - http://bmjopen.bmj.com/site/about/guidelines.xhtml

Ethnicity and impact on the implementation of CBT

2: Do ethnic-group variations in receipt of CBT differ between contexts (e.g. inpatient versus community settings) after adjustment for risk profiles and symptom severity?

Method

Study Design and Setting

The data, which were generated as part of routine care, were derived from clinical records from South London and Maudsley (SLaM) Trust. SLaM is a near-monopoly provider of secondary mental health services (Lally et al., 2015) for a catchment of over 1.2 million residents in south London and has over 400,000 service user records (Perera et al., 2016). The SLaM catchment boroughs are not dissimilar from London as a whole in terms of age, education, gender and socioeconomic status (Perera et al., 2016; Stewart et al., 2009). However, SLaM has a higher proportion of ethnic minority groups in comparison to England as whole (Perera et al., 2016). Even after adjustment for age, sex and ethnicity, areas within SLaM's catchment have been shown to have a 2.2 times higher incidence of psychosis than the European average (Jongsma et al., 2017).

This investigation utilised the Clinical Record Interactive Search (CRIS) tool (Perera et al., 2016) to access an anonymised data set derived from SLaM's electronic health records that comprise the Maudsley Biomedical Research Centre (BRC) Case Register. The BRC Case Register utilises an opt-out mechanism, which is seldom used (circa N=4). Consequently, the sampling techniques employed ensure that persons who have not experienced good engagement with mental health services are still represented in the sample. Established in 2008, the CRIS system facilitates access and retrieval of anonymised clinical records. For a more in-depth description of how the data are stored, anonymised, and accessed see Stewart et al., (2009), Fernandes et al., (2013) and Perera et al., (2016).

For peer review only

Ethnicity and impact on the implementation of CBT

Sample

Cases were included if they had received an ICD-10 diagnosis of a bipolar related mental health problem (i.e. manic episode [F30] and/or bipolar affective disorder [F31]) and were defined as having a bipolar disorder. The psychosis group included anyone with any of the following diagnoses: schizophrenia [F20], delusional disorder [F22], brief psychotic disorder [F23], shared psychotic disorder [F24], schizoaffective disorder [F25], other nonorganic psychotic disorders [F28] and unspecified nonorganic psychosis [F29].

Cases were excluded if: they were under the age of 15; they had a diagnosis of an organic/non-functional disorder; or there were missing data regarding marital status, ethnicity, IMD score, gender, or age. Due to limited numbers in some ethnic groups, cases were excluded if their recorded ethnicity did not belong to one of the following Office of National Statistics (ONS, N.D.) categories: Black African, Black Caribbean, Irish, and White British. A group labelled 'South Asian' including individuals recorded as Indian, Pakistani, or Bangladeshi was also included in the sample. This investigation utilised the same approach of defining and grouping ethnicity which has been applied to CRIS data previously (Das-Munshi et al., 2016; 2018b).

Data Retrieval

SLaM adopted fully electronic health records for all its services in 2006, including the importing of legacy data. The current data set includes records from the 1st of January 2007 up until the extraction date of the 31st of July 2017. Source clinical records contain information from structured closed question response boxes (e.g. age) and free text. Automated natural language processing (NLP) algorithms (see Aggarwal & Zhai, 2012) are used to determine the presence and prescribed 'value' of variables contained in free text.

For peer review only - http://bmjopen.bmj.com/site/about/guidelines.xhtml

Ethnicity and impact on the implementation of CBT

Within the current investigation, NLP algorithms were used to provide supplementary information on diagnoses and CBT. Recording an ICD 10 (WHO, 2010) diagnosis within a structured field is mandatory within SLaM (Jackson et al., 2017), supplemented by NLP to ascertain diagnoses recorded in free text sources e.g. clinical notes (Jackson et al., 2017; Perera et al., 2016). Another NLP algorithm has been developed to identify case notes that document a CBT session (Colling et al., 2017), again supplementing information within structured fields and achieving in combination a positive predictive value of 95% and a sensitivity of 96% (Colling et al., 2017).

Demographic, Clinical and Treatment data extracted and operationalised

Demographic data retrieved included gender, marital status, ethnicity and age. All of the demographic data was retrieved at the point of data extraction (31st July 2017), for example the participants age on the 31st of July 2017. From Lower Super Output Area (LSOA) of residence, a standard national geographic unit containing approximately 1500 residents, area level deprivation was calculated from the Index of Multiple Deprivation (IMD) (Department for Communities and Local Government, 2015). Multiple area level assessments contribute to seven subscales (Income Deprivation; Employment Deprivation; Education, Skills and Training Deprivation; Health Deprivation and Disability; Crime; Barriers to Housing and Services; and Living Environment Deprivation) which form the IMD. Scores on the IMD were split into deciles within the current sample.

The algorithm within the SLaM clinician interface ensures that structured risk assessments are completed when risk information is noted. We developed an assessment of severity and risk based on previous approaches used with this dataset (Woodhead et al., 2016). To this end, we retrieved information from structured risk assessments pertaining to: history of violence, history of ‘non-adherence’, history of suicide attempt, perceived lethal means used in suicide attempt, current plans to end life, expression of suicidal ideation,

expressed feelings of hopelessness, expressed high levels of subjective distress, and expressed feelings of having no control. We also retrieved information about previous: substance use disorder diagnosis (ICD code F1), inpatient admissions, treatment under the Mental Health Act, A&E attendance (for mental health problems), referral to Assertive Outreach, referral to the crisis team, and forensic history.

We retrieved data about the CBT session regarding: whether the service user was an inpatient or outpatient at the time of contact; whether the contact was face-to-face or remote (e.g. via telephone); and whether the contact was in a one to one, or group session. In line with National Standard guidelines definition of access (NHS England, 2016), the current investigation assessed whether participants had at least one documented session of CBT. NICE guidelines for psychosis recommend that CBT is delivered "over at least 16 planned session (sic)" (NICE, 2014a, p.589). NICE guidelines for bipolar disorder recommend that a depressive episode should be treated with between 16 to 20 sessions of CBT (NICE, 2014b). Consequently, a 16-session criterion was also adopted as a more stringent definition of a course of CBT. Jolley et al., (2015) operationalised CBT therapy completion as at least 5 sessions. Supplementary analyses were conducted utilising this less stringent definition of a course of CBT treatment. Analyses of the 5 and 16 session criteria were restricted to participants who had at least one documented session of CBT (n=5197). Participants were also excluded from analyses regarding the 5 and 16 session criteria if they were currently receiving CBT at data extraction and had not received a minimum of 5 or 16 sessions of CBT, which resulted in n=100 and n=220 participants being excluded respectively (see Figure 1). CBT that was currently on-going was defined as anyone who had a CBT session in the 6 weeks prior to data extraction.

For peer review only

Ethnicity and impact on the implementation of CBT

Ethical Considerations

The anonymised dataset has been approved by the NHS REC for secondary analysis (Oxford C Research Ethics Committee, reference18/SC/0372). This particular project received ethical approval from the Lancaster University Faculty of Health and Medicine Research Ethics Committee and approval from the CRIS Oversight Committee.

Patient and Public Involvement

This specific project was reviewed, commented on and approved by the CRIS Oversight Committee, which is chaired by a service user representative. Furthermore, the development of the CRIS system was informed by consultation with service users (Fernandes et al., 2013).

Analysis

Logistic regression models were built using multivariable procedures in Stata 12. Models were adjusted for demographic data (gender, age, IMD, and marital status), diagnoses (psychosis/bipolar disorder), and risk/severity variables (as described previously). Analysesare presented as: crude associations; adjustments for demographic data and diagnosis (Step 1); and adjustments for demographic data, diagnosis and the risk/severity variables (Step 2).

Results

Descriptive Statistics

The final sample consisted of 20,010 cases, Figure 1 displays the flow of cases through the study.

(FIGURE 1)

For peer review only

Ethnicity and impact on the implementation of CBT

The majority of cases were White British (n = 10393, 51.9%), the next largest ethnic group were Black Caribbean people who made up 27.4% of the sample (n=5481). There were more male cases (n=10457, 52.3%) than female and the majority were single (n=17097, 85.4%). Table 1 summarises the demographic and diagnosis data (at the time of data extraction) with relevant proportions for each ethnic group. Further information on treatment, risk, and severity including items from the structured risk assessment can be found in Supplementary Table 1.

(TABLE 1)

Just over a quarter of the sample (26.0%, n=5197) had a documented session of CBT in the study period. The median number of sessions of CBT was 5 (IQR 13). Considering all CBT sessions documented, most were delivered face to face, at a ratio of approximately 30 face to face sessions for every 1 remote (e.g. telephone) session delivered, and as individual rather than group sessions at a ratio of approximately 17:1. Of the people who had received CBT, 30% had their first ever (documented) session as an inpatient, 55.4% had ≥5 sessions and 25.8% had received ≥16 sessions. Further information about CBT can be found in Supplementary Table 2.

Ethnicity and reported receipt of CBT as an inpatient or outpatient.

Table 2 displays the unadjusted and adjusted ORs for having a reported session of CBT in relation to ethnicity and covariates. The final adjusted model indicated that the Black African group were significantly less likely to receive CBT than the White British group (OR 0.73, CI 0.66 to 0.82, p<.001), after risk indicators were taken into account. In the adjusted model, several factors related to risk and severity were independently associated with increased likelihood of reported receipt of CBT, including lifetime inpatient admission, history of non-adherence, history of suicide attempt, lethal means used in suicide attempt,

For peer review only

suicidal ideation, feelings of hopelessness, high levels of distress, no feelings of control, and referral to the crisis team. However, a history of a substance misuse disorder diagnosis and plans to end life were associated with a decreased likelihood of reported receipt of CBT.

(TABLE 2)

Ethnicity and a minimum of 16 CBT sessions

Table 3 displays the unadjusted and adjusted ORs of receiving a minimum of 16 sessions of CBT in relation to ethnicity and covariates. The adjusted model indicated that the Black Caribbean group were significantly less likely to receive a minimum of 16 sessions of CBT than the White British group (OR 0.83, CI 0.71 to 0.98, p=.03). The model also indicated that receiving the first session of CBT as an inpatient was associated with decreased odds of having at least 16 sessions of CBT (OR 0.35, CI 0.29 to 0.42, p<.001) and some of the indicators of risk increased the odds of receiving CBT (history of suicide attempt, reported high levels of distress and lifetime referral to crisis team). However, several factors associated with increased odds of ever receiving a documented session of CBT (Table 2) were not significantly associated with having a minimum of 16 documented sessions (i.e. lifetime inpatient admittance, history of non-adherence, lethal means used in suicide attempt, reported suicidal ideation, reported feelings of hopelessness, reported feelings of a lack of control).

TABLE 3

Ethnicity and reported receipt of CBT as an inpatient

Analyses were restricted to participants who had been an inpatient (N= 9417) and associations investigated with receipt or not of CBT in this setting. Unadjusted and adjusted associations are displayed in Table 4. The adjusted model demonstrated that the Black African group (OR 0.76, CI 0.65 to 0.89, p=.001) and the Black Caribbean group (OR 0.83,

For peer review only

Ethnicity and impact on the implementation of CBT

CI 0.73 to 0.94, p=.003) were significantly less likely to have received CBT than the White British group.

TABLE 4

Supplementary Analyses

Analyses using the less stringent definition of a course of CBT (≥5 sessions) indicated the Black African group were significantly less likely to receive this in comparison to the White British group (OR 0.76, CI 0.63 to 0.91, p=.003) (see Supplementary Table 3). Analyses of CBT sessions received only as an outpatient also indicated that the Black African group (OR 0.75, CI 0.67 to 0.84, p<.001) were significantly less likely to receive this than the White British group (see Supplementary Table 4).

Post-Hoc Sensitivity Analysis

1. Recording of clinical risk

The crude estimates indicated that several variables indicative of higher clinical risk and severity were associated with increased odds of having a (single) documented session of CBT (Table 2). We considered that this may be because CBT is better recorded (rather than more likely to be delivered) for those at an increased risk (e.g. of harming themselves, suicide, harming others) and proposed that, if defensive practice resulted in better note keeping, this would be most likely evident in the structured fields. Consequently, as a supplementary sensitivity analysis, using the entire sample (N=20,010), models assessing reported receipt of CBT were re-run omitting entries identified in the structured fields, (i.e. just using data derived from free text). However, this analysis continued to indicate an association between Black African group membership and significantly lower odds of

For peer review only

Ethnicity and impact on the implementation of CBT

receiving CBT than White British group membership (OR 0.76, CI 0.63 to 0.92, p=.004). Adjusted and unadjusted odds ratios are presented in Supplementary Table 5.

2. Influence of time

Additional analyses were conducted to assess if changes over time affected referral practices for psychological treatments. To this end, a variable was created indicating participants who had received a diagnosis of psychosis or bipolar affective disorder after the mid-point of the data collection window (i.e. after the 16th of April 2012). Models considering ethnicity and reported receipt of CBT were re-run including the variable indicating the date at which diagnosis was given. This analysis also indicated that the Black African group were significantly less likely to receive CBT than the White British group (OR 0.72, CI 0.65 to 0.81, p<.001), suggesting that this finding was not influenced by the date diagnosis was given (see Supplementary Table 6). In the fully adjusted model, receiving a diagnosis of psychosis or bipolar affective disorder after the midpoint of the data collection window was associated with decreased odds of a documented session of CBT (OR 0.77, CI 0.71 to 0.83, p<.001). Further, analysis was conducted to assess if there was an interaction between time and ethnicity; however, a likelihood ratio test indicated that fitting this interaction term did not significantly improve the model: Chi^2 (4) =5.25, p= .26.

Discussion

This investigation found that after adjustment for numerous indicators of risk and severity, in comparison to White British counterparts, Black African people with bipolar disorder or psychosis were less likely to have a documented session of CBT, a finding which was robust to a number of sensitivity analyses. After adjustment for indicators of risk and symptom severity in comparison to White British people, Black Caribbean people were also less likely to receive CBT as inpatients, and were less likely to receive the minimum 16

sessions recommended by national guidelines. This study also found that regardless of ethnicity people who had their first documented session of CBT as an inpatient were less likely to receive a minimum of 16 sessions of CBT (and a similar effect was also noted in supplementary analyses of a minimum 5 documented sessions and documented receipt of CBT as an outpatient). In addition, regardless of ethnicity indicators of higher risk and severity of symptoms were typically associated with higher odds of receiving CBT; however, these associations between risk status and receipt of CBT were less consistent in analyses of a minimum 16 documented sessions.

To our knowledge, this study has used the largest sample to date to assess ethnic differences in access to CBT for people with psychosis or bipolar affective disorder. This study utilised a case register from a large mental healthcare provider serving a socially and ethnically diverse geographic catchment. Furthermore, the data were sourced from the full electronic health record, using a case register with near-complete coverage of people receiving mental healthcare for these diagnoses. The study utilised a tool to extract information about CBT from structured fields and free text, an approach which has been shown to have high positive predictive value and sensitivity values in previous work (Colling et al., 2017). Consequently, this study likely provides a highly accurate picture of access to CBT delivered by mental health services within the catchment. Of note, despite having recognised high incidence rates of psychosis (Jongsma et al., 2017), the catchment is not dissimilar to other parts of London and UK urban areas on several sociodemographic metrics (Stewart et al., 2009; Perera et al., 2016); the results of this investigation may generalise to other urban and semi-urban multicultural areas in England. A notion which is supported by ethnic disparity in access to therapy indicated in nationally representative data (Das-Munshi et al., 2018). By accessing a large data set of complete clinical records we were able to contribute novel findings relating to the impact of risk and pathways on engagement with

For peer review only

Ethnicity and impact on the implementation of CBT

CBT. However, one limitation of this investigation is that it was not possible to extract information from the BRC Case Register about other psychological therapies, some of which are recommended by national guidelines and delivered routinely within the service analysed (e.g. Family Intervention; NICE, 2014a). It is possible therefore that disparity in access to CBT may be accounted for by ethnic differences in preference for therapy type, although this has not been suggested to be the case in other studies of national data from the UK (Das-Munshi et al., 2018a). Another limitation is that although this study likely displays an accurate picture of service users who *received* CBT it was not possible to derive information about the *offer* of CBT.

Our findings replicate those observed for unselected community residents from a nationally representative sample, namely less equitable access to CBT for ethnic minority groups (Das-Munshi et al., 2018a). Previous investigations which have explored ethnic disparities in access/engagement with CBT in samples with psychosis have not differentiated between Black African and Black Caribbean people (Colling et al., 2017; Das Munshi et al., 2018a; Jolley et al., 2015), despite the two groups typically having different migratory histories and different factors influencing pathways into treatment for psychosis (Bhui, Ullrich, Kallis & Coid, 2015). The current investigation was able to define more specific ethnic categories providing a more nuanced understanding of ethnicity and access to CBT.

Previous research has highlighted that more positive symptoms in psychosis increase referrals for CBT (Fanning et al., 2012). Our study extended this finding by highlighting that numerous indicators of higher symptom severity and risk increase the propensity to receive a minimum of one session of CBT. However, despite controlling for these variables, this study found persistent disparities by ethnicity in receipt of CBT (i.e. a minimum of one documented session). The relationship between risk and CBT engagement (i.e. documented receipt of a minimum of 16 sessions) appeared less consistent. Several of the risk indicators which

For peer review only

Ethnicity and impact on the implementation of CBT

increased the odds of receiving one documented session of CBT were not significantly associated either way with receipt of a minimum of 16 sessions. This may suggest a more complex relationship between risk and CBT engagement. The positive association between recorded level of clinical risk and receipt of CBT is in contrast to research suggesting that inequalities between ethnic groups in mental health treatment could be caused by differences in symptom severity (Gajwani et al., 2016; Singh et al., 2014). Despite risk indicators (typically) increasing access to CBT and previous investigations suggesting that Black women are most likely to self-harm (Cooper et al., 2010); the current investigation does not indicate that ethnic disparities in the receipt of CBT is as a consequence of ethnic differences in risk or symptom profile.

First access of CBT as an inpatient was associated with lower odds of receiving further CBT sessions. There are numerous potential explanations. For example, coercive practice in inpatient settings has been well documented and this may potentially impact on subsequent engagement (Department of Health and Social Care, 2018). Alternatively, our finding may be related to differences in recovery styles (Drayton, Birchwood & Trower, 1998). An avoidant recovery style (referred to as sealing over) has been linked to poorer engagement with services (Tait, Birchwood & Petertrower, 2004), and it is possible that some people are receptive to psychological therapy at the point of crisis (i.e. during inpatient stay), but once there is a diminution of symptoms they 'seal over' which reduces engagement.

Our study suggests that, within clinical settings, further work is needed to ensure there is parity in access to CBT. In practice, this might include ensuring that CBT is systematically offered to groups who are less likely to receive treatment. It is also feasible that further work is needed to ensure that CBT is more acceptable to Black groups which might be achieved by culturally-adapting interventions (e.g. Rathod et al., 2013). Nonetheless, more research is required to explore the reasons underpinning ethnicity difference in access to CBT, whether

For peer review only

ethnic differences in receipt of CBT extend to the offer of CBT, and the impact clinical risk has on engagement with CBT. Moreover, further research is necessary to explore the impact of pathways into care or psychological treatment, and its role in subsequent engagement.

For peer review only

Ethnicity and impact on the implementation of CBT

Acknowledgements

We are grateful to Hitesh Shetty for his work extracting data from the BRC case register and his advice on usage of BRC CRIS data. We are grateful to Megan Pritchard for her advice on usage of BRC CRIS data and her support in training RM on accessing CRIS data. We are grateful to Dr Emmanuelle Peters for her advice about the study and her advice about psychological therapy service delivery within SLaM.

Declaration of Funding

JD is funded by the Health Foundation working together with the Academy of Medical Sciences. RS is part-funded by the National Institute for Health Research (NIHR) Biomedical Research Centre at South London and Maudsley NHS Foundation Trust and King's College London, and by an NIHR Senior Investigator award. RM is funded by Lancashire Care Foundation Trust. The authors declare that the study funders have had no role in the study design; in the collection, analysis and interpretation of the data; in the writing of the report; and in the decision to submit the paper for publication.

This paper represents independent research funded by the National Institute for Health Research (NIHR) Biomedical Research Centre at South London and Maudsley NHS Foundation Trust and King's College London. The views expressed are those of the author(s) and not necessarily those of the NHS, the NIHR or the Department of Health and Social Care

Competing Interests

SLaM and its services have had no role, in the study design, in the analysis and interpretation of the data, in the writing of the report, or in the decision to submit the paper for publication. CC is employed by SLaM and works within one of SLaM's services which

For peer review only

has produced some of the clinical notes that form part of the data analysed herein. RS declares research funding within the last 5 years from Roche, GSK and Janssen.

Contributorship statement

RM, JD, WS, and DE made substantial contributions to the conception and design of the work and interpretation of the data. RM and JD made a substantial contribution to the analysis of the data. RS made a substantial contribution to the conception and design of the work and acquisition of the data. Craig Colling made a substantial contribution to the acquisition of the data. Caroline Cupitt made a substantial contribution to the interpretation of the data. All authors contributed to reviewing, drafting and revising of the manuscript. All authors have provided their approval for the work to be published and are in agreement to be accountable for all aspects of the work in ensuring that questions related to the accuracy or integrity of any part of the work are appropriately investigated and resolved.

Data sharing statement

Data are owned by the South London and Maudsley NHS Foundation Trust (SLaM) which provides access to anonymised data derived from electronic medical records via the Clinical Record Interactive Search (CRIS) system. These data can only be accessed by permitted individuals from within a secure firewall (i.e. remote access is not possible and the data cannot be sent elsewhere) in the same manner as the authors. For data requests please contact Cris.administrator@slam.nhs.uk

For peer review only

Ethnicity and impact on the implementation of CBT

References

Aggarwal, C. & Zhai, C. (2012). *Mining text data*. New York, USA: Springer

Al-Sharifi, A., Krynicki, C. R., & Upthegrove, R. (2015). Self-harm and ethnicity: A systematic review. *The International Journal of Social Psychiatry*, *61*(6), 600–612. https://doi.org/10.1177/0020764015573085

Barrett, M. S., Chua, W.-J., Crits-Christoph, P., Gibbons, M. B., Casiano, D., & Thompson, D. (2008). Early withdrawal from mental health treatment: Implications for psychotherapy practice. *Psychotherapy (Chicago, Ill.)*, *45*(2), 247–267. http://dx.doi.org/10.1037/0033-3204.45.2.247

Bhui, K., Ullrich, S., Kallis, C., & Coid, J. W. (2015). Criminal justice pathways to psychiatric care for psychosis. *The British journal of psychiatry*, *207*(6), 523–529. http://doi:10.1192/bjp.bp.114.153882

Blofeld, J. (2003). *Independent Inquiry into the death of David Bennett.* Cambridgeshire, Norfolk, Suffolk and Cambridgeshire, UK: Strategic Health Authority

Chen, J., & Rizzo, J. (2010). Racial and ethnic disparities in use of psychotherapy: evidence from U.S. national survey data. *Psychiatric Services (Washington, D.C.)*, *61*(4), 364–372. https://doi.org/10.1176/ps.2010.61.4.364

Colling, C., Evans, L., Broadbent, M., Chandran, D., Craig, T. J., Kolliakou, A., … Garety, P. A. (2017). Identification of the delivery of cognitive behavioural therapy for psychosis (CBTp) using a cross-sectional sample from electronic health records and open-text information in a large UK-based mental health case register. *BMJ Open*, *7*(7). http://dx.doi.org/10.1136/bmjopen-2016-015297

For peer review only

Cooper, J., Murphy, E., Webb, R., Hawton, K., Bergen, H., Waters, K., & Kapur, N. (2010). Ethnic differences in selfharm, rates, characteristics and service provision: Three city cohort study. *The British Journal of Psychiatry, 197*, 212–218. http://doi:10.1192/bjp.bp.109.072637

Das-Munshi, J., Ashworth, M., Gaughran, F., Hull, S., Morgan, C., Nazroo, J., … Prince, M. (2016). Ethnicity and cardiovascular health inequalities in people with severe mental illnesses: protocol for the E-CHASM study. *The International Journal for Research in Social and Genetic Epidemiology and Mental Health Services*, *51*(4), 627–638. https://doi.org/10.1007/s00127-016-1185-8

Das-Munshi, J., Bhugra, D. K. M. L., & Crawford, M. (2018a). Ethnic minority inequalities in access to treatments for schizophrenia and schizoaffective disorders. Findings from a nationally representative cross-sectional study. *BMC Medicine*.

Das-Munshi, J., Chang, C.-K., Dutta, R., Morgan, C., Nazroo, J., Stewart, R., & Prince, M. J. (2018b). Ethnicity and excess mortality in severe mental illness: a cohort study. *The Lancet Psychiatry*, *4*(5), 389–399. https://doi.org/10.1016/S2215-0366(17)30097-4

Daumit, G. L., Crum, R. M., Guallar, E., Powe, N. R., Primm, A. B., Steinwachs, D. M., & Ford, D. E. (2003). Outpatient prescriptions for atypical antipsychotics for African Americans, Hispanics, and whites in the United States. *Archives of General Psychiatry*, *60*(2), 121–128. https://doi:10.1001/archpsyc.60.2.121

Department of Health and Social Care (2018). *Modernising the Mental Health Act: increasing choice, reducing compulsion*. London, England: Home Office.

Ethnicity and impact on the implementation of CBT https://assets.publishing.service.gov.uk/government/uploads/system/uploads/attachment_data/file/762206/MHA_reviewFINAL.pdf

Drayton, M., Birchwood, M., & Trower, P. (1998). Early attachment experience and recovery from psychosis. *The British Journal of Clinical Psychology*, 37 (Pt 3), 269–284. https://doi.org/10.1111/j.2044-8260.1998.tb01385.x

Evans, R., Makala, J., Humphreys, M., & Mohan, C. R. N. (2010). Supervised community treatment in Birmingham and Solihull: first 6 months. *The Psychiatrist*, *34*(8), 330–333. https://doi.org/DOI:10.1192/pb.bp.109.027482

Fanning, F., Foley, S., Lawlor, E., McWilliams, S., Jackson, D., Renwick, L., … O'Callaghan, E. (2012). Group cognitive behavioural therapy for first episode psychosis: who's referred, who attends and who completes it? *Early Intervention in Psychiatry*, *6*(4), 432–441. https://doi.org/10.1111/j.1751-7893.2011.00333.x

Fernandes, A.C., Cloete, D., Broadbent, M., Hayes, R.D., Chang, C-K., Jackson, R.G., Roberts, A., Tsang, J., Soncul, M., Liebscher, J., Stewart, R. & Callard, F. (2013) Development and Evaluation of a De-Identification Procedure for a Case Register Sourced from Mental Health Electronic Records. *BMC Psychiatry.* 13, 71. https://doi.org/10.1186/1472-6947-13-71

Gajwani, R., Parsons, H., Birchwood, M., & Singh, S. P. (2016). Ethnicity and detention: are Black and minority ethnic (BME) groups disproportionately detained under the Mental Health Act 2007? *Social Psychiatry and Psychiatric Epidemiology*, *51*(5), 703–711. https://doi.org/10.1007/s00127-016-1181-z

Hazell, C. M., Strauss, C., Cavanagh, K., & Hayward, M. (2017). Barriers to disseminating brief CBT for voices from a lived experience and clinician

For peer review only

perspective. *PloS One*, *12*(6), e0178715. https://doi.org/10.1371/journal.pone.0178715

Holdsworth, E., Bowen, E., Brown, S., & Howat, D. (2016). The Development of a Program Engagement Theory for Group Offending Behavior Programs. *International Journal of Offender Therapy and Comparative Criminology*, *61*(13), 1479–1499. https://doi.org/10.1177/0306624X15624177

Horvitz-Lennon, M., McGuire, T. G., Alegria, M., & Frank, R. G. (2009). Racial and ethnic disparities in the treatment of a Medicaid population with schizophrenia. *Health Services Research*, *44*(6), 2106–2122. https://doi.org/10.1111/j.1475-6773.2009.01041.x

Islam, Z., Rabiee, F., & Singh, S. P. (2015). Black and Minority Ethnic Groups' Perception and Experience of Early Intervention in Psychosis Services in the United Kingdom. *Journal of Cross-Cultural Psychology, 46*(5), 737-753. https://doi:10.1177/0022022115575737

Jackson, R. G., Patel, R., Jayatilleke, N., Kolliakou, A., Ball, M., Gorrell, G., … Stewart, R. (2017). Natural language processing to extract symptoms of severe mental illness from clinical text: the Clinical Record Interactive Search Comprehensive Data Extraction (CRIS-CODE) project. *BMJ Open*, *7*(1). http://dx.doi.org/10.1136/bmjopen-2016-012012

Jivraj, S. & Khan, O. (2013) *Ethnicity and deprivation in England: How likely are ethnic minorities to live in deprived neighbourhoods?* Centre on Dynamics of Ethnicity briefing paper. Retrieved on February 1, 2018 from http://hummedia.manchester.ac.uk/institutes/code/briefingsupdated/ethnicity-and-

For peer review only

deprivation-in-england-how-likely-are-ethnic-minorities-to-live-in-deprived-neighbourhoods%20(1).pdf

Jolley, S., Garety, P., Peters, E., Fornells-Ambrojo, M., Onwumere, J., Harris, V., … Johns, L. (2015). Opportunities and challenges in Improving Access to Psychological Therapies for people with Severe Mental Illness (IAPT-SMI): evaluating the first operational year of the South London and Maudsley (SLaM) demonstration site for psychosis. *Behaviour Research and Therapy*, *64*, 24–30. https://doi.org/10.1016/j.brat.2014.11.006

Jongsma, H.E., Gayer-Anderson, C., Lasalvia, A., Quattrone, D., Mulé, A., Szöke, A., … Kirkbride, J.B. (2018). Treated incidence of psychotic disorders in the multinational eu-gei study. *JAMA Psychiatry*, 75(1), 36–46. http://dx.doi.org/10.1001/jamapsychiatry.2017.3554

Keating, Robertson, McCulloch & Francis, (2002) *Breaking the Circles of Fear: A review of the relationship between mental health services and African and Caribbean communities*. London, UK: The Sainsbury Centre for Mental Health

Lally, J., Wong, Y. L., Shetty, H., Patel, A., Srivastava, V., Broadbent, M. T. M., & Gaughran, F. (2015). Acute hospital service utilization by inpatients in psychiatric hospitals. *General Hospital Psychiatry*, *37*(6), 577–580. https://doi.org/10.1016/j.genhosppsych.2015.07.006

Mann, F., Fisher, H. L., Major, B., Lawrence, J., Tapfumaneyi, A., Joyce, J., … Johnson, S. (2014). Ethnic variations in compulsory detention and hospital admission for psychosis across four UK Early Intervention Services. *BMC Psychiatry*, *14*(1), 256. https://doi.org/10.1186/s12888-014-0256-1

For peer review only

Mantovani, N., Pizzolati, M., & Edge, D. (2017). Exploring the relationship between stigma and help-seeking for mental illness in African-descended faith communities in the UK. *Health Expectations : An International Journal of Public Participation in Health Care and Health Policy*, *20*(3), 373–384. https://doi.org/10.1111/hex.12464

McKenzie, K., Samele, C., Horn, E., Tattan, T., VanOs, J. & Murray, R. (2001). Comparison of the outcome and treatment of psychosis in people of Caribbean origin living in the UK and British Whites. *The British Journal of Psychiatry*, *178*(2), 160-165. https://doi.org/10.1192/bjp.178.2.160

McKenzie, K., van Os, J., Fahy, T., Jones, P., Harvey, I., Toone, B., & Murray, R. (1995). Psychosis with good prognosis in Afro-Caribbean people now living in the United Kingdom. *BMJ : British Medical Journal*, *311*(7016), 1325–1328.

Memon, A., Taylor, K., Mohebati, L. M., Sundin, J., Cooper, M., Scanlon, T., & de Visser, R. (2016). Perceived barriers to accessing mental health services among black and minority ethnic (BME) communities: a qualitative study in Southeast England. BMJ Open, 6(11), e012337. https://doi.org/10.1136/bmjopen-2016-012337

National Health Service England (2016). *Implementing the Early Intervention in Psychosis Access and Waiting Time Standard: Guidance*. London, UK: NHS England: National Collaborating Centre for Mental Health, National Institute for Health and Care Excellence. https://www.england.nhs.uk/mentalhealth/wp-content/uploads/sites/29/2016/04/eip-guidance.pdf

For peer review only

Ethnicity and impact on the implementation of CBT

National Institute for Health and Care Excellence (2014a). *Psychosis and schizophrenia in adults: The NICE guideline on treatment and management (CG178).* London, UK: NICE

National Institute for Health and Care Excellence (2014b). *Bipolar disorder: assessment and management (CG185).* London, UK: NICE

Office for National Statistics (N.D.). *Measuring equality: A guide for the collection and classification of ethnic group, national identity and religion data in the UK.* Retrieved on October 4, 2019 from https://www.ons.gov.uk/methodology/classificationsandstandards/measuringequality/ethnicgroupnationalidentityandreligion

Perera, G., Broadbent, M., Chang, C-K., Downs, J., Dutta, R., Fernandes, A., Hayes, R., Henderson, M., Jackson, R., Jewell, A., Kadra, G., Little, R., Pritchard, M., Shetty, H., Tulloch, A. & Stewart, R. (2016) Cohort Profile of the South London and Maudsley NHS Foundation Trust Biomedical Research Centre (SLaM BRC) Case Register: Current Status and Recent Enhancements of an Electronic Mental Health Record Derived Data Resource. BMJ Open 6, 3 p1-22 e00872

Prytys, M., Garety, P. A., Jolley, S., Onwumere, J., & Craig, T. (2011). Implementing the NICE guideline for schizophrenia recommendations for psychological therapies: a qualitative analysis of the attitudes of CMHT staff. *Clinical Psychology & Psychotherapy*, *18*(1), 48–59. https://doi.org/10.1002/cpp.691

Rathod, S., Kingdon, D., Phiri, P., & Gobbi, M. (2010). Developing culturally sensitive cognitive behaviour therapy for psychosis for ethnic minority patients by exploration and incorporation of service users' and health professionals' views

For peer review only

and opinions. *Behavioural And Cognitive Psychotherapy, 38*(5), 511-533. doi:10.1017/S1352465810000378

Rathod, S., Phiri, P., Harris, S., Underwood, C., Thagadur, M., Padmanabi, U., & Kingdon, D. (2013). Cognitive behaviour therapy for psychosis can be adapted for minority ethnic groups: A randomised controlled trial. *Schizophrenia Research, 143*(2-3), 319-326. http://dx.doi.org/10.1016/j.schres.2012.11.007

Royal College of Psychiatrists (2014). *Report of the second round of the National Audit of Schizophrenia (NAS2) 2014*. London, UK: Royal College of Psychiatrists Centre for Quality Improvement

Royal College of Psychiatrists (2018). *Racism and mental health: Position Statement 01/18*. London, UK: Royal College of Psychiatrists

Shih, R. A., Miles, J. N. V, Tucker, J. S., Zhou, A. J., & D'Amico, E. J. (2010). Racial/ethnic differences in adolescent substance use: mediation by individual, family, and school factors. Journal of Studies on Alcohol and Drugs, 71(5), 640–651.

Singh, S. P., Burns, T., Tyrer, P., Islam, Z., Parsons, H., & Crawford, M. J. (2014). Ethnicity as a predictor of detention under the Mental Health Act. *Psychological Medicine*, *44*(5), 997–1004. https://doi.org/10.1017/S003329171300086X

Stewart, R., Soremekun, M., Perera, G., Broadbent, M., Callard, F., Denis, M., Hotopf, M., Thornicroft, G. & Lovestone, S. (2009). The South London and Maudsley NHS Foundation Trust Biomedical Research Centre (SLAM BRC) Case Register: Development and Descriptive Data. BMC Psychiatry. 9, 51.

For peer review only

Ethnicity and impact on the implementation of CBT

Sweeney, A., Fahmy, S., Nolan, F., Morant, N., Fox, Z., Lloyd-Evans, B., … Johnson, S. (2014). The relationship between therapeutic alliance and service user satisfaction in mental health inpatient wards and crisis house alternatives: a cross-sectional study. *PloS One*, *9*(7), e100153. https://doi.org/10.1371/journal.pone.0100153

Tait, L., Birchwood, M., & Petertrower. (2004). Adapting to the challenge of psychosis: Personal resilience and the use of sealing-over (avoidant) coping strategies. *British Journal of Psychiatry*, 185(5), 410-415. https://doi:10.1192/bjp.185.5.410

Veling, W., Selten, J.-P., Mackenbach, J. P., & Hoek, H. W. (2007). Symptoms at first contact for psychotic disorder: comparison between native Dutch and ethnic minorities. *Schizophrenia Research*, *95*(1–3), 30–38. https://doi.org/10.1016/j.schres.2007.06.024

Vinkers, D. J., de Beurs, E., Barendregt, M., Rinne, T., & Hoek, H. W. (2010). Pre-trial psychiatric evaluations and ethnicity in the Netherlands. *International Journal of Law and Psychiatry*, *33*(3), 192–196. https://doi.org/10.1016/j.ijlp.2010.03.010

Walkup, J., Wei, W., Sambamoorthi, U., Crystal, S., & Yanos, P. T. (2006). Provision of Psychotherapy for a Statewide Population of Medicaid Beneficiaries With Schizophrenia. *Psychological Services*, *3*(4), 227–238. https://doi.org/10.1037/1541-1559.3.4.227

Wessely (2017, Oct). Mental Health Act: independent review. Retrieved on March 26, 2018, from https://www.gov.uk/government/publications/mental-health-act-independent-review/terms-of-reference-independent-review-of-the-mental-health-act-1983

For peer review only

Woodhead, C., Ashworth, M., Broadbent, M., Callard, F., Hotopf, M., Schofield, P., … Henderson, M. J. (2016). Cardiovascular disease treatment among patients with severe mental illness: a data linkage study between primary and secondary care. *The British Journal of General Practice*, *66*(647), e374–e381. https://doi.org/10.3399/bjgp16X685189

World Health Organisation (2010). *International Statistical Classification of Diseases and Related Health Problems 10th version*. Geneva, Switzerland: World Health Organisation. Retrieved on August 9, 2017, from http://apps.who.int/classifications/icd10/browse/2016/en

For peer review only

Tables

For peer review only

Table 1

Information on baseline demographics and diagnoses and their relevant proportions for each ethnic group

		White British		Irish		African		Caribbean		South Asian		Total		Chi² (DF)	*p* value
		N	%	N	%	N	%	N	%	N	%				
Ethnicity		10393	51.9	570	2.8	2817	14.1	5481	27.4	749	3.7	20010			
Female		5070	48.8	269	47.2	1350	47.9	2497	45.6	367	49	9553	47.7		
Male		5323	51.2	301	52.8	1467	52.1	2984	54.4	382	51	10457	52.3	15.6	<.01
Index of Multiple Deprivation**	1	1489	14.3	42	7.4	70	2.5	199	3.6	59	7.9	1859	9.3		
	2	1160	11.2	53	9.3	165	5.9	456	8.3	92	12.3	1926	9.6		
	3	1133	10.9	62	10.9	195	6.9	536	9.8	87	11.6	2013	10.1		
	4	1041	10.0	53	9.3	284	10.1	542	9.9	86	11.5	2006	10.0		
	5	980	9.4	58	10.2	302	10.7	584	10.7	82	11.0	2006	10.0	1000.0 (36)*	<.001
	6	920	8.9	62	10.9	327	11.6	654	11.9	69	9.2	2032	10.2		
	7	933	9.0	60	10.5	326	11.6	617	11.3	80	10.7	2016	10.1		
	8	919	8.8	59	10.4	407	14.5	651	11.9	54	7.2	2090	10.4		
	9	867	8.3	60	10.5	379	13.5	646	11.8	64	8.5	2016	10.1		
	10	951	9.2	61	10.7	362	12.9	596	10.9	76	10.2	2046	10.2		
Single marital status		8784	84.5	486	85.3	2300	81.7	5035	91.9	492	65.7	17097	85.4	456.4 (4)*	<.001

For peer review only

In relationship	1609	15.5	84	14.7	517	18.4	446	8.1	257	34.3	2913	14.6		
Age: median (IQR)	49 (26.9)		56 (28.8)		43 (18.8)		46 (22.3)		47 (26.2)		48 (24.5)		451.1 (4)*	<.001
Psychosis***	6516	62.7	366	64.2	2435	86.4	4617	84.2	563	75.2	14497	72.5	1200.0 (4) *	<.001
Bipolar****	3877	37.3	204	35.8	382	13.6	864	15.8	186	24.8	5513	27.6		
Lifetime Comorbid substance use diagnosis	1675	16.1	140	24.6	292	10.4	865	15.8	53	7.1	3025	15.1	94.4 (4) *	<.001

*Kruskal-Wallis H non parametric test for ranked data used to determine the Chi^2 value ; **1= least deprived,10= most deprived; ***Psychosis= diagnosis of schizophrenia, delusional disorder, brief psychotic disorder, shared psychotic disorder, schizoaffective disorder, Other nonorganic psychotic disorders, or Unspecified nonorganic psychosis; ****Bipolar= diagnosis of a Manic episode or Bipolar affective disorder.

For peer review only

For peer review only

Ethnicity and impact on the implementation of CBT

Table 2
Crude and adjusted associations from logistic regression models for at least one recorded session of CBT (inpatient or outpatient)

Variable	N	Odds Ratio (95% Confidence Interval)		
		Crude Associations	Step 1	Step 2
**Ethnicity**				
White British	10393	Reference group		
Irish	570	1.00 (0.82-1.21)	1.12 (0.91-1.36)	1.05 (0.85-1.29)
Black African	2817	1.06 (0.97-1.17)	0.96 (0.87-1.06)	0.73 (0.66-0.82) ***
Black Caribbean	5481	1.29 (1.20-1.39) ***	1.20 (1.11-1.30) ***	0.93 (0.86-1.02)
South Asian	749	0.99 (0.83-1.18)	0.97 (0.82-1.16)	0.93 (0.77-1.12)
**Gender**				
Female	9553	Reference group		
Male	10457	0.89 (0.84-0.95) ***	0.84 (0.78-0.89) ***	0.84 (0.78-0.90) ***
**Age** (years)		0.98 (0.98-0.99) ***	0.98 (0.98-0.99) ***	0.99 (0.98-0.99) ***
**Area level deprivation**				
IMD decile (per tenth)		1.01 (1.00-1.02)	1.01 (0.99-1.02)	0.99 (0.98-1.00)
**Marital status**				
In relationship	2913	Reference group		
Single	17097	1.23 (1.12-1.35)	1.08 (0.98-1.19)	1.07 (0.97-1.18)
**Diagnosis**				
Psychosis	14497	Reference Group		
Bipolar affective disorder	5513	0.94 (0.88-1.01)	0.93 (0.86-1.00)	1.00 (0.93-1.09)
**Comorbid substance misuse**				
No previous substance misuse diagnosis	16985	Reference group		
Lifetime comorbid substance misuse diagnosis	3025	1.31 (1.20-1.42) ***		0.85 (0.77-0.93)**
**Admission**				
No previous admission	10593	Reference Group		
Inpatient Admission Ever	9417	3.20 (2.99-3.42) ***		1.76 (1.58-1.95) ***
**Treatment under the Mental Health Act**				
Never treated under Mental Health Act	12904	Reference Group		
Ever treated under Mental Health Act	7106	2.54 (2.38-2.71) ***		0.96 (0.87-1.07)
**Structured risk assessment items#**				
History of Violence	6216	2.31 (2.16-2.47) ***		1.09 (1.00-1.20)
Difficulty managing physical health	3622	1.74 (1.61-1.88) ***		0.97 (0.88-1.07)
History of Non-adherence	6425	2.55 (2.39-2.73) ***		1.27 (1.16-1.39) ***
History of Suicide Attempt	3758	2.83 (2.63-3.05) ***		1.36 (1.22-1.53) ***
Lethal means used in suicide attempt	2026	2.65 (2.41-2.91) ***		1.04 (1.22-1.53) ***

For peer review only

Plans to end life	863	2.62 (2.29-3.01) ***	0.82 (0.69-0.96) *
Suicidal ideation	2041	3.23 (2.94-3.55) ***	1.24 (1.10-1.41) **
Feelings of hopelessness	2850	3.06 (2.82-3.32) ***	1.24 (1.11-1.40) ***
High level of distress	4666	3.24 (3.02-3.47) ***	1.53 (1.40-1.68) ***
No feelings of control	2972	3.03 (2.79-3.28) ***	1.22 (1.09-1.36) ***
**Referred/seen by other team**			
Never referred to Crisis team	13504	Reference Group	
Ever referred to the Crisis team	6506	2.96 (2.77-3.16) ***	1.69 (1.57-1.83) ***
Never seen at A & E~	13389	Reference Group	
Ever seen at A & E~	6621	1.69 (1.58-1.80) ***	0.97 (0.90-1.04)
Never referred to Assertive Outreach	18977	Reference Group	
Ever referred to Assertive Outreach	1033	1.51 (1.32-1.72) ***	0.94 (0.81-1.09)
**Forensic History**			
No forensic history reported	18137	Reference Group	
Forensic History reported	1873	1.70 (1.53-1.88) ***	1.07 (0.96-1.20)

**p<.05; **p<.01; ***p<.001*; IMD= Index of Multiple Deprivation, 1=least deprived, 10=most deprived; ~ Seen at A & E due to mental health emergency; # For brevity reference groups are omitted. Reference groups are a non-affirmative response to the item. The *n* for the reference group is the number of people included in the analysis (N=20010) – the number of people with an affirmative response.

Step 1 Adjusted for Ethnicity + Gender + Age + IMD decile + Marital Status + diagnosis: psychosis/bipolar

Step 2 Adjusted for Ethnicity + Gender + Age + IMD decile + Marital Status + diagnosis: psychosis/bipolar + Substance use diagnosis + inpatient admittance + treated under the MHA + Structured risk assessment items (entered separately) + Referred to crisis team + Treated at A & E + Referred to assertive outreach + forensic history

For peer review only

Ethnicity and impact on the implementation of CBT

Table 3
Crude and adjusted associations from logistic regression models for at least sixteen recorded sessions of CBT

Variable	N	Odds Ratio (95% Confidence Interval) Crude Associations	Step 1	Step 2
**Ethnicity**				
White British	2456	Reference group		
Irish	137	1.03 (0.70-1.50)	1.02 (0.70-1.50)	1.05 (0.71-1.55)
Black African	682	0.78 (0.64-0.95) *	0.77 (0.63-0.95) *	0.86 (0.69-1.06)
Black Caribbean	1524	0.77 (0.67-0.90) **	0.76 (0.65-0.89) **	0.83 (0.71-0.98) *
South Asian	178	0.98 (0.70-1.38)	0.99 (0.72-1.39)	1.03 (0.73-1.47)
**Gender**				
Female	2485	Reference group		
Male	2492	0.99 (0.87-1.12)	0.98 (0.86-1.11)	1.05 (0.91-1.20)
**Age** (years)		1.00 (1.00-1.01)	1.00 (1.00-1.01)	1.00 (1.00-1.01)
**Area level deprivation**				
IMD decile (per tenth)		0.99 (0.97-1.01)	1.00 (0.97 1.02)	0.99 (0.97-1.01)
**Marital Status**				
In relationship	639	Reference group		
Single	4338	1.07 (0.88-1.29)	1.11 (0.91-1.36)	1.21 (0.98-1.48)
**Diagnosis**				
Psychosis	3645	Reference group		
Bipolar affective disorder	1332	0.95 (0.83-1.10)	0.90 (0.77-1.04)	0.86 (0.74-1.01)
**Comorbid substance misuse**				
No previous substance misuse diagnosis	4090	Reference group		
Lifetime comorbid substance misuse diagnosis	887	0.81 (0.69-0.97) *		0.79 (0.66-0.96) *
**Admission**				
No previous admission	1622	Reference Group		
Inpatient Admission ever	3355	0.74 (0.65-0.85) ***		1.06 (0.86-1.31)
**Treatment under Mental Health Act**				
Never treated under Mental Health Act	2429	Reference Group		
Ever treated under the Mental Health Act	2548	0.70 (0.61-0.79) ***		0.86 (0.71-1.05)
**Structured risk assessment items#**				
History of Violence	2234	0.80 (0.71-0.91) **		0.93 (0.78-1.10)
Difficulty managing physical health	1237	0.94 (0.81-1.09)		1.01 (0.85-1.20)
History of non-adherence	2382	0.83 (0.73-0.95) **		0.91 (0.77-1.08)
History of Suicide Attempt	1589	1.39 (1.22-1.59) ***		1.33 (1.09-1.61) **
Lethal means used in	887	1.36 (1.16-1.60) ***		1.01 (0.80-1.27)

For peer review only

suicide attempt			
Reported plans to end life	382	1.54 (1.23-1.92) ***	1.33 (1.01-1.73) *
Suicidal ideation	961	1.38 (1.18-1.61) ***	1.10 (0.89-1.35)
Feelings of hopelessness	1287	1.32 (1.14-1.52) ***	1.01 (0.82-1.23)
High level of distress	2000	1.22 (1.07-1.39) **	1.22 (1.03-1.44) *
No feelings of control	1337	1.24 (1.08-1.43) **	1.09 (0.90-1.31)
**Referred/seen by other team**			
Never referred to Crisis team	2459	Reference Group	
Ever referred to the Crisis team	2518	1.27 (1.12-1.44) ***	1.34 (1.14-1.56) ***
Never seen at A & E~	2918	Reference Group	
Ever seen at A & E~	2059	0.96 (0.84-1.09)	0.93 (0.80-1.08)
Never referred to Assertive Outreach	4636	Reference Group	
Ever referred to Assertive Outreach	341	0.67 (0.51-0.89) **	0.81 (0.60-1.08)
**Forensic History**			
No forensic history reported	4326	Reference Group	
Forensic History reported	651	0.80 (0.66-0.98) *	0.86 (0.69-1.06)
**Context of first CBT session**			
First CBT as outpatient	3493	Reference Group	
First CBT as inpatient	1484	0.35 (0.29-0.41) ***	0.35 (0.29-0.42) ***

*p<.05;**p<.01;***p<.001;* IMD= Index of Multiple Deprivation, 1=least deprived, 10=most deprived ; ~ Seen at A & E due to mental health emergency; # For brevity reference groups are omitted. Reference groups are a non-affirmative response to the item. The *n* for the reference group is the number of people included in the analysis (N=4977) – the number of people with an affirmative response.

Step 1 Adjusted for Ethnicity + Gender + Age + IMD decile + Marital Status + diagnosis: psychosis/bipolar

Step 2 Adjusted for Ethnicity + Gender + Age + IMD decile + Marital Status + diagnosis: psychosis/bipolar + Substance use diagnosis + inpatient admittance + treated under the MHA + Structured risk assessment items (entered separately) + Referred to crisis team + Treated at A & E + Referred to assertive outreach + forensic history + First CBT as inpatient

For peer review only

Ethnicity and impact on the implementation of CBT

Table 4

Crude and adjusted associations from logistic regression models for at least one recorded session of CBT as an inpatient

Variable	N	Odds Ratio (95% Confidence Interval)		
		Crude Associations	Step 1	Step 2
**Ethnicity**				
White British	4000	Reference Group		
Irish	232	0.95 (0.69-1.32)	1.02 (0.73-1.41)	0.99 (0.71-1.39)
Black African	1734	0.82 (0.71-0.95) **	0.80 (0.69-0.93) **	0.76 (0.65-0.89) **
Black Caribbean	3132	0.93 (0.83-1.05)	0.91 (0.80-1.02)	0.83 (0.73-0.94) **
South Asian	319	0.82 (0.62-1.10)	0.83 (0.62-1.12)	0.86 (0.64-1.16)
**Gender**				
Female	4390	Reference group		
Male	5027	0.93 (0.84-1.03)	0.89 (0.80-0.99) *	0.87 (0.79-0.97) *
**Age (years)**		0.99 (0.99-1.00) ***	0.99 (0.99-1.00) ***	0.99 (0.99-0.99) ***
**Area level deprivation**				
IMD decile (per tenth)		0.97 (0.95-0.99) **	0.97 (0.96-0.99) **	0.97 (0.95-0.99) **
**Marital Status**				
In relationship	1234	Reference group		
Single	8183	1.24 (1.06-1.45) **	1.19 (1.02-1.40) *	1.08 (0.91-1.27)
**Diagnosis**				
Psychosis	7114	Reference group		
Bipolar affective disorder	2303	0.97 (0.86-1.09)	0.94 (0.83-1.06)	1.02 (0.90-1.16)
**Comorbid substance misuse**				
No previous substance misuse diagnosis	7456	Reference group		
Lifetime comorbid substance misuse diagnosis	1961	1.05 (0.93-1.19)		0.88 (0.77-1.00)
**Treatment under Mental Health Act**				
No treatment under Mental Health Act	2506	Reference Group		
Ever treated under Mental Health Act	6911	1.56 (1.38-1.76) ***		1.39 (1.21-1.59) ***
**Structured risk assessment items#**				
History of Violence	4914	1.56 (1.41-1.73) ***		1.13 (1.00-1.28) *
Difficulty managing physical health	2720	1.59 (1.44-1.77) ***		1.34 (1.19-1.51) ***
History of Non-adherence	5161	1.66 (1.50-1.84) ***		1.24 (1.09-1.41) **
History of Suicide Attempt	2879	1.61 (1.46-1.79) ***		1.17 (1.00-1.35) *
Lethal means used in suicide attempt	1612	1.56 (1.38-1.77) ***		1.02 (0.86-1.20)
Plans to end life	754	1.66 (1.41-1.96) ***		1.09 (0.89-1.32)
Suicidal ideation	1684	1.66 (1.47-1.87) ***		1.14 (0.97-1.33)
Feelings of hopelessness	2218	1.66 (1.48-1.85) ***		1.08 (0.93-1.25)

For peer review only

Ethnicity and impact on the implementation of CBT

High level of distress	3747	1.82 (1.65-2.02) ***	1.37 (1.22-1.54) ***
No feelings of control	2370	1.68 (1.51-1.87) ***	1.08 (0.94-1.24)
**Referred/seen by other team**			
Never referred to Crisis team	4217	Reference Group	
Ever referred to the Crisis team	5200	1.08 (0.97-1.19)	0.90 (0.80-1.00) *
Never seen at A & E~	4981	Reference Group	
Ever seen at A & E~	4436	1.22 (1.10-1.34) ***	1.11 (1.00-1.23)
Never referred to Assertive Outreach	8633	Reference Group	
Ever referred to Assertive Outreach	784	1.45 (1.23-1.71) ***	1.18 (0.99-1.41)
**Forensic History**			
No forensic history reported	7936	Reference Group	
Forensic History reported	1481	1.11 (0.97-1.27)	1.02 (0.89-1.18)

p*<.05;*p*<.01;****p*<.001; IMD= Index of Multiple Deprivation, 1=least deprived, 10=most deprived; ~ Seen at A & E due to mental health emergency; # For brevity reference groups are omitted. Reference groups are a non-affirmative response to the item. The *n* for the reference group is the number of people included in the analysis (N=9417) – the number of people with an affirmative response.

Step 1 Adjusted for Ethnicity + Gender + Age + IMD decile + Marital Status + diagnosis: psychosis/bipolar

Step 2 Adjusted for Ethnicity + Gender + Age + IMD decile + Marital Status + diagnosis: psychosis/bipolar + Substance use diagnosis + treated under the MHA + Structured risk assessment items (entered separately) + Referred to crisis team + Treated at A & E + Referred to assertive outreach + forensic history

For peer review only

Ethnicity and impact on the implementation of CBT

For peer review only

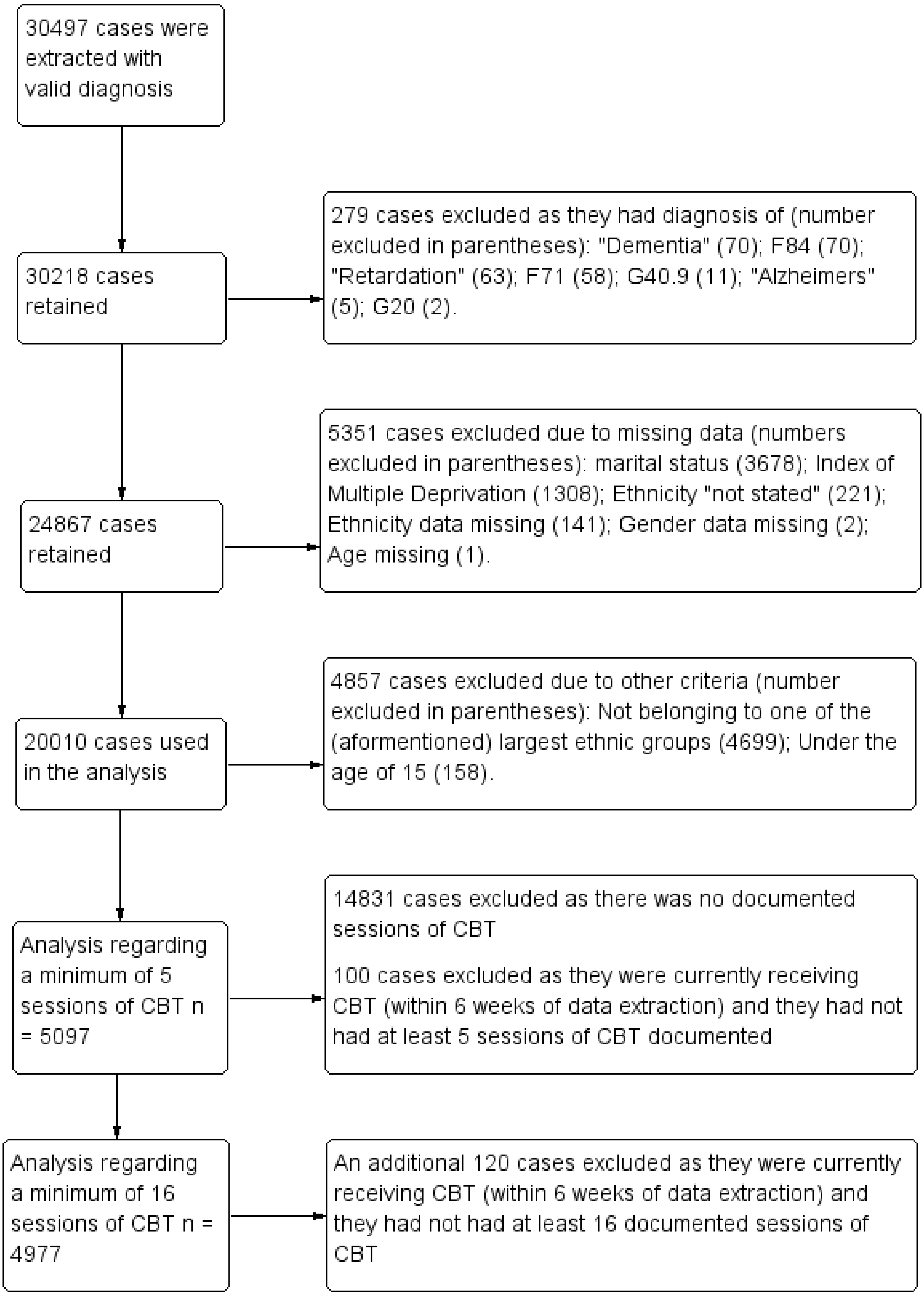

Figure 1. Demonstrating the flow of included cases

Supplementary Data Contents

Supplementary Data 1

Supplementary Data 1 contains further information regarding treatment, risk, and severity including items from a structured risk assessment. The table also contains tests of difference between the different ethnic groups.

Supplementary Data 2

Supplementary Data 2 contains information about CBT treatment received. The table displays the relevant proportions for each ethnic group and tests of difference.

Supplementary Data 3

Supplementary Data 3 contains crude estimates and adjusted multivariable logistic regression models regarding ethnicity and having a minimum of 5 sessions of CBT.

Supplementary Data 4

Supplementary Data 4 contains crude estimates and adjusted multivariable logistic regression models regarding ethnicity and reported receipt of CBT as an outpatient.

Supplementary Data 5

Supplementary Data 5 contains crude estimates and adjusted multivariable logistic regression models regarding ethnicity and reported receipt of CBT in the unstructured clinical notes (i.e. just using data derived from free text not structured fields).

.

For peer review only

Supplementary Data 6

Supplementary Data 6 contains crude estimates and adjusted multivariable logistic regression models regarding ethnicity and reported receipt of CBT which are adjusted for the effect of time.

For peer review only

Supplementary Data 1

Table 1
Information about clients' treatment and risk assessment

		White British N	White British %	Irish N	Irish %	African N	African %	Caribbean n	Caribbean %	South Asian n	South Asian %	Total N	Total %	Chi²*	*p*-value
	Ever treated under Mental Health Act	2575	24.8	155	27.2	1492	53.0	2648	48.3	236	31.5	7106	35.5	1300.0	<.001
	Inpatient admission ever	4000	38.5	232	40.7	1734	61.6	3132	57.1	319	42.6	9417	47.1	783.0	<.001
	Forensic History#	836	8.0	44	7.7	260	9.2	700	12.8	33	4.4	1873	9.4	119.9	<.001
Structured risk assessment items	History of violence	2324	22.4	140	24.6	1086	38.6	2491	45.4	175	23.4	6216	31.1	1000.0	<.001
	Difficulty managing physical health	1645	15.8	98	17.2	513	18.2	1240	22.6	126	16.8	3622	18.1	113.0	<.001
	History of non-adherence	2320	22.3	150	26.3	1177	41.8	2579	47.1	199	26.6	6425	32.1	1200.0	<.001
	History of suicide attempt	2062	19.8	119	20.9	445	15.8	1045	19.1	87	11.6	3758	18.8	51.2	<.001
	Lethal means used in suicide attempt	1157	56.1	67	56.3	230	51.7	531	50.8	41	47.1	2026	53.9	44.1	<.001

For peer review only

Current plans to end life	524	5.0	25	4.4	109	3.9	186	3.4	19	2.5	863	4.3	31.7	<*.001*
Current suicidal ideation	1126	10.8	73	12.8	256	9.1	526	9.6	60	8.0	2041	10.2	18.7	<*.01*
Hopelessness	1481	14.3	93	16.3	398	14.1	797	14.5	81	10.8	2850	14.2	9.6	*.047*
High levels of distress	1986	19.1	129	22.6	808	28.7	1595	29.1	148	19.8	4666	23.3	256.3	<*.001*
No perception of control	1404	13.5	86	15.1	442	15.7	950	17.3	90	12.0	2972	14.9	47.8	<*.001*
Crisis team ever	2642	25.4	145	25.4	1241	44.1	2209	40.3	269	35.9	6506	32.5	577.8	<*.001*
Assertive outreach ever	496	4.8	10	1.8	149	5.3	348	6.3	30	4.0	1033	5.2	34.7	<*.001*
A&E ever~	3172	30.5	191	33.5	1125	39.9	1879	34.3	254	33.9	6621	33.1	94.4	<*.001*

Forensic history noted in the clinical records includes self-reported and reports by professionals from different organisations; ~ Seen at A & E due to mental health; * All degrees of freedom = 4

Supplementary Data 2

Table 2

Information about CBT and the relevant proportions by ethnicity

	White British		Irish		African		Caribbean		South Asian		Total		Kruskal -Wallis *H*, Chi^2 *	*p*-value
	N	%	N	%	n	%	n	%	n	%	N	%		
CBT ever	2541	24.5	139	24.4	722	25.6	1613	29.4	182	24.3	5197	26.0	48.6	<.001
CBT on-going	160	6.3	9	6.5	66	9.1	141	8.7	10	5.5	386	7.4	24	<.001
Sessions: median (IQR)#	6 (14)		8 (15)		4 (12)		5 (11)		6 (14)		5 (13)		37.2	<.001
CBT inpatient ever	833	8.0	47	8.3	318	11.3	644	11.8	57	7.6	1899	9.5	47.1	<.001
Inpatient sessions: median (IQR) ##	2 (5)		2 (3)		2 (2)		2 (3)		1 (3)		2 (3)		22.4	<.001
CBT outpatient ever	2011	19.4	113	19.8	514	18.3	1197	21.8	147	19.6	3982	19.9	14.2	<.01
Outpatient sessions: median (IQR) ###	8 (16)		8 (16)		6 (15)		6 (14)		8 (16)		7 (15)		38.1	<.001
% of sessions Face to Face	96.5		97.8		97.0		96.6		98.3		96.7			

For peer review only

% of sessions one to one	94.7		91.7		92.4		93.1		96.5		93.9			
1st CBT as inpatient##	670	26.4	37	26.6	262	36.3	537	33.3	51	28.0	1557	30.0	68.2	<.001
>= 5 sessions~	1477	58.9	82	59.4	344	48.9	818	52.3	101	56.1	2822	55.4	31.4	<.001
>= 16 sessions~	686	27.9	39	28.7	158	23.2	352	23.1	49	27.5	1284	25.8	14.9	<.01

* Kruskal-Wallis H non parametric test for ranked data used to determine the Chi^2 value , all degrees of freedom = 4; # Including only the people who had received CBT; ## Including only people who have received CBT as an inpatient; ### Including only people who have received CBT as an outpatient

~ Only includes people who had ever received CBT and people who had not received CBT within 6 weeks of data extraction if they did not meet the criteria (i.e. people with <5 or <16 sessions who were currently receiving CBT)

For peer review only

Supplementary Data 3

Table 3

Crude and adjusted associations from logistic regression models for at least five recorded sessions of CBT (inpatient or outpatient)

Variable	N	Odds Ratio (95% Confidence Interval)		
		Crude Associations	Step 1	Step 2
**Ethnicity**				
White British	2509	Reference group		
Irish	139	1.01 (0.71-1.42)	1.02 (0.72-1.44)	1.02 (0.71-1.47)
Black African	704	0.67 (0.56-0.79) ***	0.68 (0.57-0.81) ***	0.76 (0.63-0.91) **
Black Caribbean	1565	0.77 (0.67-0.89) ***	0.79 (0.69-0.90) ***	0.88 (0.76-1.01)
South Asian	180	0.89 (0.66-1.21) ***	0.88 (0.64-1.19)	0.91 (0.66-1.25)
**Gender**				
Female	2543	Reference Group		
Male	2554	0.97 (0.87-1.09) ***	0.99 (0.88-1.11)	1.09 (0.96-1.23)
**Age (years)**		1.00 (1.00-1.00)	1.00 (1.00-1.00)	1.00 (1.00-1.00)
**Area level deprivation**				
IMD decile (per tenth)		0.97 (0.96-0.99) *	0.99 (0.97-1.01)	0.98 (0.96-1.00)*
**Marital Status**				
In relationship	654	Reference Group		
Single	4443	0.84 (0.71-0.99) *	0.87 (0.73-1.03)	0.95 (0.79-1.14)
**Diagnosis**				
Psychosis	3734	Reference Group		
Bipolar affective disorder	1363	1.14 (1.01-1.29) *	1.04 (0.92-1.19)	1.00 (0.87-1.15)
**Comorbid substance misuse**				
No previous substance misuse diagnosis	4191	Reference group		
Lifetime comorbid substance misuse diagnosis	906	0.78 (0.68-0.90) **		0.79 (0.68-0.93) **
**Admission**				
No previous admission	1650	Reference Group		
Inpatient admission ever	3447	0.64 (0.57-0.72) ***		1.05 (0.87-1.28)
**Treatment under Mental Health Act**				
No treatment under Mental Health Act	2473	Reference Group		
Ever treated under Mental Health Act	2624	0.63 (0.56-0.70) ***		0.91 (0.76-1.08)**
**Structured risk assessment items#**				
History of Violence	2288	0.71 (0.64-0.80) ***		0.88 (0.76-1.02)
Difficulty managing physical health	1257	0.81 (0.71-0.92) **		0.96 (0.83-1.12)
History of Non-adherence	2443	0.73 (0.66-0.82) ***		0.90 (0.77-1.05)

For peer review only

History of Suicide Attempt	1628	1.21 (1.08-1.37) **	1.23 (1.03-1.72) *
Lethal means used in suicide attempt	904	1.20 (1.04-1.39) *	0.98 (0.80-1.21)
Plans to end life	396	1.43 (1.16-1.77) **	1.33 (1.03-1.72) *
Suicidal ideation	991	1.30 (1.13-1.50) ***	1.13 (0.93-1.36)
Feelings of hopelessness	1318	1.20 (1.05-1.36) **	0.97 (0.81-1.16)
High level of distress	2059	1.13 (1.01-1.26) *	1.29 (1.11-1.49) **
No feelings of control	1366	1.12 (0.99-1.27)	1.06 (0.89-1.25)
**Referred/seen by other team**			
Never referred to Crisis team	2508	Reference Group	
Ever referred to the Crisis team	2589	1.13 (1.01-1.26) *	1.21 (1.05-1.38) **
Never seen at A & E~	2971	Reference Group	
Ever seen at A & E~	2126	0.96 (0.86-1.07)	1.00 (0.88-1.14)
Never referred to Assertive Outreach	4752	Reference Group	
Ever referred to Assertive Outreach	345	0.65 (0.52-0.80) ***	0.83 (0.65-1.05)
**Forensic History**			
No forensic history reported	4435	Reference Group	
Forensic History reported	662	0.72 (0.61-0.85) ***	0.81 (0.68-0.97) *
**Context of first CBT session**			
First CBT as outpatient	3584	Reference Group	
First CBT as inpatient	1513	0.33 (0.29-0.38) ***	0.34 (0.29-0.39) ***

p<.05;p<.01;***p<.001*; IMD= Index of Multiple Deprivation, 1=least deprived, 10=most deprived; ~ Seen at A & E due to mental health emergency; # For brevity reference groups are omitted. Reference groups are a non-affirmative response to the item. The *n* for the reference group is the number of people included in the analysis (N=5097) – the number of people with an affirmative response.

Step 1 Adjusted for Ethnicity + Gender + Age + IMD decile + Marital Status + diagnosis: psychosis/bipolar

Step 2 Adjusted for Ethnicity + Gender + Age + IMD decile + Marital Status + diagnosis: psychosis/bipolar + Substance use diagnosis + inpatient admittance + treated under the MHA + Structured risk assessment items (entered separately) + Referred to crisis team + Treated at A & E + Referred to assertive outreach + forensic history + First CBT as inpatient

For peer review only

Supplementary Data 4

Table 4

Crude and adjusted associations from logistic regression models for at least one recorded session of CBT as an outpatient

Variable	N	Odds Ratio (95% Confidence Interval) Crude Associations	Step 1	Step 2
**Ethnicity**				
White British	10393	Reference Group		
Irish	570	1.03 (0.84-1.27)	1.15 (0.94- 1.42)	1.10 (0.89- 1.37)
Black African	2817	0.90 (0.81-1.00)	0.83 (0.74 - 0.92) **	0.75 (0.67-0.84) ***
Black Caribbean	5481	1.11 (1.03-1.20) **	1.06 (0.98- 1.15)	0.95 (0.87- 1.04)
South Asian	749	1.00 (0.83-1.20)	0.98 (0.81-1.18)	0.97 (0.80-1.17)
**Gender**				
Female	9553	Reference Group		
Male	10457	0.85 (0.80-0.92) ***	0.82 (0.76-0.88) ***	0.85 (0.79-0.91) ***
**Age (years)**		0.99 (0.98-0.99) ***	0.98 (0.98-0.99) ***	0.99 (0.98-0.99) ***
**Area level deprivation**				
IMD decile (per tenth)		1.00 (0.99-1.01)	1.00 (0.99-1.02)	0.99 (0.98-1.00)
**Marital Status**				
In relationship	2913	Reference Group		
Single	17097	1.09 (0.98-1.20)	0.98 (0.88-1.08)	1.01 (0.91-1.13)
**Diagnosis**				
Psychosis	14497	Reference Group		
Bipolar	5513	1.09 (1.01-1.18) *	1.15 (0.94- 1.42)	1.07 (0.99-1.16)
**Comorbid substance misuse**				
No previous substance misuse diagnosis	16985	Reference group		
Lifetime comorbid substance misuse diagnosis	3025	1.15 (1.05-1.27) **		0.86 (0.78-0.95) **
**Admission**				
No previous admission	10593	Reference Group		
Inpatient Admission Ever	9417	1.69 (1.57-1.81) ***		1.13 (1.01-1.27) *
**Treatment under Mental Health Act#**				
No treatment under Mental Health Act	12904	Reference Group		
Ever treated under Mental Health Act	7106	1.40 (1.30-1.50) ***		0.75 (0.67-0.84) ***
**Structured risk assessment items#**				
History of Violence	6216	1.54 (1.43-1.65) ***		1.02 (0.93-1.13)
Difficulty managing physical health	3622	1.19 (1.09-1.29) ***		0.83 (0.75-0.92) **
History of Non-adherence	6425	1.69 (1.57-1.81) ***		1.22 (1.11-1.35) ***
History of Suicide Attempt	3758	2.25 (2.08-2.44) ***		1.38 (1.23-1.56) ***

For peer review only

Lethal means used in suicide attempt	2026	2.09 (1.89-2.31) ***	1.02 (0.88-1.17)
Plans to end life	863	2.01 (1.74-2.33) ***	0.79 (0.66-0.94) **
Suicidal ideation	2041	2.62 (2.38-2.89) ***	1.26 (1.11-1.44) ***
Feelings of hopelessness	2850	2.50 (2.30-2.73) ***	1.30 (1.15-1.47) ***
High level of distress	4666	2.36 (2.19-2.54) ***	1.50 (1.36-1.65) ***
No feelings of control	2972	2.33 (2.14-2.54) ***	1.21 (1.08-1.36) **
**Referred/seen by other team**			
Never referred to Crisis team	13504	Reference Group	
Ever referred to the Crisis team	6506	2.49 (2.32-2.67)***	2.02 (1.86-2.21) ***
Never seen at A & E~	13389	Reference Group	
Ever seen at A & E~	6621	1.36 (1.27-1.46)***	0.88 (0.81-0.96) **
Never referred to Assertive Outreach	18977	Reference Group	
Ever referred to Assertive Outreach	1033	0.92 (0.78-1.07)	0.71 (0.60-0.84) ***
**Forensic History**			
No forensic history reported	18137	Reference Group	
Forensic History reported	1873	1.45 (1.30-1.61) ***	1.15 (1.02-1.29) *
**Context of first CBT session**			
First CBT not as inpatient	18453	Reference Group	
First CBT as inpatient	1557	1.08 (0.95-1.23)	0.71 (0.62-0.81) ***

p<.05;p<.01;***p<.001;* IMD= Index of Multiple Deprivation, 1=least deprived, 10=most deprived; ~ Seen at A & E due to mental health emergency; # For brevity reference groups are omitted. Reference groups are a non-affirmative response to the item. The *n* for the reference group is the number of people included in the analysis (N=20010) – the number of people with an affirmative response.

Step 1 Adjusted for Ethnicity + Gender + Age + IMD decile + Marital Status + diagnosis: psychosis/bipolar

Step 2 Adjusted for Ethnicity + Gender + Age + IMD decile + Marital Status + diagnosis: psychosis/bipolar + Substance use diagnosis + inpatient admittance + treated under the MHA + Structured risk assessment items (entered separately) + Referred to crisis team + Treated at A & E + Referred to assertive outreach + forensic history + First CBT as an inpatient

For peer review only

Supplementary Data 5

Sensitivity Analysis

Table 5
Crude and adjusted associations from logistic regression models for at least one recorded session of CBT (inpatient or outpatient) in the unstructured clinical notes

Variable	N	Odds Ratio (95% Confidence Interval)		
		Crude Associations	Step 1	Step 2
**Ethnicity**				
White British	10393	Reference group		
Irish	570	0.88 (0.62-1.26)	1.07 (0.75-1.53)	1.04 (0.72-1.49)
Black African	2817	0.85 (0.71-1.01)	0.79 (0.66-0.95)*	0.76 (0.63-0.92) **
Black Caribbean	5481	0.96 (0.85-1.10)	0.95 (0.82-1.09)	0.90 (0.78-1.04)
South Asian	749	0.72 (0.52-1.02)	0.70 (0.50-0.99)*	0.89 (0.49-0.98)
**Gender**				
Female	9553	Reference group		
Male	10457	0.84 (0.75-0.94) **	0.83 (0.74-0.93) **	0.89 (0.79-1.00) *
**Age (years)**		0.98 (0.97-0.98) ***	0.98 (0.97-0.98) ***	0.98 (0.97-0.98) ***
**Area level deprivation**				
IMD decile (per tenth)		0.98 (0.96-1.00)*	0.99 (0.97-1.01)	0.98 (0.96-1.00)
**Marital Status**				
In relationship	2913	Reference group		
Single	17097	1.02 (0.87-1.21)	0.88 (0.74-1.04)	0.91 (0.77-1.09)
**Diagnosis**				
Psychosis	14497	Reference Group		
Bipolar affective disorder	5513	1.27 (1.12-1.43)***	1.13 (0.99-1.29)	1.17 (1.03-1.34)*
**Comorbid substance misuse**				
No previous substance misuse diagnosis	16985	Reference group		
Lifetime comorbid substance misuse diagnosis	3025	0.96 (0.82-1.13)		0.75 (0.64-0.89)**
**Admission**				
No previous admission	10593	Reference Group		
Inpatient Admission Ever	9417	1.65 (1.47-1.85) ***		1.60 (1.35-1.90) ***
**Treatment under Mental Health Act**				
No treatment under Mental Health Act	12904	Reference Group		
Ever treated under Mental Health Act	7106	1.19 (1.06-1.33) **		0.67 (0.57-0.80)***
**Structured risk assessment items#**				
History of Violence	6216	1.16 (1.03-1.31) *		0.84 (0.72-0.99)*

For peer review only

Difficulty managing physical health	3622	1.01 (0.87-1.17)	0.92 (0.78-1.09)
History of Non-adherence	6425	1.29 (1.15-1.45) ***	1.12 (0.95-1.31)
History of Suicide Attempt	3758	1.91 (1.69-2.17) ***	1.23 (1.02-1.50) *
Lethal means used in suicide attempt	2026	2.00 (1.72-2.32) ***	1.20 (0.97-1.50)
Plans to end life	863	2.01 (1.62-2.50) ***	0.90 (0.69-1.16)
Suicidal ideation	2041	2.42 (2.09-2.79) ***	1.43 (1.17-1.74) ***
Feelings of hopelessness	2850	1.95 (1.71-2.24) ***	1.11 (0.91-1.35)
High level of distress	4666	1.90 (1.69-2.14) ***	1.43 (1.17-1.74) ***
No feelings of control	2972	1.76 (1.54-2.02) ***	1.02 (0.85-1.23)
**Referred/seen by other team**			
Never referred to Crisis team	13504	Reference Group	
Ever referred to the Crisis team	6506	1.80 (1.61-2.02) ***	1.45 (1.26-1.66) ***
Never seen at A & E~	13389	Reference Group	
Ever seen at A & E~	6621	1.16 (1.03-1.30) *	0.76 (0.67-0.87)***
Never referred to Assertive Outreach	18977	Reference Group	
Ever referred to Assertive Outreach	1033	0.75 (0.56-1.00) *	0.61 (0.46-0.82)**
**Forensic History**			
No forensic history reported	18137	Reference Group	
Forensic History reported	1873	1.11 (0.92-1.34)	0.96 (0.78-1.17)

**p<.05; **p<.01; ***p<.001*; IMD= Index of Multiple Deprivation, 1=least deprived, 10=most deprived; ~ Seen at A & E due to mental health emergency; # For brevity reference groups are omitted. Reference groups are a non-affirmative response to the item. The *n* for the reference group is the number of people included in the analysis (N=20010) – the number of people with an affirmative response.

Step 1 Adjusted for Ethnicity + Gender + Age + IMD decile + Marital Status + diagnosis: psychosis/bipolar

Step 2 Adjusted for Ethnicity + Gender + Age + IMD decile + Marital Status + diagnosis: psychosis/bipolar + Substance use diagnosis + inpatient admittance + treated under the MHA + Structured risk assessment items (entered separately) + Referred to crisis team + Treated at A & E + Referred to assertive outreach + forensic history

For peer review only

Supplementary Data 6

Time analysis

Table 6
Crude and adjusted associations from logistic regression models for at least one recorded session of CBT (inpatient or outpatient) adjusting for time

Variable	N	Odds Ratio (95% Confidence Interval)		
		Crude Associations#	Step 1	Step 2
**Ethnicity**				
White British	10393	Reference group		
Irish	570	0.97 (0.80-1.19)	1.10 (0.90-1.35)	1.04 (0.84-1.28)
Black African	2817	1.06 (0.96-1.17)	0.93 (0.84-1.03)	0.72 (0.65-0.81) ***
Black Caribbean	5481	1.27 (1.18-1.36) ***	1.16 (1.08-1.25) ***	0.92 (0.85-1.00)
South Asian	749	1.00 (0.84-1.19)	0.97 (0.82-1.16)	0.93 (0.77-1.12)
**Gender**				
Female	9553	Reference group		
Male	10457	0.88 (0.83-0.94)***	0.81 (0.76-0.87) ***	0.83 (0.78-0.89) ***
**Age (years)**		0.98 (0.98-0.98) ***	0.98 (0.98-0.98) ***	0.98 (0.98-0.99) ***
**Area level deprivation**				
IMD decile (per tenth)		1.01 (1.00-1.02)	1.01 (0.99-1.02)	0.99 (0.98-1.00)
**Marital Status**				
In relationship	2913	Reference group		
Single	17097	1.22 (1.11-1.34) ***	1.56 (0.95-1.15)	1.05 (0.95-1.17)
**Diagnosis**				
Psychosis	14497	Reference Group		
Bipolar affective disorder	5513	0.96 (0.89-1.03)	0.94 (0.87-1.01)	1.01 (0.93-1.09)
**Comorbid substance misuse**				
No previous substance misuse diagnosis	16985	Reference group		
Lifetime comorbid substance misuse diagnosis	3025	1.31 (1.20-1.43) ***		0.85 (0.77-0.94)**
**Admission**				
No previous admission	10593	Reference Group		
Inpatient Admission Ever	9417	3.16 (2.96-3.38) ***		1.74 (1.56-1.93) ***
**Treatment under Mental Health Act**				
No treatment under Mental Health Act	12904	Reference Group		
Ever treated under Mental Health Act	7106	2.51 (2.35-2.68) ***		0.98 (0.88-1.09)
**Structured risk assessment items#**				
History of Violence	6216	2.26 (2.12-2.42) ***		1.08 (0.99-1.19)

Difficulty managing physical health	3622	1.68 (1.55-1.81) ***		0.96 (0.87-1.05)
History of Non-adherence	6425	2.51 (2.35-2.68) ***		1.24 (1.13-1.36) ***
History of Suicide Attempt	3758	2.79 (2.59-3.01) ***		1.35 (1.21-1.52) ***
Lethal means used in suicide attempt	2026	2.61 (2.37-2.86) ***		1.04 (0.91-1.19)
Plans to end life	863	2.64 (2.30-3.03) ***		0.82 (0.70-0.97) *
Suicidal ideation	2041	3.26 (2.97-3.58) ***		1.26 (1.11-1.43) ***
Feelings of hopelessness	2850	3.04 (2.80-3.30) ***		1.25 (1.11-1.41) ***
High level of distress	4666	3.21 (2.99-3.44) ***		1.55 (1.41-1.69) ***
No feelings of control	2972	2.98 (2.75-3.23) ***		1.20 (1.08-1.34) **
**Referred/seen by other team**				
Never referred to Crisis team	13504	Reference Group		
Ever referred to the Crisis team	6506	2.93 (2.74-3.13) ***		1.67 (1.54-1.81) ***
Never seen at A & E~	13389	Reference Group		
Ever seen at A & E~	6621	1.74 (1.63-1.86) ***		0.99 (0.91-1.06)
Never referred to Assertive Outreach	18977	Reference Group		
Ever referred to Assertive Outreach	1033	1.46 (1.27-1.66) ***		0.92 (0.80-1.06)
**Forensic History**				
No forensic history reported	18137	Reference Group		
Forensic History reported	1873	1.62 (1.46-1.79) ***		1.02 (0.91-1.14)
**Time point diagnosis given##**				
Psychosis/bipolar affective disorder diagnosis before midpoint of study period	13518	Reference category		
Psychosis/bipolar affective disorder diagnosis after midpoint of study period	6492	0.76 (0.71-0.82) ***	0.64 (0.59-0.69) ***	0.77 (0.71-0.83) ***

**p<.05; **p<.01; ***p<.001*; # including time; IMD= Index of Multiple Deprivation, 1=least deprived, 10=most deprived; ~ Seen at A & E due to mental health emergency; # For brevity reference groups are omitted. Reference groups are a non-affirmative response to the item. The *n* for the reference group is the number of people included in the analysis (N=20010) – the number of people with an affirmative response.
##midpoint of study period was 16/4/2012
Step 1 Adjusted for Ethnicity + Gender + Age + IMD decile + Marital Status + diagnosis: psychosis/bipolar + Psychosis/bipolar affective disorder diagnosis after 16/04/12
Step 2 Adjusted for Ethnicity + Gender + Age + IMD decile + Marital Status + diagnosis: psychosis/bipolar + Substance use diagnosis + inpatient admittance + treated under the MHA + Structured risk assessment items (entered separately) + Referred to crisis team + Treated at A & E + Referred to assertive outreach + forensic history + Psychosis/bipolar affective disorder diagnosis after 16/04/12

For peer review only

For peer review only

STROBE Statement—Checklist of items that should be included in reports of cohort studies

**Title and abstract** 1	(a) Indicate the study's design with a commonly used term in the title or the abstract	X
	(b) Provide in the abstract an informative and balanced summary of what was done and what was found	X
**Introduction**		
Background/rationale 2	Explain the scientific background and rationale for the investigation being reported	X
Objectives 3	State specific objectives, including any prespecified hypotheses	X
**Methods**		
Study design 4	Present key elements of study design early in the paper	X
Setting 5	Describe the setting, locations, and relevant dates, including periods of recruitment, exposure, follow-up, and data collection	X
Participants 6	(a) Give the eligibility criteria, and the sources and methods of selection of participants. Describe methods of follow-up	X
	(b) For matched studies, give matching criteria and number of exposed and unexposed	n/a
Variables 7	Clearly define all outcomes, exposures, predictors, potential confounders, and effect modifiers. Give diagnostic criteria, if applicable	X
Data sources/ measurement 8	For each variable of interest, give sources of data and details of methods of assessment (measurement). Describe comparability of assessment methods if there is more than one group	X
Bias 9	Describe any efforts to address potential sources of bias	X
Study size 10	Explain how the study size was arrived at	X
Quantitative variables 11	Explain how quantitative variables were handled in the analyses. If applicable, describe which groupings were chosen and why	X
Statistical methods 12	(a) Describe all statistical methods, including those used to control for confounding	X
	(b) Describe any methods used to examine subgroups and interactions	X
	(c) Explain how missing data were addressed	X
	(d) If applicable, explain how loss to follow-up was addressed	n/a
	(e) Describe any sensitivity analyses	X
**Results**		
Participants 13	(a) Report numbers of individuals at each stage of study—eg numbers potentially eligible, examined for eligibility, confirmed eligible, included in the study, completing follow-up, and analysed	X
	(b) Give reasons for non-participation at each stage	X
	(c) Consider use of a flow diagram	X
Descriptive data 14	(a) Give characteristics of study participants (eg demographic, clinical, social) and information on exposures and potential confounders	X
	(b) Indicate number of participants with missing data for each variable of interest	n/a
	(c) Summarise follow-up time (eg, average and total amount)	n/a
Outcome data 15	Report numbers of outcome events or summary measures over time	X
Main results 16	(a) Give unadjusted estimates and, if applicable, confounder-adjusted	X

For peer review only

	estimates and their precision (eg, 95% confidence interval). Make clear which confounders were adjusted for and why they were included	
	(b) Report category boundaries when continuous variables were categorized	n/a
	(c) If relevant, consider translating estimates of relative risk into absolute risk for a meaningful time period	n/a
Other analyses 17	Report other analyses done—eg analyses of subgroups and interactions, and sensitivity analyses	X
**Discussion**		
Key results 18	Summarise key results with reference to study objectives	X
Limitations 19	Discuss limitations of the study, taking into account sources of potential bias or imprecision. Discuss both direction and magnitude of any potential bias	X
Interpretation 20	Give a cautious overall interpretation of results considering objectives, limitations, multiplicity of analyses, results from similar studies, and other relevant evidence	X
Generalisability 21	Discuss the generalisability (external validity) of the study results	X
**Other information**		
Funding 22	Give the source of funding and the role of the funders for the present study and, if applicable, for the original study on which the present article is based	X

For peer review only

BMJ Open

Ethnicity and impact on the receipt of Cognitive Behavioural Therapy in people with psychosis or bipolar disorder: An English cohort study

Journal:	*BMJ Open*
Manuscript ID	bmjopen-2019-034913.R1
Article Type:	Original research
Date Submitted by the Author:	08-Apr-2020
Complete List of Authors:	Morris, Rohan; Lancaster University, Division of Health Research; Lancashire Care NHS Foundation Trust<br>Sellwood, William; Lancaster University, Division of Health Research<br>Edge, Dawn; The University of Manchester, Division of Psychology & Mental Health, School of Health Sciences<br>Colling, Craig; King's College London, Institute of Psychiatry, Psychology and Neuroscience; South London and Maudsley NHS Foundation Trust<br>Stewart, Robert; King's College London, Institute of Psychiatry, Psychology and Neuroscience; South London and Maudsley NHS Foundation Trust<br>Cupitt, Caroline; South London and Maudsley NHS Foundation Trust<br>Das-Munshi, Jayati; King's College London, Institute of Psychiatry, Section of Epidemiology, Department of Health Service & Population Research
**Primary Subject Heading**:	Mental health
Secondary Subject Heading:	Mental health
Keywords:	Schizophrenia & psychotic disorders < PSYCHIATRY, Depression & mood disorders < PSYCHIATRY, MENTAL HEALTH, Adult psychiatry < PSYCHIATRY

SCHOLARONE™
Manuscripts

BMJ

I, the Submitting Author has the right to grant and does grant on behalf of all authors of the Work (as defined in the below author licence), an exclusive licence and/or a non-exclusive licence for contributions from authors who are: i) UK Crown employees; ii) where BMJ has agreed a CC-BY licence shall apply, and/or iii) in accordance with the terms applicable for US Federal Government officers or employees acting as part of their official duties; on a worldwide, perpetual, irrevocable, royalty-free basis to BMJ Publishing Group Ltd ("BMJ") its licensees and where the relevant Journal is co-owned by BMJ to the co-owners of the Journal, to publish the Work in this journal and any other BMJ products and to exploit all rights, as set out in our licence.

The Submitting Author accepts and understands that any supply made under these terms is made by BMJ to the Submitting Author unless you are acting as an employee on behalf of your employer or a postgraduate student of an affiliated institution which is paying any applicable article publishing charge ("APC") for Open Access articles. Where the Submitting Author wishes to make the Work available on an Open Access basis (and intends to pay the relevant APC), the terms of reuse of such Open Access shall be governed by a Creative Commons licence – details of these licences and which Creative Commons licence will apply to this Work are set out in our licence referred to above.

Other than as permitted in any relevant BMJ Author's Self Archiving Policies, I confirm this Work has not been accepted for publication elsewhere, is not being considered for publication elsewhere and does not duplicate material already published. I confirm all authors consent to publication of this Work and authorise the granting of this licence.

For peer review only

Ethnicity and impact on the receipt of Cognitive Behavioural Therapy in people with psychosis or bipolar disorder: An English cohort study

Rohan Morris[1,2] William Sellwood[1] Dawn Edge[3] Craig Colling[4,5] Robert Stewart[4,5] Caroline Cupitt[5] Jayati Das-Munshi[4,5]

[1] Division of Health Research, Lancaster University, UK

[2] Lancashire Care Foundation Trust, Lancashire, UK

[3]Division of Psychology & Mental Health, School of Health Sciences, University of Manchester

[4] Institute of Psychiatry, Psychology and Neuroscience, King's College London, London, United Kingdom

[5] South London & Maudsley NHS Foundation Trust, London, UK

Corresponding author contact details: Dr Rohan Michael Morris, r.morris1@lancaster.ac.uk, Lancaster University, Furness College, Department of Clinical Psychology, Division of Health Research, LA1 4YW, +44 (0)1524 592691

Keywords: Psychotic Disorders, Mood Disorders, Psychotherapy, Ethnic Groups, Cognitive Behavioral Therapy

Word count 4825

For peer review only

Ethnicity and impact on the receipt of CBT

For peer review only

For peer review only - http://bmjopen.bmj.com/site/about/guidelines.xhtml

Ethnicity and impact on the receipt of CBT

Abstract

Objectives:1) To explore the role of ethnicity in receiving Cognitive Behavioural Therapy (CBT) for people with psychosis or bipolar disorder whilst adjusting for differences in risk profiles and symptom severity. 2) To assess whether context of treatment (inpatient versus community) impacts on the relationship between ethnicity and access to CBT.

Design: Cohort study of case-register data from one catchment area (January 2007 to July 2017).

Setting: A large secondary care provider serving an ethnically-diverse population in London.

Participants: Data extracted for 30,497 records of people who had diagnoses of bipolar disorder (ICD Code F30-1) or psychosis (F20-F29 excluding F21). Exclusion criteria were: <15 years old, missing data, and not self-defining as belonging to one of the larger ethnic groups. The sample (N=20010) comprised the following ethnic groups: White British, n=10393; Black Caribbean, n=5481, Black African, n=2817; Irish, n= 570; and 'South Asian' people (consisting of Indian, Pakistani, and Bangladeshi people) n=749.

Outcome Assessments: Odds ratios for receipt of CBT (single session or full course) as determined via multivariable logistic regression analyses.

Results: In models adjusted for risk and severity variables, in comparison to White British people; Black African people were less likely to receive a single session of CBT (OR 0.73, CI 0.66 to 0.82, p<.001); Black Caribbean people were less likely to receive a minimum of 16-sessions of CBT (OR 0.83, CI 0.71 to 0.98, p=.03); Black African and Black Caribbean people were significantly less likely to receive CBT whilst inpatients (respectively OR 0.76, CI 0.65 to 0.89, p=.001; OR 0.83, CI 0.73 to 0.94, p=.003).

For peer review only - http://bmjopen.bmj.com/site/about/guidelines.xhtml

Ethnicity and impact on the receipt of CBT

Conclusions: This study highlights disparity in receipt of CBT from a large provider of secondary care in London for Black African and Caribbean people and that the context of therapy (inpatient versus community settings) has a relationship with disparity in access to treatment.

Strengths and Limitations

- A key strength of this study is that the data were from a near-complete case register of a large secondary care mental health service provider; which has a near monopoly on mental health provision in its catchment area.
- Published data are available on the tools used for extracting information about CBT which indicates high degrees of precision (95%) and sensitivity (96%).
- A limitation of this study is that it was not possible to assess access to other types of psychological intervention (e.g. Family Therapy).
- This study was not able to assess the offer of therapy (only receipt), consequently it is unclear if there are ethnic differences in whether or not therapy is offered to Black service users.

For peer review only

Ethnicity and impact on the receipt of CBT

Introduction

Background

There are ethnic differences in the care pathways and treatments people with psychosis receive. Within the UK, people of Black Caribbean and Black African descent are more likely to: enter mental health services via forensic pathways and experience compulsory detention,[1] receive medication by depot,[2] and be subject to Community Treatment Orders (CTO).[3] Black people with treatment resistant schizophrenia are less likely to receive drug treatments in accordance with national guidelines and Asian British people with a schizophrenia diagnosis are less likely to receive copies of their care plans.[2] Treatment inequalities based on ethnicity have also been identified in other countries. For example, in the USA, people of African descent have less money spent on their healthcare through state funded programs[4] and are less likely to receive medication associated with fewer side effects.[5] In the Netherlands, ethnic minority groups are more likely to be compulsorily detained for treatment and less likely to be recommended for outpatient treatment.[6]

A prospective study in the UK, found significant ethnic differences in Mental Health Act 2007 (MHA) assessments and detentions, with Black Africans having higher rates than any other ethnic group.[7] However, when controlling for diagnosis, age, risk and social support there were no significant ethnic differences in detention.[7] Similarly, Singh [8] found no significant differences between ethnic groups in MHA detention whilst controlling for variables such as risk and social support. These studies raise the possibility that treatment differences could be accounted for by ethnic differences in factors such as: self-harm and suicide attempt,[9] psychosis symptom profiles,[10] deprivation,[11] and substance use.[12]

UK national guidelines recommend Cognitive Behavioural Therapy for the treatment and prevention of psychosis (CBTp), as CBTp has demonstrated robust evidence of its

For peer review only - http://bmjopen.bmj.com/site/about/guidelines.xhtml

efficacy on service-user outcomes.[13] However, the National Audit of Schizophrenia found that CBTp was only offered to 39% of service users and accessed by 19% of service users.[14] There are evidently barriers to accessing CBTp (e.g.[15, 16]) although certain factors may increase referral to CBTp (e.g. higher levels of positive symptoms;[17]).

People from ethnic minority communities experience additional barriers to access and engagement with psychological therapy more generally.[18] In the UK, people of Black Caribbean and Black African descent with psychosis are less likely to receive a talking therapy than their White British counterparts.[19-21] A nationally representative survey of people with psychosis found that all ethnic minority groups (excluding those with Mixed ethnicity) were less likely to be offered CBT; and Black service users were less likely to be offered Family Therapy.[2] Similar findings have been demonstrated in international samples, where Black Americans with psychosis are less likely to receive a talking therapy than their White American counterparts.[22] Nonetheless, research emanating from the UK (SLaM IAPT-SMI Demonstration Site) has indicated that after CBTp has been offered there is no difference between a Black and Minority Ethnic (BME) group and a non-BME group in engagement in CBTp.[23, 24]

Engagement is a complex concept that requires the service provider being adequately engaging and the recipient to be adequately engaged. There are potentially many explanations of ethnic variations in access to and engagement with CBT. For example, ethnic minority communities have more coercive pathways into treatment (e.g.[1]) which may adversely influence the therapeutic relationship ([25]), and subsequently impact on engagement in treatment.[26] Other barriers to engagement might include: lower socio-economic status;[26] increased stigma in certain communities;[27] fear of service-users by providers, and fear of providers by service-users;[28] suspiciousness of mental health services and non-culturally appropriate therapy;[29] language barriers;[30] clinicians' perceptions of religious and

For peer review only - http://bmjopen.bmj.com/site/about/guidelines.xhtml

spiritual explanations for psychosis;[31] and institutional racism within mental health services.[32,33]

Research Questions and rationale:

There is a lack of information about the extent of inequalities experienced by ethnic minority groups with serious mental illness, despite well-recognised adverse outcomes in certain minority groups. Furthermore, there is a paucity of information about the role that risk and symptom severity plays in treatment disparity (including access to psychological therapy) for ethnic minority groups. Consequently, using all the case records from a large secondary care mental healthcare provider, this study set out to answer the following questions:

1: In people who have had a diagnosis of bipolar disorder (ICD-10 code F30-1) or psychosis (ICD-10 code F20-29 excluding F21), are there variations by ethnic-group in receipt of either individual or group CBT after adjustment for differences in risk profiles and symptom severity?

2: Do ethnic-group variations in receipt of CBT differ between contexts (e.g. inpatient versus community settings) after adjustment for risk profiles and symptom severity?

Method

Study Design and Setting

The data, which were generated as part of routine care, were derived from clinical records from South London and Maudsley (SLaM) Trust. SLaM is a near-monopoly provider of secondary mental health services[34] for a catchment of over 1.2 million residents in south London and has over 400,000 service user records.[35] The SLaM catchment boroughs are not dissimilar from London as a whole in terms of age, education, gender and socioeconomic status.[35,36] However, SLaM has a higher proportion of ethnic minority groups in

For peer review only - http://bmjopen.bmj.com/site/about/guidelines.xhtml

comparison to England as whole.[35] The (self-assigned) ethnicity population distribution recorded in the 2011 census for the SLaM catchment area is: 55.1% White, 24.7% Black, 10.8% Asian, 6.9% Mixed ethnicity, and 2.5% Other.[35] Even after adjustment for age, sex and ethnicity, areas within SLaM's catchment have been shown to have a 2.2 times higher incidence of psychosis than the European average.[37]

This investigation utilised the Clinical Record Interactive Search (CRIS) tool[35] to access an anonymised data set derived from SLaM's electronic health records that comprise the Maudsley Biomedical Research Centre (BRC) Case Register. The BRC Case Register utilises an opt-out mechanism, which is seldom used (circa N=4). Consequently, the sampling techniques employed ensure that persons who have not experienced good engagement with mental health services are still represented in the sample. Established in 2008, the CRIS system facilitates access and retrieval of anonymised clinical records. For a more in-depth description of how the data are stored, anonymised, and accessed see [35, 36, 38].

Sample

Cases were included if they had received an ICD-10 diagnosis of a bipolar related mental health problem (i.e. manic episode [F30] and/or bipolar affective disorder [F31]) and were defined as having a bipolar disorder. The psychosis group included anyone with any of the following diagnoses: schizophrenia [F20], delusional disorder [F22], brief psychotic disorder [F23], shared psychotic disorder [F24], schizoaffective disorder [F25], other nonorganic psychotic disorders [F28] and unspecified nonorganic psychosis [F29].

No upper limit was set on age. Cases were excluded if: they were under the age of 15 (a criterion which has been previously applied to this cohort;[39]); they had a diagnosis of an organic/non-functional disorder; or there were missing data regarding marital status,

For peer review only - http://bmjopen.bmj.com/site/about/guidelines.xhtml

ethnicity, IMD score, gender, or age. To this end, only participants with complete data were included.

Due to limited numbers in some ethnic groups, cases were excluded if their recorded ethnicity did not belong to one of the following Office of National Statistics categories: Black African, Black Caribbean, Irish, and White British.[40] A group labelled 'South Asian' including individuals recorded as Indian, Pakistani, or Bangladeshi was also included in the sample. This investigation utilised the same approach of defining and grouping ethnicity which has been applied to CRIS data previously.[39, 41]

Data Retrieval

SLaM adopted fully electronic health records for all its services in 2006, including the importing of legacy data. The current data set includes records from the 1st of January 2007 up until the extraction date of the 31st of July 2017. Source clinical records contain information from structured closed question response boxes (e.g. age) and free text. Automated natural language processing (NLP) algorithms (see [42]) are used to determine the presence and prescribed 'value' of variables contained in free text.

Within the current investigation, NLP algorithms were used to provide supplementary information on diagnoses and CBT. Recording an ICD 10[43] diagnosis within a structured field is mandatory within SLaM,[44] supplemented by NLP to ascertain diagnoses recorded in free text sources e.g. clinical notes.[35, 44] Another NLP algorithm has been developed to identify case notes that document a CBT session,[19] again supplementing information within structured fields and achieving in combination a positive predictive value of 95% and a sensitivity of 96%.[19]

For peer review only - http://bmjopen.bmj.com/site/about/guidelines.xhtml

Ethnicity and impact on the receipt of CBT

Demographic, Clinical and Treatment data extracted and operationalised

Demographic data retrieved included gender, marital status, ethnicity and age. All of the demographic data was retrieved at the point of data extraction (31st July 2017), for example the participants age on the 31st of July 2017. From Lower Super Output Area (LSOA) of residence, a standard national geographic unit containing approximately 1500 residents, area level deprivation was calculated from the Index of Multiple Deprivation (IMD).[45] Multiple area level assessments contribute to seven subscales (Income Deprivation; Employment Deprivation; Education, Skills and Training Deprivation; Health Deprivation and Disability; Crime; Barriers to Housing and Services; and Living Environment Deprivation) which form the IMD. Scores on the IMD were split into deciles within the current sample.

The algorithm within the SLaM clinician interface ensures that structured risk assessments are completed when risk information is noted. We developed an assessment of severity and risk based on previous approaches used with this dataset.[46] To this end, we retrieved information from structured risk assessments pertaining to: history of violence, history of 'non-adherence', history of suicide attempt, perceived lethal means used in suicide attempt, current plans to end life, expression of suicidal ideation, expressed feelings of hopelessness, expressed high levels of subjective distress, and expressed feelings of having no control. We also retrieved information about previous: substance use disorder diagnosis (ICD code F1), inpatient admissions, treatment under the Mental Health Act, A&E attendance (for mental health problems), referral to Assertive Outreach, referral to the crisis team, and forensic history.

We retrieved data about the CBT session regarding: whether the service user was an inpatient or outpatient at the time of contact; whether the contact was face-to-face or remote (e.g. via telephone); and whether the contact was in a one to one, or group session. In line

For peer review only - http://bmjopen.bmj.com/site/about/guidelines.xhtml

with National Standard guidelines definition of access,[47] the current investigation assessed whether participants had at least one documented session of CBT. NICE guidelines for psychosis recommend that CBT is delivered "over at least 16 planned session (sic)"[13, p.589]. NICE guidelines for bipolar disorder recommend that a depressive episode should be treated with between 16 to 20 sessions of CBT.[48] Consequently, a 16-session criterion was also adopted as a more stringent definition of a course of CBT. Jolley and colleagues[23] operationalised CBT therapy completion as at least 5 sessions. Supplementary analyses were conducted utilising this less stringent definition of the completion of CBT treatment. Analyses of the 5 and 16 session criteria were restricted to participants who had at least one documented session of CBT (n=5197). Participants were also excluded from analyses regarding the 5 and 16 session criteria if they were currently receiving CBT at data extraction and had not received a minimum of 5 or 16 sessions of CBT, which resulted in n=100 and n=220 participants being excluded respectively (see Figure 1). CBT that was currently on-going was defined as anyone who had a CBT session in the 6 weeks prior to data extraction.

Ethical Considerations

The anonymised dataset has been approved by the NHS REC for secondary analysis (Oxford C Research Ethics Committee, reference18/SC/0372). This particular project received ethical approval from the Lancaster University Faculty of Health and Medicine Research Ethics Committee and approval from the CRIS Oversight Committee.

Patient and Public Involvement

This specific project was reviewed, commented on and approved by the CRIS Oversight Committee, which is chaired by a service user representative. Furthermore, the development of the CRIS system was informed by consultation with service users.[38]

For peer review only - http://bmjopen.bmj.com/site/about/guidelines.xhtml

Ethnicity and impact on the receipt of CBT

Analysis

Logistic regression models were built using multivariable procedures in Stata 12. Models were adjusted for demographic data (gender, age, IMD, and marital status), diagnoses (psychosis/bipolar disorder), and risk/severity variables (as described previously). Analysesare presented as: crude associations; adjustments for demographic data and diagnosis (Step 1); and adjustments for demographic data, diagnosis and the risk/severity variables (Step 2).

Results

Descriptive Statistics

A total of 5351 cases were excluded due to missing data relating to marital status (n=3678), Index of Multiple Deprivation (n=1308), ethnicity (n=362), gender (n=2) and age (n=1).The final sample consisted of 20,010 cases, Figure 1 displays the flow of cases through the study.

(FIGURE 1)

The majority of cases were White British (n = 10393, 51.9%), the next largest ethnic group were Black Caribbean people who made up 27.4% of the sample (n=5481). There were more male cases (n=10457, 52.3%) than female and the majority were single (n=17097, 85.4%). Table 1 summarises the demographic and diagnosis data (at the time of data extraction) with relevant proportions for each ethnic group. Further information on treatment, risk, and severity including items from the structured risk assessment can be found in Supplementary Table 1.

(TABLE 1)

For peer review only

Ethnicity and impact on the receipt of CBT

Just over a quarter of the sample (26.0%, n=5197) had a documented session of CBT in the study period. The median number of sessions of CBT was 5 (IQR 13). Considering all CBT sessions documented, most were delivered face to face, at a ratio of approximately 30 face to face sessions for every 1 remote (e.g. telephone) session delivered, and as individual rather than group sessions at a ratio of approximately 17:1. Of the people who had received CBT, 30% had their first ever (documented) session as an inpatient, 55.4% had ≥5 sessions and 25.8% had received ≥16 sessions. Further information about CBT can be found in Supplementary Table 2.

Ethnicity and reported receipt of CBT as an inpatient or outpatient.

Table 2 displays the unadjusted and adjusted ORs for having a reported session of CBT in relation to ethnicity and covariates. The final adjusted model indicated that the Black African group were significantly less likely to receive CBT than the White British group (OR 0.73, CI 0.66 to 0.82, p<.001), after risk indicators were taken into account. In the adjusted model, several factors related to risk and severity were independently associated with increased likelihood of reported receipt of CBT, including lifetime inpatient admission, history of non-adherence, history of suicide attempt, lethal means used in suicide attempt, suicidal ideation, feelings of hopelessness, high levels of distress, no feelings of control, and referral to the crisis team. However, a history of a substance misuse disorder diagnosis and plans to end life were associated with a decreased likelihood of reported receipt of CBT.

(TABLE 2)

Ethnicity and a minimum of 16 CBT sessions

Table 3 displays the unadjusted and adjusted ORs of receiving a minimum of 16 sessions of CBT in relation to ethnicity and covariates. The adjusted model indicated that the Black Caribbean group were significantly less likely to receive a minimum of 16 sessions of

For peer review only - http://bmjopen.bmj.com/site/about/guidelines.xhtml

Ethnicity and impact on the receipt of CBT

CBT than the White British group (OR 0.83, CI 0.71 to 0.98, p=.03). The model also indicated that receiving the first session of CBT as an inpatient was associated with decreased odds of having at least 16 sessions of CBT (OR 0.35, CI 0.29 to 0.42, p<.001) and some of the indicators of risk increased the odds of receiving CBT (history of suicide attempt, reported high levels of distress and lifetime referral to crisis team). However, several factors associated with increased odds of ever receiving a documented session of CBT (Table 2) were not significantly associated with having a minimum of 16 documented sessions (i.e. lifetime inpatient admittance, history of non-adherence, lethal means used in suicide attempt, reported suicidal ideation, reported feelings of hopelessness, reported feelings of a lack of control).

TABLE 3

Ethnicity and reported receipt of CBT as an inpatient

Analyses were restricted to participants who had been an inpatient (N= 9417) and associations investigated with receipt or not of CBT in this setting. Unadjusted and adjusted associations are displayed in Table 4. The adjusted model demonstrated that the Black African group (OR 0.76, CI 0.65 to 0.89, p=.001) and the Black Caribbean group (OR 0.83, CI 0.73 to 0.94, p=.003) were significantly less likely to have received CBT than the White British group.

TABLE 4

Supplementary Analyses

Analyses using the less stringent definition of a course of CBT (≥5 sessions) indicated the Black African group were significantly less likely to receive this in comparison to the White British group (OR 0.76, CI 0.63 to 0.91, p=.003) (see Supplementary Table 3). Analyses of CBT sessions received only as an outpatient also indicated that the Black African

For peer review only - http://bmjopen.bmj.com/site/about/guidelines.xhtml

Ethnicity and impact on the receipt of CBT

group (OR 0.75, CI 0.67 to 0.84, p<.001) were significantly less likely to receive this than the White British group (see Supplementary Table 4).

Post-Hoc Sensitivity Analysis

1. Recording of clinical risk

The crude estimates indicated that several variables indicative of higher clinical risk and severity were associated with increased odds of having a (single) documented session of CBT (Table 2). We considered that this may be because CBT is better recorded (rather than more likely to be delivered) for those at an increased risk (e.g. of harming themselves, suicide, harming others) and proposed that, if defensive practice resulted in better note keeping, this would be most likely evident in the structured fields. Consequently, as a supplementary sensitivity analysis, using the entire sample (N=20,010), models assessing reported receipt of CBT were re-run omitting entries identified in the structured fields, (i.e. just using data derived from free text). However, this analysis continued to indicate an association between Black African group membership and significantly lower odds of receiving CBT than White British group membership (OR 0.76, CI 0.63 to 0.92, p=.004). Adjusted and unadjusted odds ratios are presented in Supplementary Table 5.

2. Influence of time

Additional analyses were conducted to assess if changes over time affected referral practices for psychological treatments. To this end, a variable was created indicating participants who had received a diagnosis of psychosis or bipolar affective disorder after the mid-point of the data collection window (i.e. after the 16th of April 2012). Models considering ethnicity and reported receipt of CBT were re-run including the variable indicating the date at which diagnosis was given. This analysis also indicated that the Black

For peer review only - http://bmjopen.bmj.com/site/about/guidelines.xhtml

Ethnicity and impact on the receipt of CBT

African group were significantly less likely to receive CBT than the White British group (OR 0.72, CI 0.65 to 0.81, p<.001), suggesting that this finding was not influenced by the date diagnosis was given (see Supplementary Table 6). In the fully adjusted model, receiving a diagnosis of psychosis or bipolar affective disorder after the midpoint of the data collection window was associated with decreased odds of a documented session of CBT (OR 0.77, CI 0.71 to 0.83, *p*<.001). Further, analysis was conducted to assess if there was an interaction between time and ethnicity; however, a likelihood ratio test indicated that fitting this interaction term did not significantly improve the model: Chi^2 (4) =5.25, *p*= .26.

Discussion

Statement of principal findings

This investigation found that after adjustment for numerous indicators of risk and severity, in comparison to White British counterparts, Black African people with bipolar disorder or psychosis were less likely to have a documented session of CBT, a finding which was robust to a number of sensitivity analyses. After adjustment for indicators of risk and symptom severity in comparison to White British people, Black Caribbean people were also less likely to receive CBT as inpatients, and were less likely to receive the minimum 16 sessions recommended by national guidelines. This study also found that regardless of ethnicity people who had their first documented session of CBT as an inpatient were less likely to receive a minimum of 16 sessions of CBT (and a similar effect was also noted in supplementary analyses of a minimum 5 documented sessions and documented receipt of CBT as an outpatient). In addition, regardless of ethnicity indicators of higher risk and severity of symptoms were typically associated with higher odds of receiving CBT; however, these associations between risk status and receipt of CBT were less consistent in analyses of a minimum 16 documented sessions.

For peer review only - http://bmjopen.bmj.com/site/about/guidelines.xhtml

Ethnicity and impact on the receipt of CBT

Strengths and limitations of the study

To our knowledge, this study has used the largest sample to date to assess ethnic differences in access to CBT for people with psychosis or bipolar affective disorder. This study utilised a case register from a large mental healthcare provider serving a socially and ethnically diverse geographic catchment. Furthermore, the data were sourced from the full electronic health record, using a case register with near-complete coverage of people receiving mental healthcare for these diagnoses. The study utilised a tool to extract information about CBT from structured fields and free text, an approach which has been shown to have high positive predictive value and sensitivity values in previous work.[19] Consequently, this study likely provides a highly accurate picture of access to CBT delivered by mental health services within the catchment. Of note, despite having recognised high incidence rates of psychosis,[37] the catchment is not dissimilar to other parts of London and UK urban areas on several sociodemographic metrics;[35, 36] the results of this investigation may generalise to other urban and semi-urban multicultural areas in England, a notion which is supported by ethnic disparity in access to therapy indicated in nationally representative data.[2] By accessing a large data set of complete clinical records we were able to contribute novel findings relating to the impact of risk and pathways on engagement with CBT. However, one limitation of this investigation is that it was not possible to extract information from the BRC Case Register about other psychological therapies, some of which are recommended by national guidelines and delivered routinely within the services analysed (e.g. Family Intervention;[13]). It is possible therefore that disparity in access to CBT may be accounted for by ethnic differences in preference for therapy type, although this has not been suggested to be the case in other studies of national data from the UK.[2] Another limitation is that although this study likely displays an accurate picture of service users who *received* CBT it was not possible to derive information about the *offer* of CBT. If service users are not

For peer review only - http://bmjopen.bmj.com/site/about/guidelines.xhtml

Ethnicity and impact on the receipt of CBT

accepting CBT or completing a course, or alternatively service providers are not offering or delivering a course of CBT, it is important to understand why. This could be explored in future research.

An additional limitation of this study is we did not extract information regarding the length of inpatient stay. The consequence of this is we do not know the impact of length of stay on the likelihood that someone receives CBT. It is feasible that people who have very short inpatient stays are less likely to receive CBT than those who spend longer in that environment.

Strengths of this study in relation to other research

Our findings replicate those observed for unselected community residents from a nationally representative sample, namely less equitable access to CBT for ethnic minority groups.[2] Previous investigations which have explored ethnic disparities in access/engagement with CBT in samples with psychosis have not differentiated between Black African and Black Caribbean people,[2, 19, 23, 24] despite the two groups typically having different migratory histories and different factors influencing pathways into treatment for psychosis.[49] The current investigation was able to define more specific ethnic categories providing a more nuanced understanding of ethnicity and access to CBT.

Comparisons with previous research

Previous research has highlighted that more positive symptoms in psychosis increase referrals for CBT.[17] Our study extended this finding by highlighting that numerous indicators of higher symptom severity and risk increase the propensity to receive a minimum of one session of CBT. However, despite controlling for these variables, this study found persistent disparities by ethnicity in receipt of CBT (i.e. a minimum of one documented session). The relationship between risk and CBT engagement (i.e. documented receipt of a

For peer review only - http://bmjopen.bmj.com/site/about/guidelines.xhtml

Ethnicity and impact on the receipt of CBT

minimum of 16 sessions) appeared less consistent. Several of the risk indicators which increased the odds of receiving one documented session of CBT were not significantly associated either way with receipt of a minimum of 16 sessions. This may suggest a more complex relationship between risk and CBT engagement. The positive association between recorded level of clinical risk and receipt of CBT is in contrast to research suggesting that inequalities between ethnic groups in mental health treatment could be caused by differences in symptom severity.[7, 8] Despite risk indicators (typically) increasing access to CBT and previous investigations suggesting that Black women are most likely to self-harm;[50] the current investigation does not indicate that ethnic disparities in the receipt of CBT is as a consequence of ethnic differences in risk or symptom profile.

First access of CBT as an inpatient was associated with lower odds of receiving further CBT sessions. There are numerous potential explanations. For example, coercive practice in inpatient settings has been well documented and this may potentially impact on subsequent engagement.[51] Alternatively, our finding may be related to differences in recovery styles.[52] An avoidant recovery style (referred to as sealing over) has been linked to poorer engagement with services,[53] and it is possible that some people are receptive to psychological therapy at the point of crisis (i.e. during inpatient stay), but once there is a diminution of symptoms they 'seal over' which reduces engagement.

Implications of this research and suggestions for future research

Our study suggests that, within clinical settings, further work is needed to ensure there is parity in access to CBT. In practice, this might include ensuring that CBT is systematically offered to groups who are less likely to receive treatment. It is also feasible that further work is needed to ensure that CBT is more acceptable to Black groups which might be achieved by culturally-adapting interventions.[54] Nonetheless, more research is required to explore the reasons underpinning ethnicity difference in access to CBT, whether ethnic differences in

For peer review only - http://bmjopen.bmj.com/site/about/guidelines.xhtml

receipt of CBT extend to the offer of CBT, and the impact clinical risk has on engagement with CBT. Moreover, further research is necessary to explore the impact of pathways into care or psychological treatment, and its role in subsequent engagement.

For peer review only

Ethnicity and impact on the receipt of CBT

Acknowledgements

We are grateful to Hitesh Shetty for his work extracting data from the BRC case register and his advice on usage of BRC CRIS data. We are grateful to Megan Pritchard for her advice on usage of BRC CRIS data and her support in training RM on accessing CRIS data. We are grateful to Dr Emmanuelle Peters for her advice about the study and her advice about psychological therapy service delivery within SLaM.

Declaration of Funding

JD is funded by the Health Foundation working together with the Academy of Medical Sciences. RS is part-funded by the National Institute for Health Research (NIHR) Biomedical Research Centre at South London and Maudsley NHS Foundation Trust and King's College London, and by an NIHR Senior Investigator award. RM is funded by Lancashire Care Foundation Trust. The authors declare that the study funders have had no role in the study design; in the collection, analysis and interpretation of the data; in the writing of the report; and in the decision to submit the paper for publication.

This paper represents independent research funded by the National Institute for Health Research (NIHR) Biomedical Research Centre at South London and Maudsley NHS Foundation Trust and King's College London. The views expressed are those of the author(s) and not necessarily those of the NHS, the NIHR or the Department of Health and Social Care

Competing Interests

SLaM and its services have had no role, in the study design, in the analysis and interpretation of the data, in the writing of the report, or in the decision to submit the paper for publication. Caroline Cupitt is employed by SLaM and works within one of SLaM's

For peer review only - http://bmjopen.bmj.com/site/about/guidelines.xhtml

services which has produced some of the clinical notes that form part of the data analysed herein. RS declares research funding within the last 5 years from Roche, GSK and Janssen.

Contributorship statement

RM, JD, WS, and DE made substantial contributions to the conception and design of the work and interpretation of the data. RM and JD made a substantial contribution to the analysis of the data. RS made a substantial contribution to the conception and design of the work and acquisition of the data. Craig Colling made a substantial contribution to the acquisition of the data. Caroline Cupitt made a substantial contribution to the interpretation of the data. All authors contributed to reviewing, drafting and revising of the manuscript. All authors have provided their approval for the work to be published and are in agreement to be accountable for all aspects of the work in ensuring that questions related to the accuracy or integrity of any part of the work are appropriately investigated and resolved.

Data sharing statement

Data are owned by the South London and Maudsley NHS Foundation Trust (SLaM) which provides access to anonymised data derived from electronic medical records via the Clinical Record Interactive Search (CRIS) system. These data can only be accessed by permitted individuals from within a secure firewall (i.e. remote access is not possible and the data cannot be sent elsewhere) in the same manner as the authors. For data requests please contact Cris.administrator@slam.nhs.uk

For peer review only - http://bmjopen.bmj.com/site/about/guidelines.xhtml

Ethnicity and impact on the receipt of CBT

References

1 Mann F, Fisher HL, Major B, et al. Ethnic variations in compulsory detention and hospital admission for psychosis across four UK Early Intervention Services. *BMC Psychiatry* 2014;14(1):256. https://doi.org/10.1186/s12888-014-0256-1

2 Das-Munshi J, Bhugra DKML, Crawford M. Ethnic minority inequalities in access to treatments for schizophrenia and schizoaffective disorders. Findings from a nationally representative cross-sectional study. *BMC Med* 2018;16(1):55 https://doi.org/10.1186/s12916-018-1035-5

3 Evans R, Makala J, Humphreys M, et al. Supervised community treatment in Birmingham and Solihull: first 6 months. *Psychiatrist* 2010; 34(8): 330–333. https://doi.org/DOI:10.1192/pb.bp.109.027482

4 Horvitz-Lennon M, McGuire TG, Alegria M, et al. Racial and ethnic disparities in the treatment of a Medicaid population with schizophrenia. *Health Serv Res* 2009;44(6):2106–2122. https://doi.org/10.1111/j.1475-6773.2009.01041.x

5 Daumit GL, Crum RM, Guallar E, et al. Outpatient prescriptions for atypical antipsychotics for African Americans, Hispanics, and whites in the United States. *Arch Gen Psychiatry* 2003;60(2):121–128. https://doi:10.1001/archpsyc.60.2.121

6 Vinkers DJ, de Beurs E, Barendregt M, et al. Pre-trial psychiatric evaluations and ethnicity in the Netherlands. *Int J Law Psychiatry* 2010;33(3):192–196. https://doi.org/10.1016/j.ijlp.2010.03.010

7 Gajwani R, Parsons H, Birchwood M, et al. Ethnicity and detention: are Black and minority ethnic (BME) groups disproportionately detained under the Mental Health Act

For peer review only - http://bmjopen.bmj.com/site/about/guidelines.xhtml

2007? *Soc Psychiatry Psychiatr Epidemiol* 2016;51(5):703–711. https://doi.org/10.1007/s00127-016-1181-z

8 Singh SP, Burns T, Tyrer P, et al. Ethnicity as a predictor of detention under the Mental Health Act. *Psychol Med* 2014;44(5):997–1004. https://doi.org/10.1017/S003329171300086X

9 Al-Sharifi A, Krynicki CR, Upthegrove R. Self-harm and ethnicity: A systematic review. *Int J Soc Psychiatry* 2015;61(6):600–612. https://doi.org/10.1177/0020764015573085

10 Veling W, Selten JP, Mackenbach JP. Symptoms at first contact for psychotic disorder: comparison between native Dutch and ethnic minorities. *Schizophr Res* 2007;95(1–3):30–38. https://doi.org/10.1016/j.schres.2007.06.024

11 Jivraj S, Khan O. Ethnicity and deprivation in England: How likely are ethnic minorities to live in deprived neighbourhoods? Centre on Dynamics of Ethnicity briefing paper 2013 http://hummedia.manchester.ac.uk/institutes/code/briefingsupdated/ethnicity-and-deprivation-in-england-how-likely-are-ethnic-minorities-to-live-in-deprived-neighbourhoods%20(1).pdf (accessed 1 Feb 2018)

12 Shih RA, Miles JNV, Tucker JS, et al. Racial/ethnic differences in adolescent substance use: mediation by individual, family, and school factors. *J Stud Alcohol Drugs* 2010;71(5):640–651.

13 National Institute for Health and Care Excellence. Psychosis and schizophrenia in adults: The NICE guideline on treatment and management (CG178). London, UK: NICE 2014

14 Royal College of Psychiatrists. Report of the second round of the National Audit of Schizophrenia (NAS2) 2014. London, UK: Royal College of Psychiatrists Centre for Quality Improvement 2014

For peer review only - http://bmjopen.bmj.com/site/about/guidelines.xhtml

Ethnicity and impact on the receipt of CBT

15 Hazell CM, Strauss C, Cavanagh K, et al. Barriers to disseminating brief CBT for voices from a lived experience and clinician perspective. *PloS One* 2017;12(6):e0178715. https://doi.org/10.1371/journal.pone.0178715

16 Prytys M, Garety PA, Jolley S, et al. Implementing the NICE guideline for schizophrenia recommendations for psychological therapies: a qualitative analysis of the attitudes of CMHT staff. *Clin Psychol Psychother* 2011;18(1):48–59. https://doi.org/10.1002/cpp.691

17 Fanning F, Foley S, Lawlor E, et al. Group cognitive behavioural therapy for first episode psychosis: who's referred, who attends and who completes it? *Early Interv Psychiatry* 2012;6(4):432–441. https://doi.org/10.1111/j.1751-7893.2011.00333.x

18 Memon A, Taylor K, Mohebati LM, et al. Perceived barriers to accessing mental health services among black and minority ethnic (BME) communities: a qualitative study in Southeast England. *BMJ Open* 2016;6(11):e012337. https://doi.org/10.1136/bmjopen-2016-012337

19 Colling C, Evans L, Broadbent M, et al. Identification of the delivery of cognitive behavioural therapy for psychosis (CBTp) using a cross-sectional sample from electronic health records and open-text information in a large UK-based mental health case register. *BMJ Open* 2017;7(7): e015297. http://dx.doi.org/10.1136/bmjopen-2016-015297

20 McKenzie K, Samele C, Horn E, et al. Comparison of the outcome and treatment of psychosis in people of Caribbean origin living in the UK and British Whites. *British J Psychiat* 2001;178(2),160-165. https://doi.org/10.1192/bjp.178.2.160

21 McKenzie K, van Os J, Fahy T, et al. Psychosis with good prognosis in Afro-Caribbean people now living in the United Kingdom. *BMJ* 1995;311(7016):1325–1328. https://doi.org/10.1136/bmj.311.7016.1325

For peer review only - http://bmjopen.bmj.com/site/about/guidelines.xhtml

22 Walkup J, Wei W, Sambamoorthi U, et al. Provision of psychotherapy for a statewide population of medicaid beneficiaries with schizophrenia. *Psychol Serv*, 2006;3(4):227–238. https://doi.org/10.1037/1541-1559.3.4.227

23 Jolley S, Garety P, Peters E, et al. Opportunities and challenges in Improving Access to Psychological Therapies for people with Severe Mental Illness (IAPT-SMI): evaluating the first operational year of the South London and Maudsley (SLaM) demonstration site for psychosis. *Behav Res Ther* 2015;64:24–30. https://doi.org/10.1016/j.brat.2014.11.006

24 Johns L, Jolley S, Garety P, et al. Improving Access to psychological therapies for people with severe mental illness (IAPT-SMI): Lessons from the South London and Maudsley psychosis demonstration site. *Behav Res Ther* 2019;116:104–10. https://doi.org/10.1016/j.brat.2019.03.002

25 Sweeney A, Fahmy S, Nolan F, et al. The relationship between therapeutic alliance and service user satisfaction in mental health inpatient wards and crisis house alternatives: a cross-sectional study. *PloS One* 2014;9(7):e100153. https://doi.org/10.1371/journal.pone.0100153

26 Barrett MS, Chua W-J, Crits-Christoph P, et al. Early withdrawal from mental health treatment: Implications for psychotherapy practice. *Psychotherapy (Chic)* 2008;45(2): 247–267. http://dx.doi.org/10.1037/0033-3204.45.2.247

26 Holdsworth E, Bowen E, Brown S, et al. The Development of a Program Engagement Theory for Group Offending Behavior Programs. *Int J Offender Ther Comp Criminol* 2016;*61*(13):1479–1499. https://doi.org/10.1177/0306624X15624177

27 Mantovani N, Pizzolati M, Edge D. Exploring the relationship between stigma and help-seeking for mental illness in African-descended faith communities in the UK. *Health Expect* 2017;20(3):373–384. https://doi.org/10.1111/hex.12464

For peer review only - http://bmjopen.bmj.com/site/about/guidelines.xhtml

28 Keating F, Robertson D, McCulloch A, et al. Breaking the Circles of Fear: A review of the relationship between mental health services and African and Caribbean communities. London, UK: The Sainsbury Centre for Mental Health 2002

29 Rathod S, Kingdon D, Phiri P, et al. Developing culturally sensitive cognitive behaviour therapy for psychosis for ethnic minority patients by exploration and incorporation of service users' and health professionals' views and opinions. *Behav Cogn Psychother* 2010;38(5):511-533. https://doi.org/10.1017/S1352465810000378

30 Chen J, Rizzo J. Racial and ethnic disparities in use of psychotherapy: evidence from U.S. national survey data. *Psychiatric Serv* 2010;61(4):364–372. https://doi.org/10.1176/ps.2010.61.4.364

31 Islam Z, Rabiee F, Singh SP. Black and Minority Ethnic Groups' Perception and Experience of Early Intervention in Psychosis Services in the United Kingdom. *J Cross Cult Psychol* 2015;46(5):737-753. https://doi:10.1177/0022022115575737

32 Blofeld J. Independent Inquiry into the death of David Bennett. Cambridgshire, Norfolk, Suffolk and Cambridgeshire, UK: Strategic Health Authority 2003

33 Royal College of Psychiatrists. Racism and mental health: Position Statement 01/18. London, UK: Royal College of Psychiatrists 2018

34 Lally J, Wong YL, Shetty H, et al. Acute hospital service utilization by inpatients in psychiatric hospitals. *Gen Hosp Psychiatry*, 2015;37(6):577–580. https://doi.org/10.1016/j.genhosppsych.2015.07.006

35 Perera G, Broadbent M, Callard F, et al. (2016) Cohort Profile of the South London and Maudsley NHS Foundation Trust Biomedical Research Centre (SLaM BRC) Case Register:

For peer review only - http://bmjopen.bmj.com/site/about/guidelines.xhtml

Current Status and Recent Enhancements of an Electronic Mental Health Record Derived Data Resource. *BMJ Open* 2016;6:e008721. https://doi.org/10.1136/bmjopen-2015-008721

36 Stewart R, Soremekun M, Perera G, et al. The South London and Maudsley NHS Foundation Trust Biomedical Research Centre (SLAM BRC) Case Register: Development and Descriptive Data. *BMC Psychiatry* 2009;9:51. https://doi.org/10.1186/1471-244X-9-51

37 Jongsma HE, Gayer-Anderson C, Lasalvia A, et al. Treated incidence of psychotic disorders in the multinational eu-gei study. *JAMA Psychiatry* 2018;75(1):36–46. http://dx.doi.org/10.1001/jamapsychiatry.2017.3554

38 Fernandes AC, Cloete D, Broadbent M, et al. Development and Evaluation of a De-Identification Procedure for a Case Register Sourced from Mental Health Electronic Records. *BMC Psychiatry* 2013;13:71. https://doi.org/10.1186/1472-6947-13-71

39 Das-Munshi J, Chang C-K, Dutta R, Ethnicity and excess mortality in severe mental illness: a cohort study. *Lancet Psychiatry* 2018;4(5):389–399. https://doi.org/10.1016/S2215-0366(17)30097-4

40 Office for National Statistics. Measuring equality: A guide for the collection and classification of ethnic group, national identity and religion data in the UK. (N.D.) https://www.ons.gov.uk/methodology/classificationsandstandards/measuringequality/ethnicgroupnationalidentityandreligion (Accessed 4 Oct 2019)

41 Das-Munshi J, Ashworth M, Gaughran F, et al. Ethnicity and cardiovascular health inequalities in people with severe mental illnesses: protocol for the E-CHASM study. *Soc Psychiatry Psychiatr Epidemiol* 2016;*51*(4):627–638. https://doi.org/10.1007/s00127-016-1185-8

42 Aggarwal C, Zhai C. Mining text data. New York, USA: Springer 2012

For peer review only - http://bmjopen.bmj.com/site/about/guidelines.xhtml

43 World Health Organisation. International Statistical Classification of Diseases and Related Health Problems 10th version. Geneva, Switzerland: World Health Organisation. 2010 http://apps.who.int/classifications/icd10/browse/2016/en (accessed 9 Aug 2017)

44 Jackson RG, Patel R, Jayatilleke N, et al. Natural language processing to extract symptoms of severe mental illness from clinical text: the Clinical Record Interactive Search Comprehensive Data Extraction (CRIS-CODE) project. *BMJ Open* 2017;7: e012012 http://dx.doi.org/10.1136/bmjopen-2016-012012

45 Department for Communities and Local Government. The English Index of Multiple Deprivation (IMD) 2015 – Guidance. London, England: Home Office. 2015 https://assets.publishing.service.gov.uk/government/uploads/system/uploads/attachment_data/file/464430/English_Index_of_Multiple_Deprivation_2015_-_Guidance.pdf (accessed 26 Feb 20)

46 Woodhead C, Ashworth M, Broadbent M, et al. Cardiovascular disease treatment among patients with severe mental illness: a data linkage study between primary and secondary care. *Br J Gen Pract* 2016;*66*(647):e374–e381. https://doi.org/10.3399/bjgp16X685189

47 National Health Service England. Implementing the Early Intervention in Psychosis Access and Waiting Time Standard: Guidance. London, UK: NHS England: National Collaborating Centre for Mental Health, National Institute for Health and Care Excellence. 2016 https://www.england.nhs.uk/mentalhealth/wp-content/uploads/sites/29/2016/04/eip-guidance.pdf

48 National Institute for Health and Care Excellence. Bipolar disorder: assessment and management (CG185). London, UK: NICE 2014

49 Bhui K, Ullrich S, Kallis C, et al. Criminal justice pathways to psychiatric care for psychosis. *Br J Psychiatry* 2015;207(6):523–529. http://doi:10.1192/bjp.bp.114.153882

For peer review only - http://bmjopen.bmj.com/site/about/guidelines.xhtml

Ethnicity and impact on the receipt of CBT

50 Cooper J, Murphy E, Webb R, et al. (2010). Ethnic differences in selfharm, rates, characteristics and service provision: Three city cohort study. *Br J Psychiatry* 2010;197:212–218. http://doi:10.1192/bjp.bp.109.072637

51 Department of Health and Social Care. Modernising the Mental Health Act: increasing choice, reducing compulsion. London, England: Home Office. 2018 https://assets.publishing.service.gov.uk/government/uploads/system/uploads/attachment_data/file/762206/MHA_reviewFINAL.pdf

52 Drayton M, Birchwood M, Trower P. Early attachment experience and recovery from psychosis. *Br J Clin Psychol* 1998;37:269–284. https://doi.org/10.1111/j.2044-8260.1998.tb01385.x

53 Tait L, Birchwood M, Petertrower P. Adapting to the challenge of psychosis: Personal resilience and the use of sealing-over (avoidant) coping strategies. *Br J Psychiatry* 2004;185(5):410-415. https://doi.org/10.1192/bjp.185.5.410

54 Rathod S, Phiri P, Harris S, et al. Cognitive behaviour therapy for psychosis can be adapted for minority ethnic groups: A randomised controlled trial. *Schizophr Res* 2013;143, 319-326. http://dx.doi.org/10.1016/j.schres.2012.11.007

For peer review only

For peer review only - http://bmjopen.bmj.com/site/about/guidelines.xhtml

Tables

For peer review only

For peer review only - http://bmjopen.bmj.com/site/about/guidelines.xhtml

Table 1

Information on baseline demographics and diagnoses and their relevant proportions for each ethnic group

		White British		Irish		African		Caribbean		South Asian		Total		Chi² (DF)	*p* value
		N	%	N	%	N	%	N	%	N	%				
Ethnicity		10393	51.9	570	2.8	2817	14.1	5481	27.4	749	3.7	20010			
Female		5070	48.8	269	47.2	1350	47.9	2497	45.6	367	49	9553	47.7		
Male		5323	51.2	301	52.8	1467	52.1	2984	54.4	382	51	10457	52.3	15.6	<.01
Index of Multiple Deprivation**	1	1489	14.3	42	7.4	70	2.5	199	3.6	59	7.9	1859	9.3	1000.0 (36)*	<.001
	2	1160	11.2	53	9.3	165	5.9	456	8.3	92	12.3	1926	9.6		
	3	1133	10.9	62	10.9	195	6.9	536	9.8	87	11.6	2013	10.1		
	4	1041	10.0	53	9.3	284	10.1	542	9.9	86	11.5	2006	10.0		
	5	980	9.4	58	10.2	302	10.7	584	10.7	82	11.0	2006	10.0		
	6	920	8.9	62	10.9	327	11.6	654	11.9	69	9.2	2032	10.2		
	7	933	9.0	60	10.5	326	11.6	617	11.3	80	10.7	2016	10.1		
	8	919	8.8	59	10.4	407	14.5	651	11.9	54	7.2	2090	10.4		
	9	867	8.3	60	10.5	379	13.5	646	11.8	64	8.5	2016	10.1		
	10	951	9.2	61	10.7	362	12.9	596	10.9	76	10.2	2046	10.2		
Single marital status		8784	84.5	486	85.3	2300	81.7	5035	91.9	492	65.7	17097	85.4	456.4 (4)*	<.001
In relationship		1609	15.5	84	14.7	517	18.4	446	8.1	257	34.3	2913	14.6		

For peer review only

Ethnicity and impact on the receipt of CBT

Age: median (IQR)	49 (26.9)		56 (28.8)		43 (18.8)		46 (22.3)		47 (26.2)		48 (24.5)		451.1 (4)*	<.001
Psychosis***	6516	62.7	366	64.2	2435	86.4	4617	84.2	563	75.2	14497	72.5	1200.0 (4) *	<.001
Bipolar****	3877	37.3	204	35.8	382	13.6	864	15.8	186	24.8	5513	27.6		
Lifetime Comorbid substance use diagnosis	1675	16.1	140	24.6	292	10.4	865	15.8	53	7.1	3025	15.1	94.4 (4) *	<.001

*Kruskal-Wallis H non parametric test for ranked data used to determine the Chi^2 value ; **1= least deprived,10= most deprived; ***Psychosis= diagnosis of schizophrenia, delusional disorder, brief psychotic disorder, shared psychotic disorder, schizoaffective disorder, Other nonorganic psychotic disorders, or Unspecified nonorganic psychosis; ****Bipolar= diagnosis of a Manic episode or Bipolar affective disorder.

For peer review only

For peer review only - http://bmjopen.bmj.com/site/about/guidelines.xhtml

For peer review only

Ethnicity and impact on the receipt of CBT

Table 2

Crude and adjusted associations from logistic regression models for at least one recorded session of CBT (inpatient or outpatient)

Variable	N	Odds Ratio (95% Confidence Interval)		
		Crude Associations	Step 1	Step 2
**Ethnicity**				
White British	10393	Reference group		
Irish	570	1.00 (0.82-1.21)	1.12 (0.91-1.36)	1.05 (0.85-1.29)
Black African	2817	1.06 (0.97-1.17)	0.96 (0.87-1.06)	0.73 (0.66-0.82) ***
Black Caribbean	5481	1.29 (1.20-1.39) ***	1.20 (1.11-1.30) ***	0.93 (0.86-1.02)
South Asian	749	0.99 (0.83-1.18)	0.97 (0.82-1.16)	0.93 (0.77-1.12)
**Gender**				
Female	9553	Reference group		
Male	10457	0.89 (0.84-0.95) ***	0.84 (0.78-0.89) ***	0.84 (0.78-0.90) ***
**Age** (years)		0.98 (0.98-0.99) ***	0.98 (0.98-0.99) ***	0.99 (0.98-0.99) ***
**Area level deprivation**				
IMD decile (per tenth)		1.01 (1.00-1.02)	1.01 (0.99-1.02)	0.99 (0.98-1.00)
**Marital status**				
In relationship	2913	Reference group		
Single	17097	1.23 (1.12-1.35)	1.08 (0.98-1.19)	1.07 (0.97-1.18)
**Diagnosis**				
Psychosis	14497	Reference Group		
Bipolar affective disorder	5513	0.94 (0.88-1.01)	0.93 (0.86-1.00)	1.00 (0.93-1.09)
**Comorbid substance misuse**				
No previous substance misuse diagnosis	16985	Reference group		
Lifetime comorbid substance misuse diagnosis	3025	1.31 (1.20-1.42) ***		0.85 (0.77-0.93)**
**Admission**				
No previous admission	10593	Reference Group		
Inpatient Admission Ever	9417	3.20 (2.99-3.42) ***		1.76 (1.58-1.95) ***
**Treatment under the Mental Health Act**				
Never treated under Mental Health Act	12904	Reference Group		
Ever treated under Mental Health Act	7106	2.54 (2.38-2.71) ***		0.96 (0.87-1.07)
**Structured risk assessment items#**				
History of Violence	6216	2.31 (2.16-2.47) ***		1.09 (1.00-1.20)
Difficulty managing physical health	3622	1.74 (1.61-1.88) ***		0.97 (0.88-1.07)
History of Non-adherence	6425	2.55 (2.39-2.73) ***		1.27 (1.16-1.39) ***
History of Suicide Attempt	3758	2.83 (2.63-3.05) ***		1.36 (1.22-1.53) ***
Lethal means used in suicide attempt	2026	2.65 (2.41-2.91) ***		1.04 (1.22-1.53) ***

For peer review only - http://bmjopen.bmj.com/site/about/guidelines.xhtml

For peer review only

Ethnicity and impact on the receipt of CBT

Plans to end life	863	2.62 (2.29-3.01) ***	0.82 (0.69-0.96) *
Suicidal ideation	2041	3.23 (2.94-3.55) ***	1.24 (1.10-1.41) **
Feelings of hopelessness	2850	3.06 (2.82-3.32) ***	1.24 (1.11-1.40) ***
High level of distress	4666	3.24 (3.02-3.47) ***	1.53 (1.40-1.68) ***
No feelings of control	2972	3.03 (2.79-3.28) ***	1.22 (1.09-1.36) ***
**Referred/seen by other team**			
Never referred to Crisis team	13504	Reference Group	
Ever referred to the Crisis team	6506	2.96 (2.77-3.16) ***	1.69 (1.57-1.83) ***
Never seen at A & E~	13389	Reference Group	
Ever seen at A & E~	6621	1.69 (1.58-1.80) ***	0.97 (0.90-1.04)
Never referred to Assertive Outreach	18977	Reference Group	
Ever referred to Assertive Outreach	1033	1.51 (1.32-1.72) ***	0.94 (0.81-1.09)
**Forensic History**			
No forensic history reported	18137	Reference Group	
Forensic History reported	1873	1.70 (1.53-1.88) ***	1.07 (0.96-1.20)

**p<.05; **p<.01; ***p<.001*; IMD= Index of Multiple Deprivation, 1=least deprived, 10=most deprived; ~ Seen at A & E due to mental health emergency; # For brevity reference groups are omitted. Reference groups are a non-affirmative response to the item. The *n* for the reference group is the number of people included in the analysis (N=20010) – the number of people with an affirmative response.
Step 1 Adjusted for Ethnicity + Gender + Age + IMD decile + Marital Status + diagnosis: psychosis/bipolar
Step 2 Adjusted for Ethnicity + Gender + Age + IMD decile + Marital Status + diagnosis: psychosis/bipolar + Substance use diagnosis + inpatient admittance + treated under the MHA + Structured risk assessment items (entered separately) + Referred to crisis team + Treated at A & E + Referred to assertive outreach + forensic history

For peer review only

For peer review only - http://bmjopen.bmj.com/site/about/guidelines.xhtml

Ethnicity and impact on the receipt of CBT

Table 3
Crude and adjusted associations from logistic regression models for at least sixteen recorded sessions of CBT

Variable	N	Odds Ratio (95% Confidence Interval) Crude Associations	Step 1	Step 2
**Ethnicity**				
White British	2456	Reference group		
Irish	137	1.03 (0.70-1.50)	1.02 (0.70-1.50)	1.05 (0.71-1.55)
Black African	682	0.78 (0.64-0.95) *	0.77 (0.63-0.95) *	0.86 (0.69-1.06)
Black Caribbean	1524	0.77 (0.67-0.90) **	0.76 (0.65-0.89) **	0.83 (0.71-0.98) *
South Asian	178	0.98 (0.70-1.38)	0.99 (0.72-1.39)	1.03 (0.73-1.47)
**Gender**				
Female	2485	Reference group		
Male	2492	0.99 (0.87-1.12)	0.98 (0.86-1.11)	1.05 (0.91-1.20)
**Age** (years)		1.00 (1.00-1.01)	1.00 (1.00-1.01)	1.00 (1.00-1.01)
**Area level deprivation**				
IMD decile (per tenth)		0.99 (0.97-1.01)	1.00 (0.97 1.02)	0.99 (0.97-1.01)
**Marital Status**				
In relationship	639	Reference group		
Single	4338	1.07 (0.88-1.29)	1.11 (0.91-1.36)	1.21 (0.98-1.48)
**Diagnosis**				
Psychosis	3645	Reference group		
Bipolar affective disorder	1332	0.95 (0.83-1.10)	0.90 (0.77-1.04)	0.86 (0.74-1.01)
**Comorbid substance misuse**				
No previous substance misuse diagnosis	4090	Reference group		
Lifetime comorbid substance misuse diagnosis	887	0.81 (0.69-0.97) *		0.79 (0.66-0.96) *
**Admission**				
No previous admission	1622	Reference Group		
Inpatient Admission ever	3355	0.74 (0.65-0.85) ***		1.06 (0.86-1.31)
**Treatment under Mental Health Act**				
Never treated under Mental Health Act	2429	Reference Group		
Ever treated under the Mental Health Act	2548	0.70 (0.61-0.79) ***		0.86 (0.71-1.05)
**Structured risk assessment items#**				
History of Violence	2234	0.80 (0.71-0.91) **		0.93 (0.78-1.10)
Difficulty managing physical health	1237	0.94 (0.81-1.09)		1.01 (0.85-1.20)
History of non-adherence	2382	0.83 (0.73-0.95) **		0.91 (0.77-1.08)
History of Suicide Attempt	1589	1.39 (1.22-1.59) ***		1.33 (1.09-1.61) **
Lethal means used in	887	1.36 (1.16-1.60) ***		1.01 (0.80-1.27)

For peer review only - http://bmjopen.bmj.com/site/about/guidelines.xhtml

suicide attempt			
Reported plans to end life	382	1.54 (1.23-1.92) ***	1.33 (1.01-1.73) *
Suicidal ideation	961	1.38 (1.18-1.61) ***	1.10 (0.89-1.35)
Feelings of hopelessness	1287	1.32 (1.14-1.52) ***	1.01 (0.82-1.23)
High level of distress	2000	1.22 (1.07-1.39) **	1.22 (1.03-1.44) *
No feelings of control	1337	1.24 (1.08-1.43) **	1.09 (0.90-1.31)
**Referred/seen by other team**			
Never referred to Crisis team	2459	Reference Group	
Ever referred to the Crisis team	2518	1.27 (1.12-1.44) ***	1.34 (1.14-1.56) ***
Never seen at A & E~	2918	Reference Group	
Ever seen at A & E~	2059	0.96 (0.84-1.09)	0.93 (0.80-1.08)
Never referred to Assertive Outreach	4636	Reference Group	
Ever referred to Assertive Outreach	341	0.67 (0.51-0.89) **	0.81 (0.60-1.08)
**Forensic History**			
No forensic history reported	4326	Reference Group	
Forensic History reported	651	0.80 (0.66-0.98) *	0.86 (0.69-1.06)
**Context of first CBT session**			
First CBT as outpatient	3493	Reference Group	
First CBT as inpatient	1484	0.35 (0.29-0.41) ***	0.35 (0.29-0.42) ***

p<.05;p<.01;***p<.001;* IMD= Index of Multiple Deprivation, 1=least deprived, 10=most deprived ; ~ Seen at A & E due to mental health emergency; # For brevity reference groups are omitted. Reference groups are a non-affirmative response to the item. The *n* for the reference group is the number of people included in the analysis (N=4977) – the number of people with an affirmative response.

Step 1 Adjusted for Ethnicity + Gender + Age + IMD decile + Marital Status + diagnosis: psychosis/bipolar

Step 2 Adjusted for Ethnicity + Gender + Age + IMD decile + Marital Status + diagnosis: psychosis/bipolar + Substance use diagnosis + inpatient admittance + treated under the MHA + Structured risk assessment items (entered separately) + Referred to crisis team + Treated at A & E + Referred to assertive outreach + forensic history + First CBT as inpatient

For peer review only

Ethnicity and impact on the receipt of CBT

Table 4
Crude and adjusted associations from logistic regression models for at least one recorded session of CBT as an inpatient

Variable	N	Odds Ratio (95% Confidence Interval)		
		Crude Associations	Step 1	Step 2
**Ethnicity**				
White British	4000	Reference Group		
Irish	232	0.95 (0.69-1.32)	1.02 (0.73-1.41)	0.99 (0.71-1.39)
Black African	1734	0.82 (0.71-0.95) **	0.80 (0.69-0.93) **	0.76 (0.65-0.89) **
Black Caribbean	3132	0.93 (0.83-1.05)	0.91 (0.80-1.02)	0.83 (0.73-0.94) **
South Asian	319	0.82 (0.62-1.10)	0.83 (0.62-1.12)	0.86 (0.64-1.16)
**Gender**				
Female	4390	Reference group		
Male	5027	0.93 (0.84-1.03)	0.89 (0.80-0.99) *	0.87 (0.79-0.97) *
**Age (years)**		0.99 (0.99-1.00) ***	0.99 (0.99-1.00) ***	0.99 (0.99-0.99) ***
**Area level deprivation**				
IMD decile (per tenth)		0.97 (0.95-0.99) **	0.97 (0.96-0.99) **	0.97 (0.95-0.99) **
**Marital Status**				
In relationship	1234	Reference group		
Single	8183	1.24 (1.06-1.45) **	1.19 (1.02-1.40) *	1.08 (0.91-1.27)
**Diagnosis**				
Psychosis	7114	Reference group		
Bipolar affective disorder	2303	0.97 (0.86-1.09)	0.94 (0.83-1.06)	1.02 (0.90-1.16)
**Comorbid substance misuse**				
No previous substance misuse diagnosis	7456	Reference group		
Lifetime comorbid substance misuse diagnosis	1961	1.05 (0.93-1.19)		0.88 (0.77-1.00)
**Treatment under Mental Health Act**				
No treatment under Mental Health Act	2506	Reference Group		
Ever treated under Mental Health Act	6911	1.56 (1.38-1.76) ***		1.39 (1.21-1.59) ***
**Structured risk assessment items#**				
History of Violence	4914	1.56 (1.41-1.73) ***		1.13 (1.00-1.28) *
Difficulty managing physical health	2720	1.59 (1.44-1.77) ***		1.34 (1.19-1.51) ***
History of Non-adherence	5161	1.66 (1.50-1.84) ***		1.24 (1.09-1.41) **
History of Suicide Attempt	2879	1.61 (1.46-1.79) ***		1.17 (1.00-1.35) *
Lethal means used in suicide attempt	1612	1.56 (1.38-1.77) ***		1.02 (0.86-1.20)
Plans to end life	754	1.66 (1.41-1.96) ***		1.09 (0.89-1.32)
Suicidal ideation	1684	1.66 (1.47-1.87) ***		1.14 (0.97-1.33)
Feelings of hopelessness	2218	1.66 (1.48-1.85) ***		1.08 (0.93-1.25)

For peer review only - http://bmjopen.bmj.com/site/about/guidelines.xhtml

Ethnicity and impact on the receipt of CBT

High level of distress	3747	1.82 (1.65-2.02) ***	1.37 (1.22-1.54) ***
No feelings of control	2370	1.68 (1.51-1.87) ***	1.08 (0.94-1.24)
**Referred/seen by other team**			
Never referred to Crisis team	4217	Reference Group	
Ever referred to the Crisis team	5200	1.08 (0.97-1.19)	0.90 (0.80-1.00) *
Never seen at A & E~	4981	Reference Group	
Ever seen at A & E~	4436	1.22 (1.10-1.34) ***	1.11 (1.00-1.23)
Never referred to Assertive Outreach	8633	Reference Group	
Ever referred to Assertive Outreach	784	1.45 (1.23-1.71) ***	1.18 (0.99-1.41)
**Forensic History**			
No forensic history reported	7936	Reference Group	
Forensic History reported	1481	1.11 (0.97-1.27)	1.02 (0.89-1.18)

p<.05;p<.01;***p<.001;* IMD= Index of Multiple Deprivation, 1=least deprived, 10=most deprived; ~ Seen at A & E due to mental health emergency; # For brevity reference groups are omitted. Reference groups are a non-affirmative response to the item. The *n* for the reference group is the number of people included in the analysis (N=9417) – the number of people with an affirmative response.

Step 1 Adjusted for Ethnicity + Gender + Age + IMD decile + Marital Status + diagnosis: psychosis/bipolar

Step 2 Adjusted for Ethnicity + Gender + Age + IMD decile + Marital Status + diagnosis: psychosis/bipolar + Substance use diagnosis + treated under the MHA + Structured risk assessment items (entered separately) + Referred to crisis team + Treated at A & E + Referred to assertive outreach + forensic history

For peer review only

For peer review only - http://bmjopen.bmj.com/site/about/guidelines.xhtml

Ethnicity and impact on the receipt of CBT

For peer review only

For peer review only - http://bmjopen.bmj.com/site/about/guidelines.xhtml

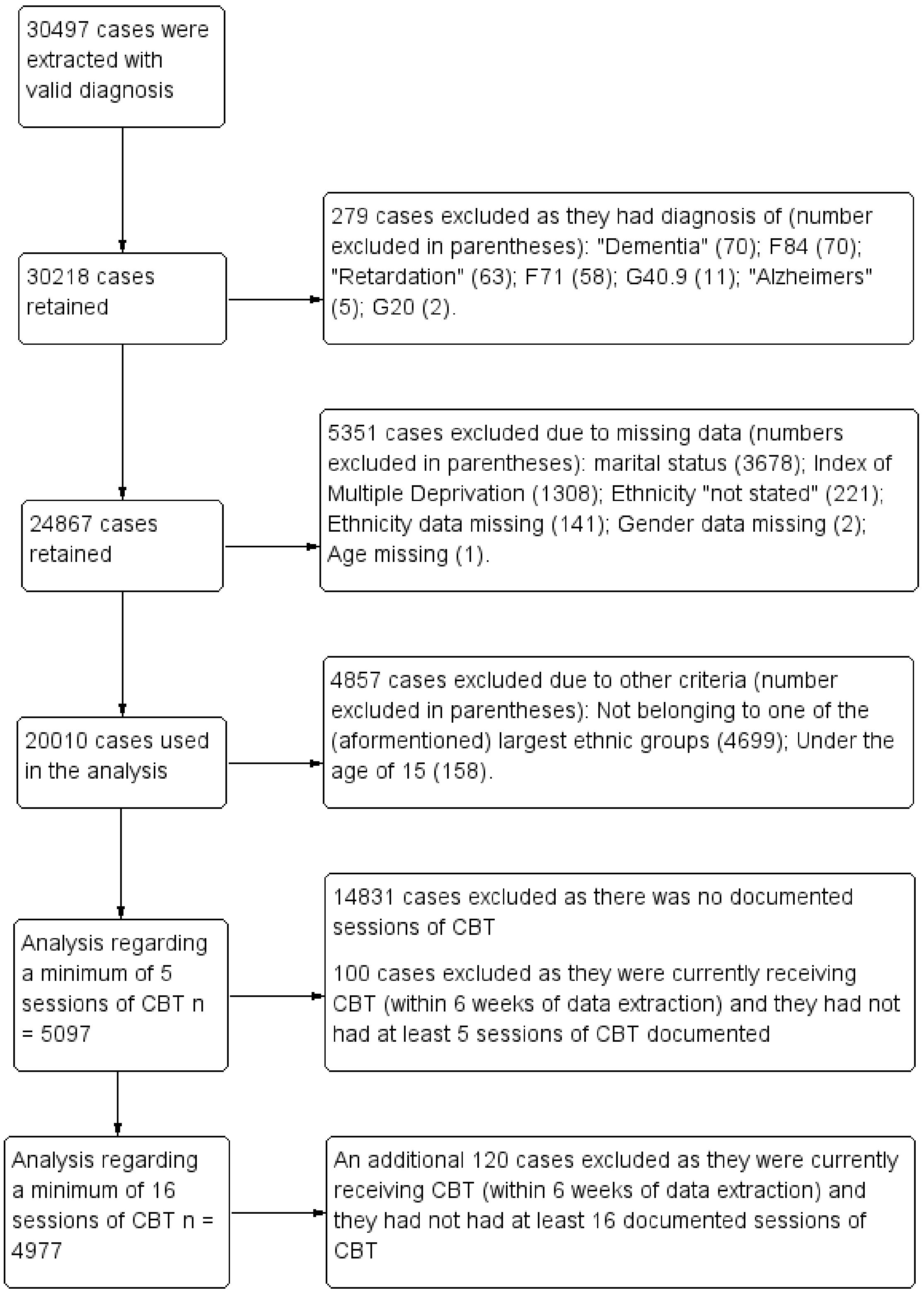

Figure 1. Demonstrating the flow of included cases

Supplementary Data Contents

Supplementary Data 1

Supplementary Data 1 contains further information regarding treatment, risk, and severity including items from a structured risk assessment. The table also contains tests of difference between the different ethnic groups.

Supplementary Data 2

Supplementary Data 2 contains information about CBT treatment received. The table displays the relevant proportions for each ethnic group and tests of difference.

Supplementary Data 3

Supplementary Data 3 contains crude estimates and adjusted multivariable logistic regression models regarding ethnicity and having a minimum of 5 sessions of CBT.

Supplementary Data 4

Supplementary Data 4 contains crude estimates and adjusted multivariable logistic regression models regarding ethnicity and reported receipt of CBT as an outpatient.

Supplementary Data 5

Supplementary Data 5 contains crude estimates and adjusted multivariable logistic regression models regarding ethnicity and reported receipt of CBT in the unstructured clinical notes (i.e. just using data derived from free text not structured fields).

.

For peer review only

Supplementary Data 6

Supplementary Data 6 contains crude estimates and adjusted multivariable logistic regression models regarding ethnicity and reported receipt of CBT which are adjusted for the effect of time.

For peer review only

Supplementary Data 1

Table 1
Information about clients' treatment and risk assessment

		White British		Irish		African		Caribbean		South Asian		Total			
		N	%	N	%	N	%	n	%	n	%	N	%	Chi²*	*p*-value
	Ever treated under Mental Health Act	2575	24.8	155	27.2	1492	53.0	2648	48.3	236	31.5	7106	35.5	1300.0	<*.001*
	Inpatient admission ever	4000	38.5	232	40.7	1734	61.6	3132	57.1	319	42.6	9417	47.1	783.0	<*.001*
	Forensic History#	836	8.0	44	7.7	260	9.2	700	12.8	33	4.4	1873	9.4	119.9	<*.001*
Structured risk assessment items	History of violence	2324	22.4	140	24.6	1086	38.6	2491	45.4	175	23.4	6216	31.1	1000.0	<*.001*
	Difficulty managing physical health	1645	15.8	98	17.2	513	18.2	1240	22.6	126	16.8	3622	18.1	113.0	<*.001*
	History of non-adherence	2320	22.3	150	26.3	1177	41.8	2579	47.1	199	26.6	6425	32.1	1200.0	<*.001*
	History of suicide attempt	2062	19.8	119	20.9	445	15.8	1045	19.1	87	11.6	3758	18.8	51.2	<*.001*
	Lethal means used in suicide attempt	1157	56.1	67	56.3	230	51.7	531	50.8	41	47.1	2026	53.9	44.1	<*.001*

For peer review only

Current plans to end life	524	5.0	25	4.4	109	3.9	186	3.4	19	2.5	863	4.3	31.7	*<.001*
Current suicidal ideation	1126	10.8	73	12.8	256	9.1	526	9.6	60	8.0	2041	10.2	18.7	*<.01*
Hopelessness	1481	14.3	93	16.3	398	14.1	797	14.5	81	10.8	2850	14.2	9.6	*.047*
High levels of distress	1986	19.1	129	22.6	808	28.7	1595	29.1	148	19.8	4666	23.3	256.3	*<.001*
No perception of control	1404	13.5	86	15.1	442	15.7	950	17.3	90	12.0	2972	14.9	47.8	*<.001*
Crisis team ever	2642	25.4	145	25.4	1241	44.1	2209	40.3	269	35.9	6506	32.5	577.8	*<.001*
Assertive outreach ever	496	4.8	10	1.8	149	5.3	348	6.3	30	4.0	1033	5.2	34.7	*<.001*
A&E ever~	3172	30.5	191	33.5	1125	39.9	1879	34.3	254	33.9	6621	33.1	94.4	*<.001*

Forensic history noted in the clinical records includes self-reported and reports by professionals from different organisations; ~ Seen at A & E due to mental health; * All degrees of freedom = 4

For peer review only

Supplementary Data 2

Table 2

Information about CBT and the relevant proportions by ethnicity

	White British		Irish		African		Caribbean		South Asian		Total		Kruskal -Wallis *H*, Chi^2 *	*p*-value
	N	%	N	%	n	%	n	%	n	%	N	%		
CBT ever	2541	24.5	139	24.4	722	25.6	1613	29.4	182	24.3	5197	26.0	48.6	<.001
CBT on-going	160	6.3	9	6.5	66	9.1	141	8.7	10	5.5	386	7.4	24	<.001
Sessions: median (IQR)#	6 (14)		8 (15)		4 (12)		5 (11)		6 (14)		5 (13)		37.2	<.001
CBT inpatient ever	833	8.0	47	8.3	318	11.3	644	11.8	57	7.6	1899	9.5	47.1	<.001
Inpatient sessions: median (IQR) ##	2 (5)		2 (3)		2 (2)		2 (3)		1 (3)		2 (3)		22.4	<.001
CBT outpatient ever	2011	19.4	113	19.8	514	18.3	1197	21.8	147	19.6	3982	19.9	14.2	<.01
Outpatient sessions: median (IQR) ###	8 (16)		8 (16)		6 (15)		6 (14)		8 (16)		7 (15)		38.1	<.001
% of sessions Face to Face	96.5		97.8		97.0		96.6		98.3		96.7			

For peer review only

% of sessions one to one	94.7		91.7		92.4		93.1		96.5		93.9			
1st CBT as inpatient##	670	26.4	37	26.6	262	36.3	537	33.3	51	28.0	1557	30.0	68.2	<.001
>= 5 sessions~	1477	58.9	82	59.4	344	48.9	818	52.3	101	56.1	2822	55.4	31.4	<.001
>= 16 sessions~	686	27.9	39	28.7	158	23.2	352	23.1	49	27.5	1284	25.8	14.9	<.01

* Kruskal-Wallis H non parametric test for ranked data used to determine the Chi^2 value , all degrees of freedom = 4; # Including only the people who had received CBT; ## Including only people who have received CBT as an inpatient; ### Including only people who have received CBT as an outpatient

~ Only includes people who had ever received CBT and people who had not received CBT within 6 weeks of data extraction if they did not meet the criteria (i.e. people with <5 or <16 sessions who were currently receiving CBT)

For peer review only

Supplementary Data 3

Table 3
Crude and adjusted associations from logistic regression models for at least five recorded sessions of CBT (inpatient or outpatient)

Variable	N	Odds Ratio (95% Confidence Interval)		
		Crude Associations	Step 1	Step 2
**Ethnicity**				
White British	2509	Reference group		
Irish	139	1.01 (0.71-1.42)	1.02 (0.72-1.44)	1.02 (0.71-1.47)
Black African	704	0.67 (0.56-0.79) ***	0.68 (0.57-0.81) ***	0.76 (0.63-0.91) **
Black Caribbean	1565	0.77 (0.67-0.89) ***	0.79 (0.69-0.90) ***	0.88 (0.76-1.01)
South Asian	180	0.89 (0.66-1.21) ***	0.88 (0.64-1.19)	0.91 (0.66-1.25)
**Gender**				
Female	2543	Reference Group		
Male	2554	0.97 (0.87-1.09) ***	0.99 (0.88-1.11)	1.09 (0.96-1.23)
**Age (years)**		1.00 (1.00-1.00)	1.00 (1.00-1.00)	1.00 (1.00-1.00)
**Area level deprivation**				
IMD decile (per tenth)		0.97 (0.96-0.99) *	0.99 (0.97-1.01)	0.98 (0.96-1.00)*
**Marital Status**				
In relationship	654	Reference Group		
Single	4443	0.84 (0.71-0.99) *	0.87 (0.73-1.03)	0.95 (0.79-1.14)
**Diagnosis**				
Psychosis	3734	Reference Group		
Bipolar affective disorder	1363	1.14 (1.01-1.29) *	1.04 (0.92-1.19)	1.00 (0.87-1.15)
**Comorbid substance misuse**				
No previous substance misuse diagnosis	4191	Reference group		
Lifetime comorbid substance misuse diagnosis	906	0.78 (0.68-0.90) **		0.79 (0.68-0.93) **
**Admission**				
No previous admission	1650	Reference Group		
Inpatient admission ever	3447	0.64 (0.57-0.72) ***		1.05 (0.87-1.28)
**Treatment under Mental Health Act**				
No treatment under Mental Health Act	2473	Reference Group		
Ever treated under Mental Health Act	2624	0.63 (0.56-0.70) ***		0.91 (0.76-1.08)**
**Structured risk assessment items#**				
History of Violence	2288	0.71 (0.64-0.80) ***		0.88 (0.76-1.02)
Difficulty managing physical health	1257	0.81 (0.71-0.92) **		0.96 (0.83-1.12)
History of Non-adherence	2443	0.73 (0.66-0.82) ***		0.90 (0.77-1.05)

For peer review only

History of Suicide Attempt	1628	1.21 (1.08-1.37) **	1.23 (1.03-1.72) *
Lethal means used in suicide attempt	904	1.20 (1.04-1.39) *	0.98 (0.80-1.21)
Plans to end life	396	1.43 (1.16-1.77) **	1.33 (1.03-1.72) *
Suicidal ideation	991	1.30 (1.13-1.50) ***	1.13 (0.93-1.36)
Feelings of hopelessness	1318	1.20 (1.05-1.36) **	0.97 (0.81-1.16)
High level of distress	2059	1.13 (1.01-1.26) *	1.29 (1.11-1.49) **
No feelings of control	1366	1.12 (0.99-1.27)	1.06 (0.89-1.25)
**Referred/seen by other team**			
Never referred to Crisis team	2508	Reference Group	
Ever referred to the Crisis team	2589	1.13 (1.01-1.26) *	1.21 (1.05-1.38) **
Never seen at A & E~	2971	Reference Group	
Ever seen at A & E~	2126	0.96 (0.86-1.07)	1.00 (0.88-1.14)
Never referred to Assertive Outreach	4752	Reference Group	
Ever referred to Assertive Outreach	345	0.65 (0.52-0.80) ***	0.83 (0.65-1.05)
**Forensic History**			
No forensic history reported	4435	Reference Group	
Forensic History reported	662	0.72 (0.61-0.85) ***	0.81 (0.68-0.97) *
**Context of first CBT session**			
First CBT as outpatient	3584	Reference Group	
First CBT as inpatient	1513	0.33 (0.29-0.38) ***	0.34 (0.29-0.39) ***

p<.05;p<.01;***p<.001*; IMD= Index of Multiple Deprivation, 1=least deprived, 10=most deprived; ~ Seen at A & E due to mental health emergency; # For brevity reference groups are omitted. Reference groups are a non-affirmative response to the item. The *n* for the reference group is the number of people included in the analysis (N=5097) – the number of people with an affirmative response.

Step 1 Adjusted for Ethnicity + Gender + Age + IMD decile + Marital Status + diagnosis: psychosis/bipolar

Step 2 Adjusted for Ethnicity + Gender + Age + IMD decile + Marital Status + diagnosis: psychosis/bipolar + Substance use diagnosis + inpatient admittance + treated under the MHA + Structured risk assessment items (entered separately) + Referred to crisis team + Treated at A & E + Referred to assertive outreach + forensic history + First CBT as inpatient

For peer review only - http://bmjopen.bmj.com/site/about/guidelines.xhtml

Supplementary Data 4

Table 4

Crude and adjusted associations from logistic regression models for at least one recorded session of CBT as an outpatient

Variable	N	Odds Ratio (95% Confidence Interval) Crude Associations	Step 1	Step 2
**Ethnicity**				
White British	10393	Reference Group		
Irish	570	1.03 (0.84-1.27)	1.15 (0.94- 1.42)	1.10 (0.89- 1.37)
Black African	2817	0.90 (0.81-1.00)	0.83 (0.74 - 0.92) **	0.75 (0.67-0.84) ***
Black Caribbean	5481	1.11 (1.03-1.20) **	1.06 (0.98- 1.15)	0.95 (0.87- 1.04)
South Asian	749	1.00 (0.83-1.20)	0.98 (0.81-1.18)	0.97 (0.80-1.17)
**Gender**				
Female	9553	Reference Group		
Male	10457	0.85 (0.80-0.92) ***	0.82 (0.76-0.88) ***	0.85 (0.79-0.91) ***
**Age (years)**		0.99 (0.98-0.99) ***	0.98 (0.98-0.99) ***	0.99 (0.98-0.99) ***
**Area level deprivation**				
IMD decile (per tenth)		1.00 (0.99-1.01)	1.00 (0.99-1.02)	0.99 (0.98-1.00)
**Marital Status**				
In relationship	2913	Reference Group		
Single	17097	1.09 (0.98-1.20)	0.98 (0.88-1.08)	1.01 (0.91-1.13)
**Diagnosis**				
Psychosis	14497	Reference Group		
Bipolar	5513	1.09 (1.01-1.18) *	1.15 (0.94- 1.42)	1.07 (0.99-1.16)
**Comorbid substance misuse**				
No previous substance misuse diagnosis	16985	Reference group		
Lifetime comorbid substance misuse diagnosis	3025	1.15 (1.05-1.27) **		0.86 (0.78-0.95) **
**Admission**				
No previous admission	10593	Reference Group		
Inpatient Admission Ever	9417	1.69 (1.57-1.81) ***		1.13 (1.01-1.27) *
**Treatment under Mental Health Act#**				
No treatment under Mental Health Act	12904	Reference Group		
Ever treated under Mental Health Act	7106	1.40 (1.30-1.50) ***		0.75 (0.67-0.84) ***
**Structured risk assessment items#**				
History of Violence	6216	1.54 (1.43-1.65) ***		1.02 (0.93-1.13)
Difficulty managing physical health	3622	1.19 (1.09-1.29) ***		0.83 (0.75-0.92) **
History of Non-adherence	6425	1.69 (1.57-1.81) ***		1.22 (1.11-1.35) ***
History of Suicide Attempt	3758	2.25 (2.08-2.44) ***		1.38 (1.23-1.56) ***

For peer review only

Lethal means used in suicide attempt	2026	2.09 (1.89-2.31) ***	1.02 (0.88-1.17)
Plans to end life	863	2.01 (1.74-2.33) ***	0.79 (0.66-0.94) **
Suicidal ideation	2041	2.62 (2.38-2.89) ***	1.26 (1.11-1.44) ***
Feelings of hopelessness	2850	2.50 (2.30-2.73) ***	1.30 (1.15-1.47) ***
High level of distress	4666	2.36 (2.19-2.54) ***	1.50 (1.36-1.65) ***
No feelings of control	2972	2.33 (2.14-2.54) ***	1.21 (1.08-1.36) **
**Referred/seen by other team**			
Never referred to Crisis team	13504	Reference Group	
Ever referred to the Crisis team	6506	2.49 (2.32-2.67)***	2.02 (1.86-2.21) ***
Never seen at A & E~	13389	Reference Group	
Ever seen at A & E~	6621	1.36 (1.27-1.46)***	0.88 (0.81-0.96) **
Never referred to Assertive Outreach	18977	Reference Group	
Ever referred to Assertive Outreach	1033	0.92 (0.78-1.07)	0.71 (0.60-0.84) ***
**Forensic History**			
No forensic history reported	18137	Reference Group	
Forensic History reported	1873	1.45 (1.30-1.61) ***	1.15 (1.02-1.29) *
**Context of first CBT session**			
First CBT not as inpatient	18453	Reference Group	
First CBT as inpatient	1557	1.08 (0.95-1.23)	0.71 (0.62-0.81) ***

p<.05;p<.01;***p<.001;* IMD= Index of Multiple Deprivation, 1=least deprived, 10=most deprived; ~ Seen at A & E due to mental health emergency; # For brevity reference groups are omitted. Reference groups are a non-affirmative response to the item. The *n* for the reference group is the number of people included in the analysis (N=20010) – the number of people with an affirmative response.

Step 1 Adjusted for Ethnicity + Gender + Age + IMD decile + Marital Status + diagnosis: psychosis/bipolar

Step 2 Adjusted for Ethnicity + Gender + Age + IMD decile + Marital Status + diagnosis: psychosis/bipolar + Substance use diagnosis + inpatient admittance + treated under the MHA + Structured risk assessment items (entered separately) + Referred to crisis team + Treated at A & E + Referred to assertive outreach + forensic history + First CBT as an inpatient

For peer review only

Supplementary Data 5

Sensitivity Analysis

Table 5
Crude and adjusted associations from logistic regression models for at least one recorded session of CBT (inpatient or outpatient) in the unstructured clinical notes

Variable	N	Odds Ratio (95% Confidence Interval)		
		Crude Associations	Step 1	Step 2
**Ethnicity**				
White British	10393	Reference group		
Irish	570	0.88 (0.62-1.26)	1.07 (0.75-1.53)	1.04 (0.72-1.49)
Black African	2817	0.85 (0.71-1.01)	0.79 (0.66-0.95)*	0.76 (0.63-0.92) **
Black Caribbean	5481	0.96 (0.85-1.10)	0.95 (0.82-1.09)	0.90 (0.78-1.04)
South Asian	749	0.72 (0.52-1.02)	0.70 (0.50-0.99)*	0.89 (0.49-0.98)
**Gender**				
Female	9553	Reference group		
Male	10457	0.84 (0.75-0.94) **	0.83 (0.74-0.93) **	0.89 (0.79-1.00) *
**Age (years)**		0.98 (0.97-0.98) ***	0.98 (0.97-0.98) ***	0.98 (0.97-0.98) ***
**Area level deprivation**				
IMD decile (per tenth)		0.98 (0.96-1.00)*	0.99 (0.97-1.01)	0.98 (0.96-1.00)
**Marital Status**				
In relationship	2913	Reference group		
Single	17097	1.02 (0.87-1.21)	0.88 (0.74-1.04)	0.91 (0.77-1.09)
**Diagnosis**				
Psychosis	14497	Reference Group		
Bipolar affective disorder	5513	1.27 (1.12-1.43)***	1.13 (0.99-1.29)	1.17 (1.03-1.34)*
**Comorbid substance misuse**				
No previous substance misuse diagnosis	16985	Reference group		
Lifetime comorbid substance misuse diagnosis	3025	0.96 (0.82-1.13)		0.75 (0.64-0.89)**
**Admission**				
No previous admission	10593	Reference Group		
Inpatient Admission Ever	9417	1.65 (1.47-1.85) ***		1.60 (1.35-1.90) ***
**Treatment under Mental Health Act**				
No treatment under Mental Health Act	12904	Reference Group		
Ever treated under Mental Health Act	7106	1.19 (1.06-1.33) **		0.67 (0.57-0.80)***
**Structured risk assessment items#**				
History of Violence	6216	1.16 (1.03-1.31) *		0.84 (0.72-0.99)*

For peer review only

Difficulty managing physical health	3622	1.01 (0.87-1.17)	0.92 (0.78-1.09)
History of Non-adherence	6425	1.29 (1.15-1.45) ***	1.12 (0.95-1.31)
History of Suicide Attempt	3758	1.91 (1.69-2.17) ***	1.23 (1.02-1.50) *
Lethal means used in suicide attempt	2026	2.00 (1.72-2.32) ***	1.20 (0.97-1.50)
Plans to end life	863	2.01 (1.62-2.50) ***	0.90 (0.69-1.16)
Suicidal ideation	2041	2.42 (2.09-2.79) ***	1.43 (1.17-1.74) ***
Feelings of hopelessness	2850	1.95 (1.71-2.24) ***	1.11 (0.91-1.35)
High level of distress	4666	1.90 (1.69-2.14) ***	1.43 (1.17-1.74) ***
No feelings of control	2972	1.76 (1.54-2.02) ***	1.02 (0.85-1.23)
**Referred/seen by other team**			
Never referred to Crisis team	13504	Reference Group	
Ever referred to the Crisis team	6506	1.80 (1.61-2.02) ***	1.45 (1.26-1.66) ***
Never seen at A & E~	13389	Reference Group	
Ever seen at A & E~	6621	1.16 (1.03-1.30) *	0.76 (0.67-0.87)***
Never referred to Assertive Outreach	18977	Reference Group	
Ever referred to Assertive Outreach	1033	0.75 (0.56-1.00) *	0.61 (0.46-0.82)**
**Forensic History**			
No forensic history reported	18137	Reference Group	
Forensic History reported	1873	1.11 (0.92-1.34)	0.96 (0.78-1.17)

**p<.05; **p<.01; ***p<.001*; IMD= Index of Multiple Deprivation, 1=least deprived, 10=most deprived; ~ Seen at A & E due to mental health emergency; # For brevity reference groups are omitted. Reference groups are a non-affirmative response to the item. The *n* for the reference group is the number of people included in the analysis (N=20010) – the number of people with an affirmative response.

Step 1 Adjusted for Ethnicity + Gender + Age + IMD decile + Marital Status + diagnosis: psychosis/bipolar

Step 2 Adjusted for Ethnicity + Gender + Age + IMD decile + Marital Status + diagnosis: psychosis/bipolar + Substance use diagnosis + inpatient admittance + treated under the MHA + Structured risk assessment items (entered separately) + Referred to crisis team + Treated at A & E + Referred to assertive outreach + forensic history

For peer review only

Supplementary Data 6

Time analysis

Table 6

Crude and adjusted associations from logistic regression models for at least one recorded session of CBT (inpatient or outpatient) adjusting for time

Variable	N	Odds Ratio (95% Confidence Interval)		
		Crude Associations#	Step 1	Step 2
**Ethnicity**				
White British	10393	Reference group		
Irish	570	0.97 (0.80-1.19)	1.10 (0.90-1.35)	1.04 (0.84-1.28)
Black African	2817	1.06 (0.96-1.17)	0.93 (0.84-1.03)	0.72 (0.65-0.81) ***
Black Caribbean	5481	1.27 (1.18-1.36) ***	1.16 (1.08-1.25) ***	0.92 (0.85-1.00)
South Asian	749	1.00 (0.84-1.19)	0.97 (0.82-1.16)	0.93 (0.77-1.12)
**Gender**				
Female	9553	Reference group		
Male	10457	0.88 (0.83-0.94)***	0.81 (0.76-0.87) ***	0.83 (0.78-0.89) ***
**Age (years)**		0.98 (0.98-0.98) ***	0.98 (0.98-0.98) ***	0.98 (0.98-0.99) ***
**Area level deprivation**				
IMD decile (per tenth)		1.01 (1.00-1.02)	1.01 (0.99-1.02)	0.99 (0.98-1.00)
**Marital Status**				
In relationship	2913	Reference group		
Single	17097	1.22 (1.11-1.34) ***	1.56 (0.95-1.15)	1.05 (0.95-1.17)
**Diagnosis**				
Psychosis	14497	Reference Group		
Bipolar affective disorder	5513	0.96 (0.89-1.03)	0.94 (0.87-1.01)	1.01 (0.93-1.09)
**Comorbid substance misuse**				
No previous substance misuse diagnosis	16985	Reference group		
Lifetime comorbid substance misuse diagnosis	3025	1.31 (1.20-1.43) ***		0.85 (0.77-0.94)**
**Admission**				
No previous admission	10593	Reference Group		
Inpatient Admission Ever	9417	3.16 (2.96-3.38) ***		1.74 (1.56-1.93) ***
**Treatment under Mental Health Act**				
No treatment under Mental Health Act	12904	Reference Group		
Ever treated under Mental Health Act	7106	2.51 (2.35-2.68) ***		0.98 (0.88-1.09)
**Structured risk assessment items#**				
History of Violence	6216	2.26 (2.12-2.42) ***		1.08 (0.99-1.19)

For peer review only

Difficulty managing physical health	3622	1.68 (1.55-1.81) ***		0.96 (0.87-1.05)
History of Non-adherence	6425	2.51 (2.35-2.68) ***		1.24 (1.13-1.36) ***
History of Suicide Attempt	3758	2.79 (2.59-3.01) ***		1.35 (1.21-1.52) ***
Lethal means used in suicide attempt	2026	2.61 (2.37-2.86) ***		1.04 (0.91-1.19)
Plans to end life	863	2.64 (2.30-3.03) ***		0.82 (0.70-0.97) *
Suicidal ideation	2041	3.26 (2.97-3.58) ***		1.26 (1.11-1.43) ***
Feelings of hopelessness	2850	3.04 (2.80-3.30) ***		1.25 (1.11-1.41) ***
High level of distress	4666	3.21 (2.99-3.44) ***		1.55 (1.41-1.69) ***
No feelings of control	2972	2.98 (2.75-3.23) ***		1.20 (1.08-1.34) **
**Referred/seen by other team**				
Never referred to Crisis team	13504	Reference Group		
Ever referred to the Crisis team	6506	2.93 (2.74-3.13) ***		1.67 (1.54-1.81) ***
Never seen at A & E~	13389	Reference Group		
Ever seen at A & E~	6621	1.74 (1.63-1.86) ***		0.99 (0.91-1.06)
Never referred to Assertive Outreach	18977	Reference Group		
Ever referred to Assertive Outreach	1033	1.46 (1.27-1.66) ***		0.92 (0.80-1.06)
**Forensic History**				
No forensic history reported	18137	Reference Group		
Forensic History reported	1873	1.62 (1.46-1.79) ***		1.02 (0.91-1.14)
**Time point diagnosis given##**				
Psychosis/bipolar affective disorder diagnosis before midpoint of study period	13518	Reference category		
Psychosis/bipolar affective disorder diagnosis after midpoint of study period	6492	0.76 (0.71-0.82) ***	0.64 (0.59-0.69) ***	0.77 (0.71-0.83) ***

*p<.05; **p<.01; ***p<.001*; # including time; IMD= Index of Multiple Deprivation, 1=least deprived, 10=most deprived; ~ Seen at A & E due to mental health emergency; # For brevity reference groups are omitted. Reference groups are a non-affirmative response to the item. The *n* for the reference group is the number of people included in the analysis (N=20010) – the number of people with an affirmative response.
##midpoint of study period was 16/4/2012
Step 1 Adjusted for Ethnicity + Gender + Age + IMD decile + Marital Status + diagnosis: psychosis/bipolar + Psychosis/bipolar affective disorder diagnosis after 16/04/12
Step 2 Adjusted for Ethnicity + Gender + Age + IMD decile + Marital Status + diagnosis: psychosis/bipolar + Substance use diagnosis + inpatient admittance + treated under the MHA + Structured risk assessment items (entered separately) + Referred to crisis team + Treated at A & E + Referred to assertive outreach + forensic history + Psychosis/bipolar affective disorder diagnosis after 16/04/12

For peer review only

For peer review only

STROBE Statement—Checklist of items that should be included in reports of cohort studies

**Title and abstract** 1	(a) Indicate the study's design with a commonly used term in the title or the abstract	p.1
	(b) Provide in the abstract an informative and balanced summary of what was done and what was found	pp.3-4
**Introduction**		
Background/rationale 2	Explain the scientific background and rationale for the investigation being reported	pp.5-7
Objectives 3	State specific objectives, including any prespecified hypotheses	p.7
**Methods**		
Study design 4	Present key elements of study design early in the paper	pp.7-8
Setting 5	Describe the setting, locations, and relevant dates, including periods of recruitment, exposure, follow-up, and data collection	p.7 & p.9
Participants 6	(a) Give the eligibility criteria, and the sources and methods of selection of participants. Describe methods of follow-up	pp.8-9
	(b) For matched studies, give matching criteria and number of exposed and unexposed	n/a
Variables 7	Clearly define all outcomes, exposures, predictors, potential confounders, and effect modifiers. Give diagnostic criteria, if applicable	pp.10-11
Data sources/ measurement 8	For each variable of interest, give sources of data and details of methods of assessment (measurement). Describe comparability of assessment methods if there is more than one group	pp.10-11
Bias 9	Describe any efforts to address potential sources of bias	pp.15-16
Study size 10	Explain how the study size was arrived at	Figure 1
Quantitative variables 11	Explain how quantitative variables were handled in the analyses. If applicable, describe which groupings were chosen and why	pp.10-11 & p.12
Statistical methods 12	(a) Describe all statistical methods, including those used to control for confounding	p.12
	(b) Describe any methods used to examine subgroups and interactions	pp.14-15
	(c) Explain how missing data were addressed	p.12
	(d) If applicable, explain how loss to follow-up was addressed	n/a
	(e) Describe any sensitivity analyses	pp.15-16
**Results**		
Participants 13	(a) Report numbers of individuals at each stage of study—eg numbers potentially eligible, examined for eligibility, confirmed eligible, included in the study, completing follow-up, and analysed	Figure 1
	(b) Give reasons for non-participation at each stage	Figure 1
	(c) Consider use of a flow diagram	Figure 1
Descriptive data 14	(a) Give characteristics of study participants (eg demographic, clinical, social) and information on exposures and potential confounders	p.12 & Table 1 & Supp. Data
	(b) Indicate number of participants with missing data for each variable of interest	n/a
	(c) Summarise follow-up time (eg, average and total amount)	n/a

For peer review only

Outcome data 15	Report numbers of outcome events or summary measures over time	Supp. Data 2
Main results 16	(a) Give unadjusted estimates and, if applicable, confounder-adjusted estimates and their precision (eg, 95% confidence interval). Make clear which confounders were adjusted for and why they were included	Tables 2-4 (pp.35-40)
	(b) Report category boundaries when continuous variables were categorized	n/a
	(c) If relevant, consider translating estimates of relative risk into absolute risk for a meaningful time period	n/a
Other analyses 17	Report other analyses done—eg analyses of subgroups and interactions, and sensitivity analyses	pp.15-16
**Discussion**		
Key results 18	Summarise key results with reference to study objectives	p.16
Limitations 19	Discuss limitations of the study, taking into account sources of potential bias or imprecision. Discuss both direction and magnitude of any potential bias	pp.17-18
Interpretation 20	Give a cautious overall interpretation of results considering objectives, limitations, multiplicity of analyses, results from similar studies, and other relevant evidence	pp.19-20
Generalisability 21	Discuss the generalisability (external validity) of the study results	p.17
**Other information**		
Funding 22	Give the source of funding and the role of the funders for the present study and, if applicable, for the original study on which the present article is based	p.21

For peer review only
